# Iron-Catalyzed Decarboxylative Sulfinylation of Alkyl Carboxylic Acids

**DOI:** 10.1021/acs.orglett.5c03100

**Published:** 2025-09-04

**Authors:** Matthew Southern, Christopher Pearce, Michael C. Willis

**Affiliations:** † Department of Chemistry, 6396 University of Oxford, Chemistry Research Laboratory, Mansfield Road, Oxford, OX1 3TA, U.K.; ‡ Sygnature Discovery, Bio City, Pennyfoot St, Nottingham NG1 1GR, U.K.

## Abstract

Sulfinamides, sulfonamides, and sulfonimidamides are valuable motifs in medicinal chemistry, yet methods to synthesize alkyl variants from simple, readily available feedstocks remain scarce. In this report, we detail the synthesis of these three distinct sulfur functional groups, using readily available and structurally diverse alkyl carboxylic acids as the starting materials. The method harnesses alkyl radical generation from carboxylic acids using commercial iron salts and visible light irradiation, in combination with commercial sulfinylamine reagents, to deliver alkyl sulfinamide products. The method is operationally simple and scalable, exhibits broad functional group tolerance, and is translatable to continuous-flow synthesis. Furthermore, it facilitates late-stage diversification of complex molecules, highlighting its potential utility in medicinal chemistry applications.

## Graphical abstract

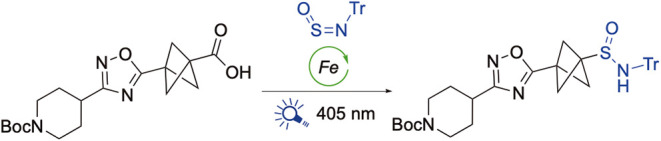

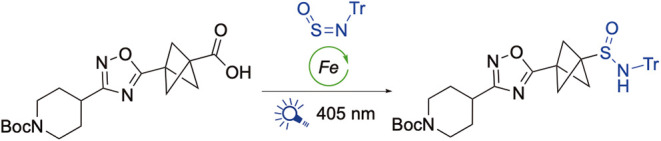

High-oxidation-state sulfur­(VI) functional groups, including sulfonamides and their aza-analogues, sulfonimidamides, are privileged motifs in medicinal chemistry, prized for their stability, polarity, and capacity to introduce three-dimensional vectors into molecules, aligning with modern structure–activity relationship (SAR) exploration.^1^ Sulfonimidamides, in particular, offer distinct synthetic and biological advantages by expanding exit vectors from the sulfur center and enabling novel interactions in protein binding sites, which can enhance potency and selectivity (Scheme 1a).^2,3^

**Scheme 1:**
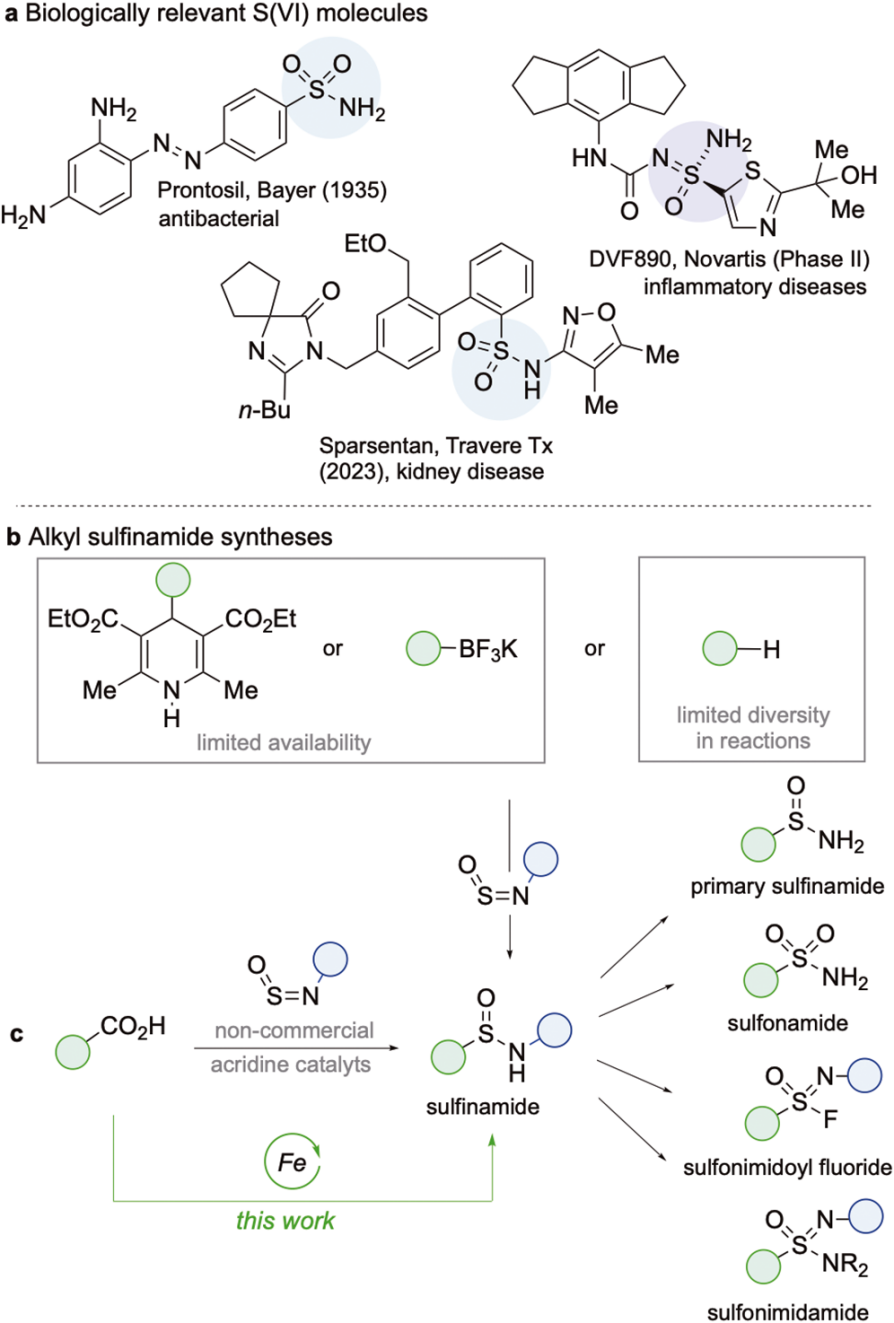
Alkyl Sulfinamide Synthesis, and This Work

Recently, several efficient routes to sp^3^-linked sulfinamides have emerged. These key intermediates^4^ en route to sulfonimidamides are accessible from commercial sulfinylamine reagents^5^ (R-NSO) in combination with preformed organometallic nucleophiles, enabling the synthesis of sulfonamides and sulfonimidamides (Scheme 1b).^6^ Sulfinylamines have also been combined with carbon-centered radicals; Bolm demonstrated that aryl radicals generated from diazonium salts can install sulfur­(VI) groups,^7^ and Li showed that radical species derived from Hantzsch esters transfer alkyl groups onto sulfinylamines.^8^ While effective, these strategies rely on either preformed organometallics, which show poor functional group tolerance, on high-energy diazonium salts, or specialized radical precursors, all of which are ill-suited for discovery chemistry applications because of safety concerns and/or limited reagent availability. Iron-catalyzed radical C–H activation of sp^3^ centers has been used to install sulfur­(VI) groups onto aliphatic scaffolds, although the structural diversity and functional group tolerance using this approach is limited.^9^ Recently, the Li^10^ and Sahoo laboratories and have shown that alkyl BF_3_K salts can act as radical precursors for sulfonamide synthesis,^11^ broadening the substrate scope of sulfur­(VI) installation. Ye and co-workers have reported an electrochemical synthesis, using sulfenamides as substrates.^12^

The use of abundant, stable, and broadly available alkyl carboxylic acids as radical precursors offers a solution to many of the limitations seen with earlier sulfinamide syntheses. Recent work from the Willis and Larioniov laboratories^13^ has shown that decarboxylative routes to sulfinamides, combining alkyl carboxylic acids and sulfinylamines, are efficient methods for their synthesis. Despite the success of these methods, they employ bespoke organic catalysts that are not commercially available.^14^ Separation of the organo-catalysts, or catalyst-derived side products, from the final products can also be challenging. Both of these factors limit the attractiveness of the methods for discovery applications and diminish the sustainability profile of the reactions. There is clearly space for a sustainable, operationally simple, and broadly applicable method employing commercially available bench-stable reagents to access alkyl sulfinamides. In this study, we disclose an iron-catalyzed decarboxylative sulfinylation of carboxylic acids using commercial bench-stable NSO reagents.^15^ The methodology uses inexpensive, earth-abundant iron salts under visible-light irradiation to generate alkyl radicals via ligand-to-metal charge transfer (LMCT), forging C–S bonds in a straightforward, scalable, and environmentally benign process. The method can also be translated to continuous-flow synthesis.

Iron-catalyzed decarboxylation, which operates by ligand-to-metal charge transfer (LMCT) under visible light irradiation,^16^ provides an attractive route to alkyl radicals and has been used to develop reactions such as Giese-type C–C bond formation,^17^ heteroarylations,^18^ borylation,^19^ azidation,^20^ and (fluoro)­methylations,^21^ among others.^22^ Inspired by these precedents, we were drawn to develop an iron-catalyzed synthesis of alkyl sulfinamides (Scheme 1c). Such a process would remove the limitations resulting from the use of noncommercial organo-catalysts and would provide a more sustainable and scalable route to sulfinamides.^23^

We selected hydrocinnamic acid and commercially available N-trityl sulfinylamine (TrNSO) as model substrates. DMSO was chosen as the reaction solvent due to its green credentials^24^ and compatibility with the high-throughput experimentation (HTE) platforms.^25^ Selected reaction optimizations are presented in Scheme 2. We found Fe­(OTf)_3_ to be an effective catalyst as it outperformed other Fe­(III) salts and provided the targeted sulfinamide in 85% yield using our standard conditions (entry 1, and Supporting Information). Key observations included triethylamine being base of choice and a loading of 20 mol % being optimal (entries 2–5). Reactions performed in the absence of any base were also less efficient (entry 6). Variation of light wavelength revealed broad tolerance (390–420 nm), with 390 nm irradiation providing an 80% yield and 420 nm giving 82%, which is consistent with LMCT absorption profiles (entries 7 and 8).^16^ Control experiments confirmed the requirements of both an iron catalyst and light (entries 9 and 10). Automated HTE runs confirmed robustness (see Supporting Information), achieving 66% yield using a DMSO/DMA solvent-mixture. The reaction conditions following the optimization process were Fe­(OTf)_3_ (15 mol %) with NEt_3_ (20 mol %), using 405 nm LEDs in DMSO (0.1 M).

**Scheme 2:**
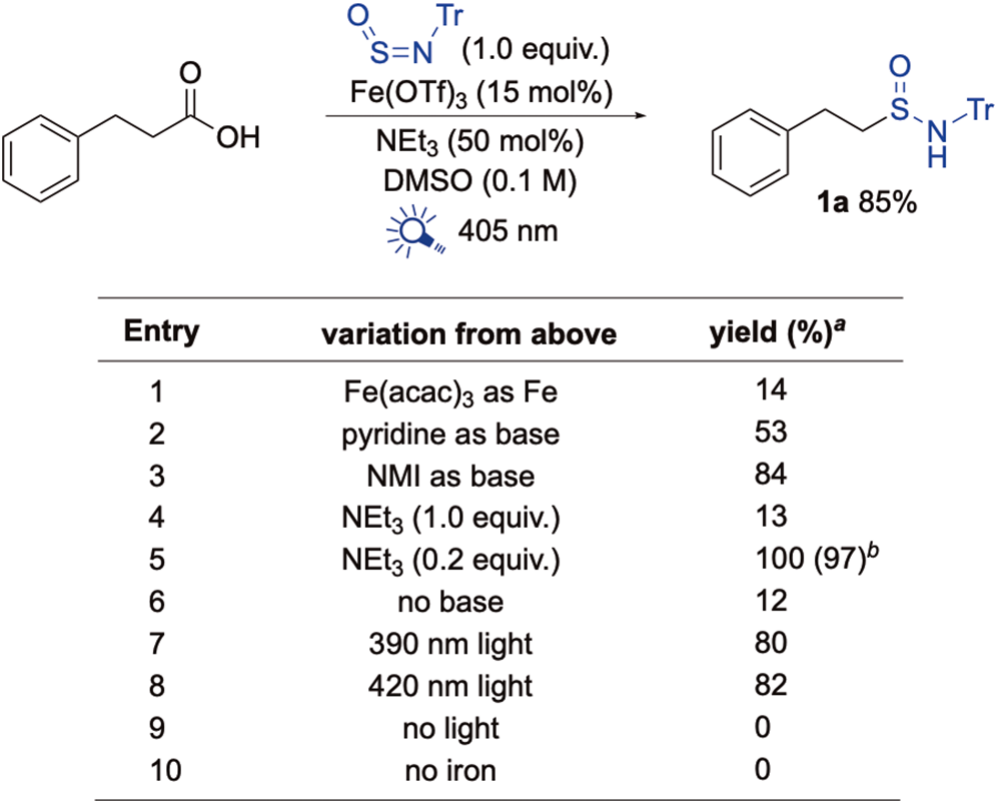
Selected Optimization Studies

a Yields of **1a** calculated from HPLC analysis against internal consumption of TrNSO.
b Isolated yield.

With an optimized system for the synthesis of *N*-trityl sulfinamide **1a** established, we next investigated the scope of the reaction with respect to the carboxylic acid component (Scheme 2). Encouragingly, the scope broadly mirrored the previously reported acridine-based chemistry,^13b^ with only minor deviations. A wide range of alkyl carboxylic acids was well-tolerated, including primary (**1a**–**k**), secondary (**1l**–**q**), and tertiary (**1r**–**t**) examples. Benzylic acids were also compatible (**1u**), although they delivered products in only moderate yields, likely due to the rapid dimerization of the benzylic radicals. Diverse functional groups were accommodated, including amides (**1d**), sulfonamides (**1p**), carbamates (**1f**–**i**, **v**, and **w**), and free alcohols (**1x**). Substrates featuring functionalized aromatic rings (**1d**, **s**, and **q**) were suitable, as were substrates that incorporated the heteroaromatics quinoline (**1u**), oxazole (**1k**), and benzothiazole (**1j**). Additionally, several more complex carboxylic acids were successfully transformed, including a bicyclopropane derivative (**1w**), a spirocycle (**1v**), β-amino acid valine (**1i**), and the steroid natural product chenodeoxycholic acid (**1x**), all of which provided the desired sulfinamides in good yields. The methodology proved effective on a preparative scale; using a continuous flow reactor, we achieved gram-scale reactions, furnishing sulfinamide **1p** in 82% yield.

We next evaluated alternative sulfinylamine reagents with a focus on bench-stable examples. Both N-*t*-octyl and N–O-*t*-butyl sulfinylamines provided the corresponding sulfinamides (**2a**, **b**) in good yields when using the optimized reaction conditions (Scheme 3b). However, TIPS-NSO was incompatible with these conditions, and the formation of TIPS-NH_2_ was observed. An evaluation of alternative iron salt catalysts revealed Fe­(NO_3_)_3_·9H_2_O as an effective catalyst, with the reaction between TIPS-NSO and hydrocinnamic acid now affording the desired silyl-protected sulfinamide (**2c**) in an excellent yield. Fe­(NO_3_)_3_·9H_2_O has the additional advantage that it is less costly and more widely available than Fe­(OTf)_3_.^26^ The ferric nitrate conditions were applied to a small selection of carboxylic acid substrates, with primary, secondary, and tertiary acids, used in combination with TIPS-NSO, all proving compatible, and the sulfinamide products were obtained in good yields (**3a**–**3h**, Scheme 3c). Extension to more complex substrates, including aspartic (**3i**) and glutamic (**3j**) acid derivatives, as well as a bicyclopropane (**3k**) example, was also successful. It is notable that the aspartic acid substrate was not successful in our original organo-catalyzed process.^13b^ These modified conditions were also compatible with the Tr-NSO reagent (examples **1a**, **1e**, and **1n**).

**Scheme 3:**
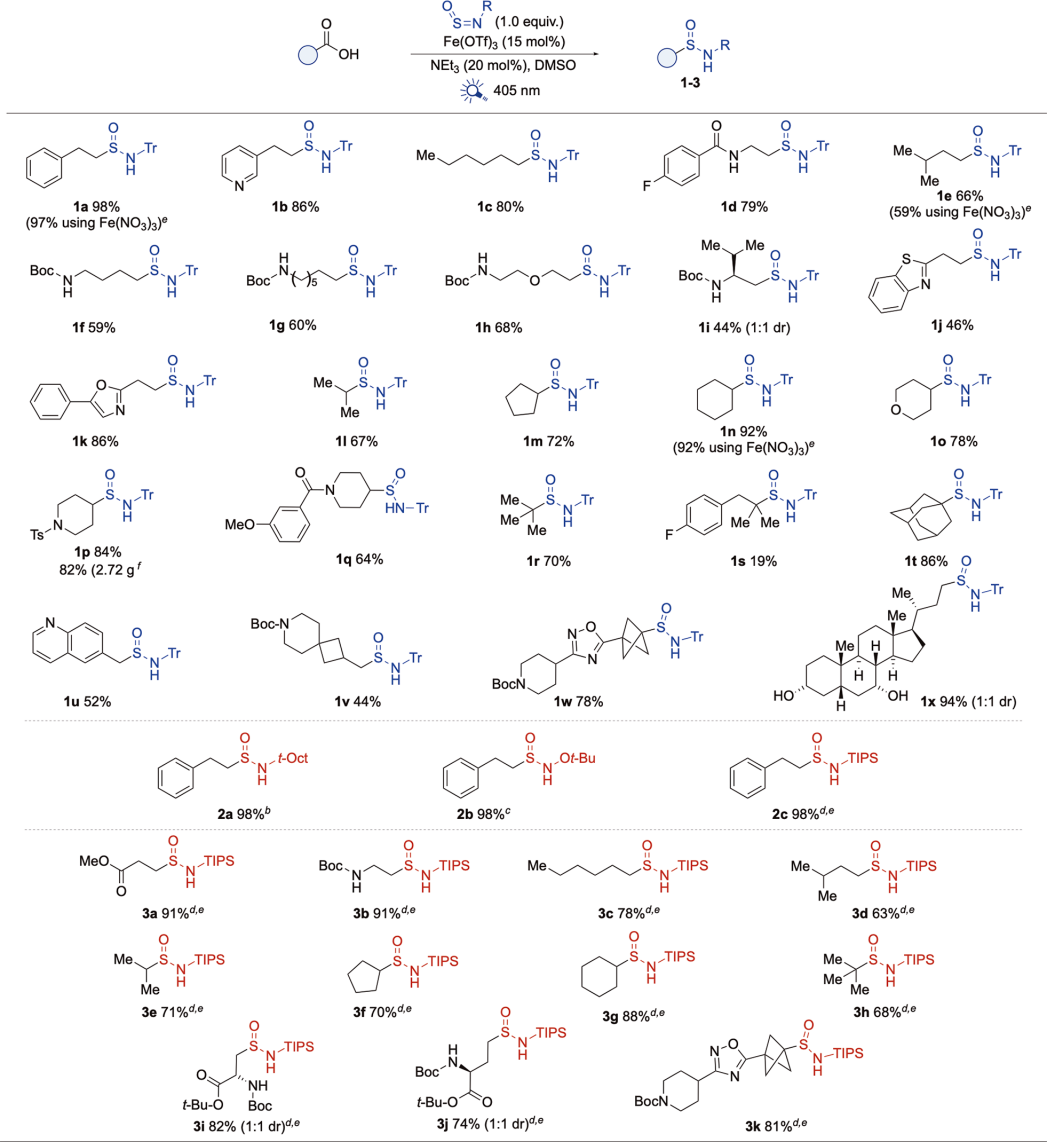
Scope of the Fe-Catalyzed Decarboxylative Synthesis of Alkyl Sulfinamides. (a) Acid Variation. (b) Sulfinylamine Variation. (c) Fe­(NO_3_)_3_ Evaluation^a^

a Reaction conditions: (i) carboxylic acid (0.2 mmol), Tr-NSO (0.2 mmol), Fe­(OTf)_3_ (15 mol %), NEt_3_ (20 mol %), 405 nm LEDs, DMSO (2 mL), rt, 3–18 h. Isolated yields. Compounds purified by reverse-phase semipreparative HPLC.
b Using *t*-Oct-NSO in place of Tr-NSO.
c Using *t*-BuO-NSO in place of Tr-NSO.
d Using TIPS-NSO in place of Tr-NSO.
e Fe­(NO_3_)_3_·9H_2_O used in place of Fe­(OTf)_3_. Acid (0.3 mmol) TIPS-NSO (0.2 mmol), Fe­(NO_3_)_3_·9H_2_O (15 mol %), NEt_3_ (20 mol %), 405 nm LEDs, DMSO (2 mL), rt, 3–18 h.
f Continuous flow reaction using a Lumidox II reactor.

One potential application of the developed chemistry is to the late-stage functionalization of complex PROTAC linkers. As a demonstration of scalability and operational simplicity, the bicyclo[1.1.1]­pentane (BCP)-containing PROTAC precursor **4** was subjected to Fe-catalyzed decarboxylative sulfinylation with TrNSO under the optimized flow conditions (Scheme 4, and Supporting Information). Performing the reaction in a 2 mL Lumidox II Flow reactor irradiated with 405 nm LEDs at 40 °C enabled continuous processing over several hours, delivering 1.27 g of the sulfinamide product **1w** in 74% yield. This sulfinamide intermediate was readily derivatized in three directions; oxidation with trichloroisocyanuric acid (TCCA) and silver fluoride afforded sulfonimidoyl fluoride **5**, treatment with TCCA followed by imidazole provided sulfonimidoyl imidazole **6**, while addition of water following the TCCA step provided sulfonamide **7**. Both of the activated sulfonimidamide species (**5** and **6**) retain the Boc-protected piperidine, enabling further conjugation steps essential for PROTAC synthesis. This sequence underscores the potential of iron-catalyzed decarboxylative sulfinylation as a practical and scalable route for accessing advanced PROTAC intermediates.

**Scheme 4:**
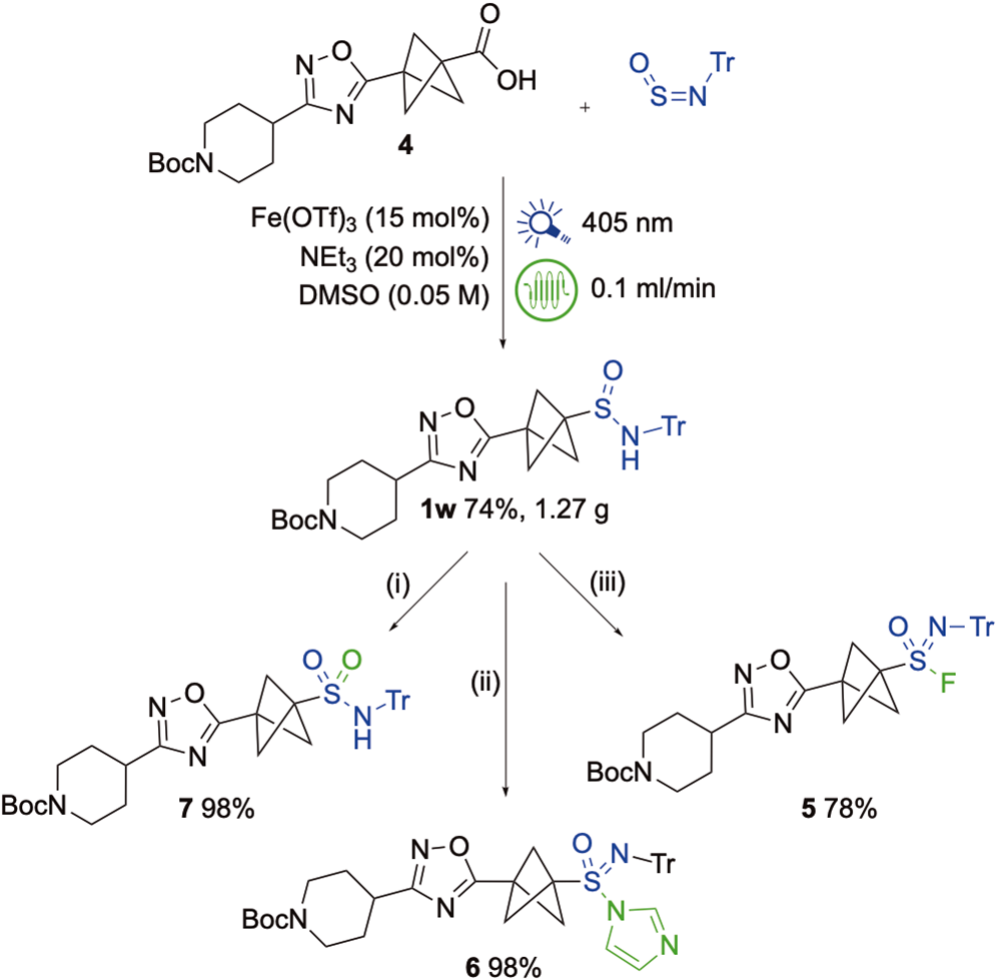
Flow Synthesis of Sulfinamide **1w** and Its Derivatisation to Sulfonimidoyl Fluoride **5**, Sulfonimidoyl Imidazole **6**, and Sulfonamide **7** ^a^

a Reaction conditions: Acid **4** (1.0 equiv), TrNSO (1.2 equiv), Fe­(OTf)_3_ (15 mol %), NEt_3_ (20 mol %), DMSO (0.05 M), 10 mL PFA coil reactor irradiated with 405 nm LEDs at 40 °C, 0.1 mL/min, t_res_ 20 min. Isolated yields. (i) TCCA, rt, then H_2_O, rt. (ii) TCCA, rt, then imidazole, rt. (iii) TCCA, rt, then AgF, rt, dark.

In conclusion, we have shown that iron-catalyzed, visible-light-mediated decarboxylative radical additions into commercially available and bench-stable NSO reagents provides efficient access to a broad range of alkyl sulfinamides. A key advance of this work is the ability to generalize the methodology beyond trityl-protected NSO reagents to include *t*-octyl, *t*-Bu-O, TIPS-derived sulfinylamine reagents, thus expanding the scope and utility of the platform. The optimized conditions are operationally simple, scalable in continuous flow, and compatible with substrates bearing diverse functional groups and complex, biologically relevant motifs. This chemistry offers a practical and sustainable route to sulfinamide intermediates for further derivatization into sulfonamides, sulfonimidamides, and related sulfur­(VI) compounds, enabling applications in modern drug discovery and beyond.

## Supplementary Material



## Data Availability

The data underlying this study are available in the published article and its Supporting Information.

## Notes

Experimental procedures and supporting characterization data and spectra (PDF)
