# Supplementary material for: Iron-Catalyzed Decarboxylative Sulfinylation of Alkyl Carboxylic Acids
Source: Org Lett. 2025 Sep 4;27(37):10325–9. doi: 10.1021/acs.orglett.5c03100 (PMC12455644; doi:10.1021/acs.orglett.5c03100)

Supporting Information

Iron-Catalyzed Decarboxylative Sulfinylation of Alkyl Carboxylic Acids

Matthew Southern,[a,b] Dr. Christopher Pearce,[b] and Prof. Michael C. Willis[a,*]

[*] michael.willis@chem.ox.ac.uk

[a] Department of Chemistry, University of Oxford, Chemistry Research Laboratory, Mansfield Road, Oxford, OX1 3TA, UK.

[b] Sygnature Discovery, Bio City, Pennyfoot St, Nottingham NG1 1GR, UK.

Table of Contents

1.General information 1
2.Optimisation of conditions 4
3. Experimental procedures 8
4. References 33
5. NMR Spectra 34

1. General information

1.1 General Considerations

All moisture-sensitive reactions were conducted under a nitrogen atmosphere using oven-dried glassware. Anhydrous solvents were obtained in the highest available purity from Acros Organics. Non-commercial compounds were sourced via Amici and used without further purification. Flash chromatography was performed on a Teledyne ISCO CombiFlash system using pre-packed silica cartridges (4–330 g) as specified. Reverse-phase chromatography was carried out on an automated mass-directed purification system using C18-derivatised silica (100 Å, 20–40 µm), monitored at 254 and 280 nm. ^{1}H NMR spectra were recorded on a Bruker Avance 400 MHz or Bruker 500MHz Avance III HD spectrometer equipped with a Bruker 5mm SmartProbeTM. Spectra were measured at 298 K using unless indicated otherwise. $CDCl_3$ was used as the solvent unless otherwise noted. Chemical shifts (δ) are reported in ppm, with multiplicities, coupling constants (J in Hz), and integration. Residual solvent signals ($CDCl_3$: δH = 7.26; DMSO-d_6: δH = 2.50; MeOH-d_4: δH = 3.34) served as internal references. ^{13}C NMR spectra were recorded on the same instrument and referenced to residual solvent signals ($CDCl_3$: δC = 77.0; DMSO-d_6: δC = 39.5; MeOH-d_4: δC = 49.9). Multiplicities are reported as follows: s (singlet), d (doublet), t (triplet), q (quartet), m (multiplet), br. (broad signal), app. (apparent). Low-resolution LCMS analyses were performed on a Waters Acquity i-Class UPLC with BEH C18 column (30 mm × 2.1 mm, 1.7 µm), operating at 50 °C with a linear gradient of 5–100% MeCN in 0.1% NH_4OH over 3 min at 0.8 mL/min. UPLC yields were determined by comparison to the *N*-sulfinyltritylamine (TrNSO) internal standard (λ = 254 nm). Method validation was confirmed by product isolation, matching within ± 2%. Isolated yields are reported relative to the lmiting reagent. High-resolution mass spectra

(HRMS) were recorded using either a Bruker microTOF (ESI), Waters LCT Premier (CI), or Waters BioAccord (ESI) instrument. Samples were submitted in MeOH (1 mg/mL) or neat (for CI). All accurate masses are reported within ± 5 ppm of the calculated value. Infrared spectra were recorded on a Bruker Tensor 27 Fourier Transform spectrometer over the range 600–4000 cm^{-1}. All absorption maxima ($\tilde{\nu}max$) are reported in wavenumbers (cm^{-1}). Melting points were recorded in degrees Celsius (°C) using a STUART scientific hot-stage microscope apparatus SMP1 or a Reichert melting point apparatus and are reported uncorrected. Heating was performed using standard laboratory equipment (heating mantle), with temperatures monitored externally by thermocouple. Reflux reactions were conducted using a condenser. Cooling to 0 °C was achieved with an ice-water bath. “Room temperature” refers to an ambient temperature of 21 ± 2 °C.

1.2 Instrumentation and Reaction Setups

Photochemical Reaction Optimisation

Optimisation reactions were performed in a Lumidox II® photoreactor (HepatoChem) with 405 nm diffuse LED irradiation. Reactions were run at power level 1 with active external fan cooling to maintain a temperature of 20 °C, verified via a thermocouple in a dummy vial. The reactor has an internal diameter of 54 mm and height of 69 mm. Vials (4 mL) were arranged symmetrically in the reactor carousel and stirred magnetically. All successful reactions were validated using an hepatochem reactor and 405 nm EvoluChem lamps.

Flow Reaction Setup

Flow experiments were carried out using a ThalesNano H-Cube Pro pump connected to a Lumidox II® photoreactor fitted with 405 nm diffuse LEDs (Analytical Sales and Services, New Jersey). The reactor coil consisted of PFA tubing (1/16" OD, 1.0 mm ID) with an illuminated volume of 2 mL. Residence times were achieved by adjusting flow rate. Reaction temperatures were regulated with an external chiller Humber Minichiller 300 OLE.

Preparative Purification

Preparative-scale purification was performed using a Waters Prep LC Classic system. Chromatographic separations employed Waters XSelect CSH C18 OBD Prep columns

(5 µm, 19 × 150 mm) and Waters XBridge BEH C18 OBD Prep columns (5 µm, 19 × 150 mm), following protocols established by Sygnature Discovery’s HTE group. Gradients of 0.1% ammonium hydroxide in H_2O/MeCN were used with diode-array and QDa detection. Fractions were pooled based on UV and MS purity, concentrated in vacuo, and analysed by NMR and HRMS.

2. Optimisation of conditions

Table S1: Metal catalysts screening

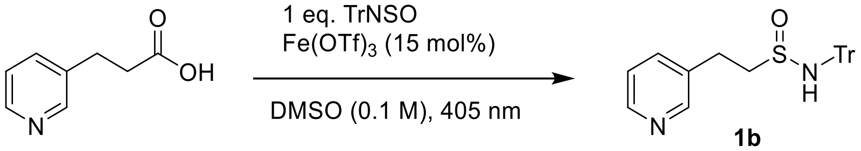

1b

**Entry**	**Metal source**	**Yield of 1b (%)**
**1**	$Fe(acac)_3$	14 (13)[a]
**2**	$FeCl_3$	13
**3**	$FeCl_3.6H_2O$	12
**4**	$Fe(NO_3)_3.9H_2O$	40
**5**	$InBr_3$	0
**6**	$FeBr_3$	6
**7**	$Fe(OTs)_3$	54 (52)[a]
**8**	**$Fe(OTf)_3$**	**56 (55)**[a]
**9**	$RuCl_3.6H_2O$	0
**10**	4-czipn	0
**11**	$Ir(dF(CF_3)ppy)_2(dtbbpy)PF_6$	1
**12**	Blank	0

Reaction conditions: 3-(pyridin-3-yl)propanoic acid (0.2 mmol), TrNSO (0.2 mmol), metal catalyst (15 mol%), DMSO (1 mL), rt, **2 hr**. Yields calculated by UPLC analysis using TrNSO as an internal standard. [a]Isolated yield

Table S2: Concentration screening

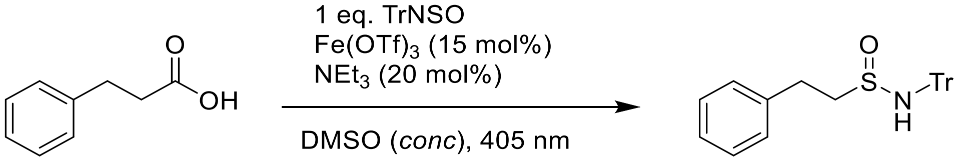

1a

**Entry**	**Concentration**	**Yield of 1a (%)**

**1**	0.05	87
**2**	**0.1**	**96**
**3**	0.2	72
**4**	0.5	0

Reaction conditions: Hydrocinnamic acid (0.05 mmol), TrNSO (0.05 mmol), $Fe(OTf)_3$ (15 mol%), NEt_3 (20 mol%), DMSO (0.1-1 mL), rt, 2 hr. Yields calculated by UPLC analysis using TrNSO as an internal standard.

Table S3: Control reactions

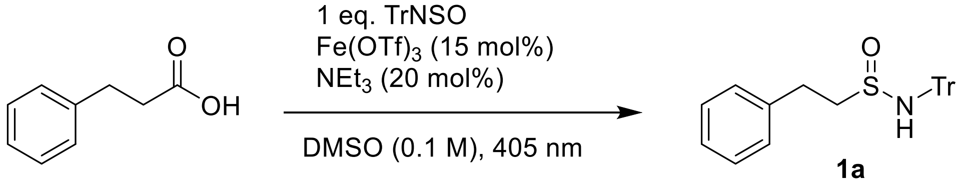

Entry	Deviation from standard conditions	Yield of 1a (%)
**1**	No light	0
**2**	No iron	0
**3**	390 nm	80
**4**	420 nm	82

Reaction conditions: Hydrocinnamic acid (0.2 mmol), TrNSO (0.2 mmol), $Fe(OTf)_3$ (15 mol%), NEt_3 (20 mol%), DMSO (2 mL), rt, 2 hr. Yields calculated by UPLC analysis using TrNSO as an internal standard.

Table S4: Base screening

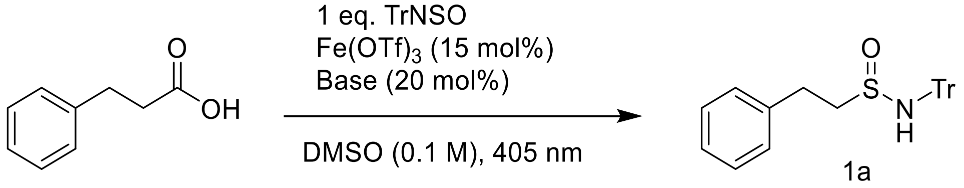

Entry	Base	Yield of 1a (%)
**1**	Pyridine	53

**2**	Cs_2CO_3	68
**3**	NMI	83
**4**	DABCO	85
**5**	DBU	80
**6**	**TEA**	**96**

Reaction conditions: Hydrocinnamic acid (0.2 mmol), TrNSO (0.2 mmol), $Fe(OTf)_3$ (15 mol%), DMSO (2 mL), rt, 2 hr. Yields calculated by UPLC analysis using TrNSO as an internal standard.

Table S5: Equivalents of base screening

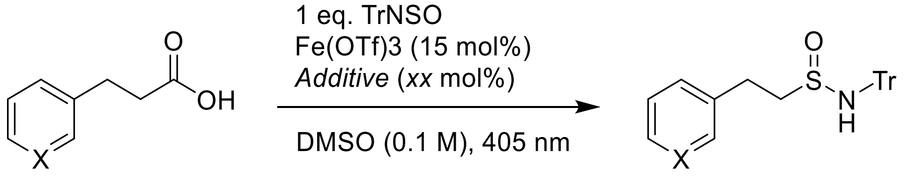

Entry	Additive (mol%)	Yield of 1b (X=N) (%)	Yield of 1a (X=CH) (%)
**1**	None	86	12
**2**	Pyridine (100 mol%)	7	0
**3**	Pyridine (20 mol %)	-	53
**4**	Triethylamine (100 mol %)	3	15
**5**	Triethylamine (50 mol %)	-	13
**6**	Triethylamine (20 mol %)	88	97 (96)
**7**	*N*-methylimidazole (20 mol%)	-	83
**8**	*N*-methylimidazole (50 mol%)	88	95

Reaction conditions: *Carboxylic acid* (0.2 mmol), TrNSO (0.2 mmol), $Fe(OTf)_3$ (15 mol%), DMSO (2 mL), rt, 2 hr. Yields calculated by UPLC analysis using TrNSO as an internal standard.

Table S6: Solvent screening

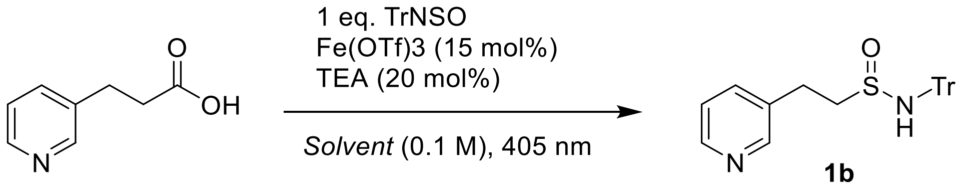

1b

Entry	Solvent	Yield of 1b (%)
**1**	DMSO	86
**2**	Water	1
**3**	DMF	0
**4**	EtOAc	0
**5**	NMP	0
**6**	DCM	4
**7**	Acetone	10
**8**	PhMe	0
**9**	MeOH	14
**10**	HFIPA	8
**11**	2-MeTHF	1
**12**	MeCN	1

Reaction conditions: Hydrocinnamic acid (0.2 mmol), TrNSO (0.2 mmol), $Fe(OTf)_3$ (15 mol%),, NEt_3 (20 mol%) *solvent* (2 mL) rt, 3 hr. Yields calculated by UPLC analysis using TrNSO as an internal standard.

Table S7: Optimisation of flow reaction

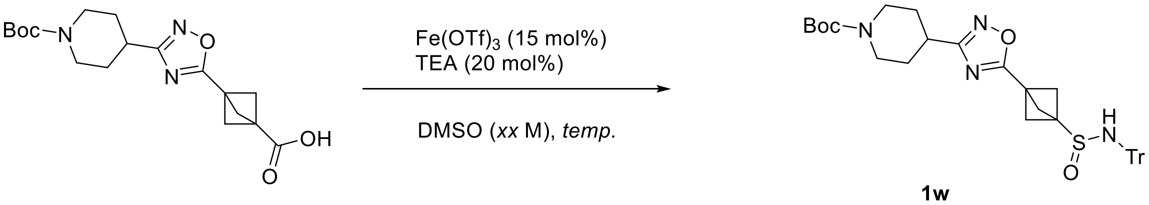

1w

Entry	Concentration /Molar	Temperature / °C	Residence time / min	Yield of × (%)
**1**	0.25	25	4	16
**2**	0.1	25	4	32

**3**	0.05	25	4	46
**4**	0.025	25	4	44
**5**	0.05	40	4	57
**6**	0.05	50	4	52
**7**	0.05	40	8	69
**8**	**0.05**	**40**	**20**	**78**

Reaction conditions: 3-(3-(1-(tert-butoxycarbonyl)piperidin-4-yl)-1,2,4-oxadiazol-5-yl)bicyclo[1.1.1]pentane-1-carboxylic acid (0.2 mmol), TrNSO (0.2 mmol), $Fe(OTf)_3$ (15 mol%),, NEt_3 (20 mol%) DMSO (2 mL), Lumidox II®, 405 nm, level 5/5. Yields are calculated by UPLC analysis using TrNSO as an internal standard.

3. Experimental procedures

General Procedure A - conversion of alkyl carboxylic acids into sulfinamides.

Carboxylic acid (0.2 mmol, 1.0 equiv.) and triethylamine (0.04 mmol, 20 mol%) were dissolved in dimethyl sulfoxide (2.0 mL, 0.1 M) in a 4 mL vial. $Fe(OTf)_3$ (0.03 mmol, 15 mol%) was then added, followed by the *N*-sulfinyl reagent (0.3 mmol, 1.5 equiv.). The headspace was flushed with nitrogen, and the vial sealed. The reaction was then stirred under 405 nm LED irradiation (see Figure S1 for experimental setup) at room temperature for 18 h. Upon completion (determined by UPLC or TLC), the reaction mixture was diluted with diethyl ether (20 mL), washed with saturated aqueous $NaHCO_3$ (3 × 10 mL for 0.2 mmol reaction) and brine (1 × 10 mL for 0.2 mmol reaction). The organic layer was dried over $MgSO_4$, filtered, concentrated *in vacuo*, and the crude product was purified by silica gel flash chromatography or reverse-phase semi-preparative HPLC.

General Procedure B - conversion of alkyl carboxylic acids into silyl-sulfinamides.

Carboxylic acid (0.3 mmol, 1.0 equiv.) and triethylamine (0.06 mmol, 20 mol%) were dissolved in dimethyl sulfoxide (2.0 mL, 0.1 M) in a 4 mL vial. $Fe(NO_3)_3{\cdot}9H_2O$ (0.045 mmol, 15 mol%) was added, the headspace was flushed with nitrogen, and the vial sealed. The reaction mixture was stirred for 15 minutes in the dark. The *N*-sulfinyl

reagent (0.2 mmol, 0.7 equiv.) was added and the reaction was then stirred under 405 nm LED irradiation (see Figure S1 for experimental setup) at room temperature for 18 h. Upon completion (determined by UPLC or TLC), the reaction mixture was diluted with diethyl ether (20 mL), washed with saturated aqueous $NaHCO_3$ (3 × 10 mL for 0.2 mmol reaction) and brine (1 × 10 mL for 0.2 mmol reaction). The organic layer was dried over $MgSO_4$, filtered, concentrated *in vacuo*, and the crude product was purified by silica gel flash chromatography.

2-phenyl-*N*-tritylethane-1-sulfinamide 1a

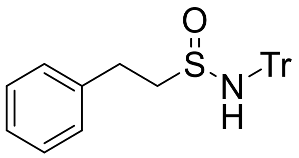

Normal phase work-up:

The title compound was prepared according to **General Procedure A** using hydrocinnamic acid (0.2 mmol, 30 mg) and *N*-sulfinyltritylamine (0.3 mmol, 92 mg), $Fe(OTf)_3$ (0.03 mmol, 15 mg) and triethylamine (0.04 mmol, 5.4 μL) in DMSO (2 mL). Purification by silica gel flash column chromatography (10-50% EtOAc in isohexane) afforded the *title compound* as a white solid (80 mg, 98%). The spectral data is consistent with the literature.[1]

^{1}H NMR (500 MHz, $CDCl_3$) δ 7.33–7.15 (m, 20H), 4.96 (s, 1H), 3.08–2.89 (m, 4H); **^{13}C NMR** (126 MHz, $CDCl_3$) δ 144.9, 139.2, 129.3, 128.9, 128.7, 128.2, 127.5, 101.8, 73.0, 58.4, 29.3; **IR** (ATR): ṽ (cm^{-1}) = 3095, 2990, 2950, 1699, 1576, 1449; **LRMS** (ESI$^+$, *m/z*) 434.2 $[M+Na]^+$.

The title compound was also prepared according to **General Procedure B**, using hydrocinnamic acid (0.2 mmol, 30 mg) and *N*-sulfinyltritylamine (0.3 mmol, 92 mg), $Fe(NO_3)_3 \cdot 9H_2O$ (0.045 mmol, 18 mg) and triethylamine (0.06 mmol, 8.4 μL) in DMSO (2 mL). The title compound was isolated as a white solid (77 mg, 97%).

2-(pyridin-3-yl)-*N*-tritylethane-1-sulfinamide 1b

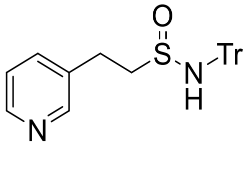

The title compound was prepared according to **General Procedure A** using 3-(pyridin-3-yl)propanoic acid (0.2 mmol, 30 mg) and *N*-sulfinyltritylamine (0.3 mmol, 92 mg), $Fe(OTf)_3$ (0.03 mmol, 15 mg) and triethylamine (0.04 mmol, 5.4 µL) in DMSO (2 mL). Purification by silica gel flash column chromatography (10-100% EtOAc in isohexane) afforded the *title compound* as a white solid (71 mg, 86%). The spectral data is consistent with the literature.[1]

^{1}H NMR (500 MHz, $CDCl_3$) δ 8.51–8.40 (m, 2H), 7.56 (dt, J = 7.9, 2.0 Hz, 1H), 7.36–7.19 (m, 16H), 5.13 (s, 1H), 3.06–2.92 (m, 4H); **^{13}C NMR** (126 MHz, $CDCl_3$) δ 149.5, 147.7, 144.8, 136.8, 134.9, 129.2, 128.3, 127.6, 123.8, 73.1, 57.9, 27.1; **IR** (ATR): ṽ (cm^{-1}) = 3403, 3181, 3055, 1596, 1491, 1442; **LRMS** (ESI^+, *m/z*) 413.1 $[M+H]^+$.

***N*-Tritylhexane-1-sulfinamide 1c**

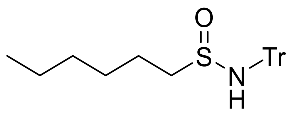

The title compound was prepared according to **General Procedure A** using heptanoic acid (0.2 mmol, 26 mg) and *N*-sulfinyltritylamine (0.3 mmol, 92 mg), $Fe(OTf)_3$ (0.03 mmol, 15 mg) and triethylamine (0.04 mmol, 5.4 µL) in DMSO (2.0 mL). Purification by silica gel flash column chromatography (15–30% EtOAc in Petrol) afforded the title compound as a colourless oil (63 mg, 80 %).

^{1}H NMR (400 MHz, $CDCl_3$) δ 7.31 – 7.15 (m, 15H), 4.76 (s, 1H), 2.63 (ddt, J = 9.7, 5.9, 2.8 Hz, 2H), 1.61 – 1.52 (m, 2H), 1.31 – 1.11 (m, 6H), 0.86 – 0.74 (m, 3H); **^{13}C NMR** (101 MHz, $CDCl_3$) δ 144.9, 129.2, 128.0, 127.4, 72.8, 57.8, 31.4, 28.3, 23.2, 22.4, 14.0; **IR** (ATR): ṽ (cm^{-1}) = 3193, 3062, 2956, 2927, 2858, 2597, 2494; **LRMS** (ESI^+, m/z) 392.3 $[M+H]^+$; **HRMS** (ESI^+, m/z): Calc. for $C_{25}H_{30}NOS$ [M+H] = 392.2043, Found = 392.2055.

4-fluoro-*N*-(2-((tritylamino)sulfinyl)ethyl)benzamide 1d

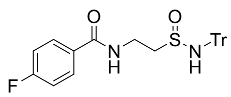

The title compound was prepared according to **General Procedure A** using 3-(4-fluorobenzamido)propanoic acid (0.2 mmol, 42 mg) and *N*-sulfinyltritylamine (0.3 mmol, 92 mg), $Fe(OTf)_3$ (0.03 mmol, 15 mg) and triethylamine (0.04 mmol, 5.4 µL) in

DMSO (2 mL). Purification by silica gel flash column chromatography (10-100% EtOAc in isohexane) afforded the *title compound* as a white solid (75 mg, 79%).

^{1}H NMR (500 MHz, DMSO) δ 8.61 (t, J = 5.3 Hz, 1H), 7.89–7.82 (m, 2H), 7.33–7.19 (m, 18H), 3.61–3.51 (m, 1H), 3.46–3.37 (m, 1H), 3.19 (app. t, J = 6.6 Hz, 2H); **^{13}C NMR** (126 MHz, DMSO) δ 165.2, 163.9 (d, J = 248.5 Hz), 145.1, 130.7 (d, J = 3.0 Hz), 129.8 (d, J = 9.1 Hz), 129.3, 127.5, 127.0, 115.2 (d, J = 21.6 Hz), 72.5, 54.4, 35.0; **19F NMR** (471 MHz, DMSO) δ -109.42 (tt, J = 8.9, 5.4 Hz); **Melting point** (CH_2Cl_2) = 110 – 113 °C; **IR** (ATR): ṽ (cm^{-1}) = 3238, 3055, 1636, 1562, 1504, 1445; **LRMS** (ESI$^+$, *m/z*) 495.3 [M+Na]$^+$; **HRMS** (ESI$^+$, m/z): Calc. for $C_{28}H_{25}FN_2O_2SNa$ [M+Na] = 495.1513, Found = 495.1508

3-Methyl-*N*-tritylbutane-1-sulfinamide 1e

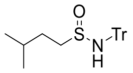

The title compound was prepared according to **General Procedure A** using 3-methylbutanoic acid (0.2 mmol, 23 mg) and *N*-sulfinyltritylamine (0.3 mmol, 92 mg), $Fe(OTf)_3$ (0.03 mmol, 15 mg) and triethylamine (0.04 mmol, 5.4 µL) in DMSO (2.0 mL). Purification by silica gel flash column chromatography (15–30% EtOAc in Petrol) afforded the title compound as a colourless oil (50 mg, 66%).

^{1}H NMR (400 MHz, $CDCl_3$) δ 7.31 – 7.16 (m, 15H), 4.73 (s, 1H), 2.61 (t, J = 7.2 Hz, 2H), 1.53 (td, J = 7.2, 6.5 Hz, 1H), 1.49 – 1.40 (m, 2H), 0.80 (d, J = 6.5 Hz, 6H); **^{13}C NMR** (101 MHz, $CDCl_3$) δ 144.9, 129.2, 128.0, 127.4, 72.8, 55.8, 31.8, 27.4, 22.4; **IR (ATR):** ṽ (cm^{-1}) = 3120, 2958, 1502, 1488; **LRMS** (ESI$^+$, m/z) 378.2 [M+H]$^+$; **HRMS** (ESI$^+$, m/z): Calc. for $C_{24}H_{27}NOSH$ [M+H]$^+$ = 378.1886, Found = 378.1899.

The title compound was also prepared according to **General Procedure B**, using 3-methylbutanoic acid (0.2 mmol, 23 mg) and *N*-sulfinyltritylamine (0.3 mmol, 92 mg), $Fe(NO_3)_3 \cdot 9H_2O$ (0.045 mmol, 18 mg) and triethylamine (0.06 mmol, 8.4 µL) in DMSO (2 mL). The title compound was isolated as a white colourless oil (45 mg, 59%).

tert-Butyl (4-((tritylamino)sulfinyl)butyl)carbamate 1f

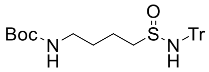

The title compound was prepared according to **General Procedure A** using 5-((tert-butoxycarbonyl)amino)pentanoic acid (0.2 mmol, 43 mg) and *N*-sulfinyltritylamine (0.3 mmol, 92 mg), $Fe(OTf)_3$ (0.03 mmol, 15 mg) and triethylamine (0.04 mmol, 5.4 µL) in DMSO (2.0 mL). Purification by silica gel flash column chromatography (15 – 100% EtOAc in Petrol) afforded the title compound as a yellow gum (56 mg, 59%).

^{1}H NMR (400 MHz, $CDCl_3$) δ 7.41 – 7.24 (m, 15H), 5.08 (s, 1H), 4.63 (s, 1H), 3.15 – 3.05 (m, 2H), 2.76 (t, J = 7.5 Hz, 2H), 1.74 – 1.62 (m, 2H), 1.60 – 1.50 (m, 2H), 1.44 (s, 9H); **^{13}C NMR** (101 MHz, $CDCl_3$) δ 156.0, 144.9, 129.2, 128.0, 127.4, 79.2, 72.9, 56.9, 39.9, 29.0, 28.4, 20.7; **IR** (ATR): ṽ (cm^{-1}) = 3218, 2061, 2919, 1688, 1494; **LRMS** (ESI^+, m/z) 479.1 $[M+H]^+$; **HRMS** (ESI^+, m/z): Calc. for $C_{28}H_{35}N_2O_3S$ $[M+H]^+$ = 479.2363, Found = 479.2373.

tert-Butyl (7-((tritylamino)sulfinyl)heptyl)carbamate 1g

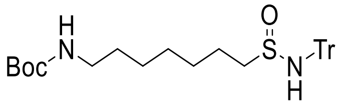

The title compound was prepared according to **General Procedure A** using 8-((tert-butoxycarbonyl)amino)octanoic acid (0.2 mmol, 52 mg) and *N*-sulfinyltritylamine (0.3 mmol, 92 mg), $Fe(OTf)_3$ (0.03 mmol, 15 mg) and triethylamine (0.04 mmol, 5.4 µL) in DMSO (2.0 mL). Purification by silica gel flash column chromatography (15 – 100% EtOAc in Petrol) afforded the title compound as a white gum (62 mg, 60%).

^{1}H NMR (400 MHz, $CDCl_3$) δ 7.36 – 7.22 (m, 15H), 4.98 – 4.94 (m, 1H), 4.53 (s, 1H), 3.08 (m, 2H), 2.71 (m, 2H), 1.63 (m, 2H), 1.43 (s, 11H), 1.37 – 1.23 (m, 6H); **^{13}C NMR** (101 MHz, $CDCl_3$) δ 156.0, 144.9, 129.2, 128.0, 127.3, 79.0, 72.9, 57.5, 40.5, 29.9, 28.9, 28.5, 28.5, 26.5, 23.3; **IR** (ATR): ṽ (cm^{-1}) = 3062, 2979, 2856, 1692, 1496; **LRMS** (ESI^+, m/z) 521.0 $[M+H]^+$; **HRMS** (ESI^+, m/z): Calc. for $C_{31}H_{41}N_2O_3S$ $[M+H]^+$ = 521.2832, Found = 521.2848.

tert-Butyl (2-(2-((tritylamino)sulfinyl)ethoxy)ethyl)carbamate 1h

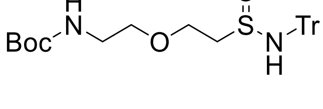

The title compound was prepared according to **General Procedure A** using 3-(2-((tert-butoxycarbonyl)amino)ethoxy)propanoic acid (0.2 mmol, 47 mg) and *N*-sulfinyltritylamine (0.3 mmol, 92 mg), $Fe(OTf)_3$ (0.03 mmol, 15 mg) and triethylamine (0.04 mmol, 5.4 μL) in DMSO (2.0 mL). Purification by silica gel flash column chromatography (15 – 100% EtOAc in Petrol) afforded the title compound as a white gum (67 mg, 68%).

^{1}H NMR (400 MHz, $CDCl_3$) δ 7.32 – 7.13 (m, 15H), 6.36 (s, 1H), 4.82 (s, 1H), 4.30 (ddd, J = 10.4, 8.0, 4.3 Hz, 1H), 3.64 (dt, J = 10.4, 4.3 Hz, 1H), 3.47 (t, J = 4.4 Hz, 2H), 3.21 – 3.14 (m, 2H), 2.70 (t, J = 4.4 Hz, 2H), 1.34 (s, 9H); **^{13}C NMR** (101 MHz, $CDCl_3$) δ 144.9, 129.1, 128.5, 128.4, 128.1, 127.3, 72.6, 70.5, 62.9, 53.3, 40.9, 28.4. **IR (ATR)**: ṽ (cm^{-1}) = 3446, 2979, 2914, 1705, 1496, 1447; **LRMS** (ESI^+, m/z) 517.1 $[M+H]^+$;**HRMS** (ESI^+, m/z): Calc. for $C_{28}H_{34}N_2O_4SNa$ $[M+Na]^+$ = 517.2132, Found = 517.2140

tert-butyl ((2S)-3-methyl-1-((tritylamino)sulfinyl)butan-2-yl)carbamate 1i

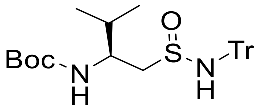

The title compound was prepared according to **General Procedure A** using (R)-3-((tert-butoxycarbonyl)amino)-4-methylpentanoic acid (0.2 mmol, 46 mg) and *N*-sulfinyltritylamine (0.3 mmol, 92 mg), $Fe(OTf)_3$ (0.03 mmol, 15 mg) and triethylamine (0.04 mmol, 5.4 μL) in DMSO (2 mL). Purification by prep-HPLC afforded the *title compound* as a white gum (43 mg, 44%, 1:1 inseparable mixture of diastereomers).

^{1}H NMR (500 MHz, $CDCl_3$) δ 7.38–7.21 (m, 15H), 6.00 (s, 0.5H), 5.37 (s, 0.5H), 5.25 (d, J = 9.3 Hz, 0.5H), 4.71 (d, J = 9.0 Hz, 0.5H), 3.95–3.86 (m, 0.5H), 3.70 (ddt, J = 9.3, 7.4, 3.7 Hz, 0.5H), 2.97 (dd, J = 13.3, 7.4 Hz, 1H), 2.89–2.80 (m, 1H), 1.90–1.79 (m, 1H), 1.39 (s, 4.5H), 1.35 (s, 4.5 H), 0.91–0.86 (m, 3H), 0.86–0.82 (m, 3H); **^{13}C NMR** (126 MHz, $CDCl_3$) δ 155.7, 145.1, 145.0, 129.4, 129.1, 128.2, 128.1, 127.46, 127.4, 80.1, 79.4, 73.2, 73.1, 59.1, 58.7, 52.3, 51.1, 32.0, 31.5, 28.5, 19.5, 19.1, 19.0, 17.3; **IR** (ATR): ṽ (cm^{-1}) =3169, 3061, 1590, 1567, 1493, 1402; **LRMS** (ESI^+, *m/z*) 392.2 [M-*t*Bu]$^+$; **HRMS** (ESI^+, m/z): Calc. for formula $C_{29}H_{37}N_2O_3S$ $[M+H]^+$ = 492.2447, Found = 492.2463

2-(benzo[d]thiazol-2-yl)-*N*-tritylethane-1-sulfinamide 1j

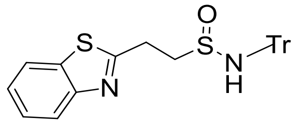

The title compound was prepared according to **General Procedure A** using 3-(benzo[d]thiazol-2-yl)propanoic acid (0.2 mmol, 42 mg) and *N*-sulfinyltritylamine (0.3 mmol, 92 mg), $Fe(OTf)_3$ (0.03 mmol, 15 mg) and triethylamine (0.04 mmol, 5.4 µL) in DMSO (2 mL). Purification by semi-preparative HPLC (10-40% MeCN) afforded the *title compound* as a colourless gum (43.0 mg, 46%).

^{1}H NMR (500 MHz, $CDCl_3$) δ 7.88–7.79 (m, 1H), 7.55–7.49 (m, 1H), 7.42–7.37 (m, 5H), 7.37–7.33 (m, 1H), 7.30–7.21 (m, 11H), 7.08 (s, 1H), 3.85 (dt, J = 20.9, 8.2 Hz, 1H), 3.35–3.25 (m, 2H), 3.05 (dt, J = 20.9, 10.2 Hz, 1H); **^{13}C NMR** (126 MHz, $CDCl_3$) δ 169.6, 152.7, 145.1, 135.3, 129.3, 128.1, 127.3, 126.2, 125.2, 122.6, 121.6, 72.9, 52.0, 25.5; **IR** (ATR): ṽ (cm^{-1}) =3161, 3055, 1593, 1519, 1493, 1445; **LRMS** (ESI^+, *m/z*) 469.2 $[M+H]^+$; **HRMS** (ESI^+, m/z): Calc. for formula $C_{28}H_{24}N_2OS_2Na$ $[M+Na]^+$ = 491.1222, Found = 491.1207

2-(5-phenyloxazol-2-yl)-*N*-tritylethane-1-sulfinamide 1k

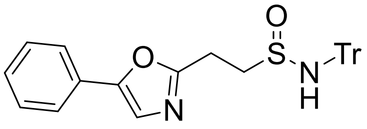

The title compound was prepared according to **General Procedure A** using 3-(5-phenyloxazol-2-yl)propanoic acid (0.2 mmol, 43 mg) and *N*-sulfinyltritylamine (0.3 mmol, 92 mg), $Fe(OTf)_3$ (0.03 mmol, 15 mg) and triethylamine (0.04 mmol, 5.4 µL) in DMSO (2 mL). Purification by silica gel flash column chromatography (10-80% EtOAc in isohexane) afforded the *title compound* as a white solid (82 mg, 86%).

^{1}H NMR (500 MHz, $CDCl_3$) δ 7.57 (d, J = 7.6 Hz, 2H), 7.41 (app. t, J = 7.6 Hz, 2H), 7.36 – 7.33 (m, 5H), 7.31 – 7.24 (m, 11H), 7.11 (br. s, 1H), 6.60 – 6.49 (br. s, 1H), 3.47 (dt, J = 17.0, 7.0 Hz, 1H), 3.29 (dt, J = 13.6, 6.8 Hz, 1H), 3.19 (dt, J = 13.6, 7.0 Hz, 1H), 3.10 (dt, J = 17.0, 6.8 Hz, 1H); **^{13}C NMR** (126 MHz, $CDCl_3$) δ 162.4 , 151.8, 145.0, 129.4, 129.1, 129.0, 128.6 , 128.1, 127.4, 124.3, 121.5, 73.2, 51.5, 21.1; **Melting point** (CH_2Cl_2) = 116 – 118 °C; **IR** (ATR): ṽ (cm^{-1}) =3198, 3055, 1590, 1559, 1491, 144;

LRMS (ESI$^+$, *m/z*) 479.2 [M+H]$^+$; **HRMS** (ESI$^+$, *m/z*) Calc. for formula $C_{30}H_{26}N_2O_2SNa$ [M+Na]$^+$ = 501.1607, Found = 501.1610.

***N*-Tritylpropane-2-sulfinamide 1l**

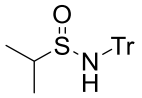

The title compound was prepared according to **General Procedure A** using isobutyric acid (0.2 mmol, 18 mg) and *N*-sulfinyltritylamine (0.3 mmol, 92 mg), $Fe(OTf)_3$ (0.03 mmol, 15 mg) and triethylamine (0.04 mmol, 5.4 µL) in DMSO (2.0 mL). Purification by silica gel flash column chromatography (15–30% EtOAc in Petrol) afforded the title compound as a colourless oil (47 mg, 67%).

^{1}H NMR (400 MHz, $CDCl_3$) δ 7.31 – 7.14 (m, 15H), 4.61 (s, 1H), 2.69 (p, J = 6.9 Hz, 1H), 1.19 (t, J = 6.9 Hz, 6H); **^{13}C NMR** (101 MHz, $CDCl_3$) δ 144.9, 129.4, 128.0, 127.4, 72.8, 55.4, 16.1, 14.9; **IR** (ATR): ṽ (cm^{-1}) = 3211, 3053, 2986, 1485, 1466 **LRMS** (ESI$^+$, m/z) 372.1 [M+Na]$^+$; **HRMS** (ESI$^+$, m/z): Calc. for $C_{22}H_{23}NOSNa$ [M+Na]$^+$ = 372.1393, Found = 372.1399.

***N*-tritylcyclopentanesulfinamide 1m**

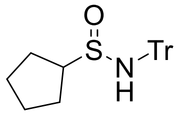

The title compound was prepared according to **General Procedure A** using cyclopentanecarboxylic acid (0.2 mmol, 23 mg) and *N*-sulfinyltritylamine (0.3 mmol, 92 mg), $Fe(OTf)_3$ (0.03 mmol, 15 mg) and triethylamine (0.04 mmol, 5.4 µL) in DMSO (2.0 mL). Purification by silica gel flash column chromatography (15–30% EtOAc in Petrol) afforded the title compound as a white solid (54 mg, 72%). The spectral data is consistent with the literature.[1]

^{1}H NMR (400 MHz, $CDCl_3$) δ 7.31 – 7.14 (m, 15H), 4.66 (s, 1H), 3.05 (tt, J = 8.7, 6.5 Hz, 1H), 2.07 – 1.93 (m, 1H), 1.92 – 1.75 (m, 2H), 1.72 – 1.46 (m, 5H); **^{13}C NMR** (101 MHz, $CDCl_3$) δ 144.9, 129.4, 127.9, 127.3, 72.8, 65.2, 28.3, 25.9, 25.6, 25.4; **IR** (ATR): ṽ (cm^{-1}) = 3186, 3061, 2910, 1598, 1494; **LRMS** (ESI$^+$, m/z) 376.2 [M+H]$^+$; **HRMS** (ESI$^+$, m/z): Calc. for $C_{24}H_{26}NOS$ [M+H]$^+$ = 376.1730, Found = 376.1737

***N*-Tritylcyclohexanesulfinamide 1n**

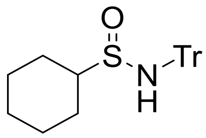

The title compound was prepared according to **General Procedure A** using cyclohexanecarboxylic acid (0.2 mmol, 26 mg) and *N*-sulfinyltritylamine (0.3 mmol, 92 mg), $Fe(OTf)_3$ (0.03 mmol, 15 mg) and triethylamine (0.04 mmol, 5.4 μL) in DMSO (2.0 mL). Purification by silica gel flash column chromatography (15–30% EtOAc in Petrol) afforded the title compound as a white solid (72 mg, 92%). The spectral data is consistent with the literature.[1]

^{1}H NMR (500 MHz, $CDCl_3$) δ 7.33 – 7.16 (m, 15H), 4.65 (s, 1H), 2.44 (tt, J = 11.3, 3.6 Hz, 1H), 1.96 (dd, J = 12.9, 3.7 Hz, 2H), 1.76 (ddt, J = 16.9, 12.6, 3.7 Hz, 2H), 1.64 – 1.54 (m, 1H), 1.43 – 1.09 (m, 5H); **^{13}C NMR** (101 MHz, $CDCl_3$) δ 145.0, 129.4, 128.0, 127.3, 72.9, 64.3, 26.8, 25.6, 25.6, 25.4, 25.3; **IR** (ATR): ṽ (cm^{-1}) = 3206, 3060, 2929, 2845, 1597, 1493; **LRMS** (ESI^+, m/z) 390.2 $[M+H]^+$; **HRMS** (ESI^+, m/z): Calc. for $C_{25}H_{28}NOS$ $[M+H]^+$ = 390.1886, Found = 390.1897.

The title compound was also prepared according to **General Procedure B**, using 3-methylbutanoic acid (0.2 mmol, 23 mg) and *N*-sulfinyltritylamine (0.3 mmol, 92 mg), $Fe(NO_3)_3·9H_2O$ (0.045 mmol, 18 mg) and triethylamine (0.06 mmol, 8.4 μL) in DMSO (2 mL). The title compound was isolated as a white solid (71 mg, 92%).

***N*-trityltetrahydro-2H-pyran-4-sulfinamide 1o**

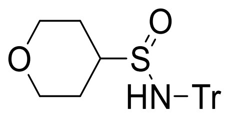

The title compound was prepared according to **General Procedure A** using tetrahydro-2H-pyran-4-carboxylic acid (0.2 mmol, 26 mg) and *N*-sulfinyltritylamine (0.3 mmol, 92 mg), $Fe(OTf)_3$ (0.03 mmol, 15 mg) and triethylamine (0.04 mmol, 5.4 μL) in DMSO (2 mL). Purification by semi-preparative HPLC (10-40% MeCN) afforded the *title compound* as a white solid (61 mg, 78%).

^{1}H NMR (500 MHz, $CDCl_3$) δ 7.39–7.10 (m, 15H), 5.03 (s, 1H), 4.07–3.97 (m, 2H), 3.39 (ddd, J = 11.5, 4.6, 2.4 Hz, 2H), 2.85 (tt, J = 11.7, 4.1 Hz, 1H), 1.95–1.86 (m, 2H), 1.68 (dddd, J = 25.0, 13.5, 11.7, 4.6 Hz, 2H); **^{13}C NMR** (126 MHz, $CDCl_3$) δ 144.8, 129.5, 128.1, 127.6, 73.2, 67.1, 67.0, 61.4, 26.8, 26.5; **Melting point** (CH_2Cl_2) = 104 – 107 °C; **IR** (ATR): ṽ (cm^{-1}) =3209, 3058, 2955, 2850, 2150, 1542, 1493, 1445; **LRMS** (ESI^+, *m/z*) 414.3 $[M+Na]^+$; **HRMS** (ESI^+, m/z): Calc. for $C_{24}H_{25}NO_2SNa$ $[M+Na]^+$ = 414.1498, Found = 414.1497

1-tosyl-*N*-tritylpiperidine-4-sulfinamide 1p

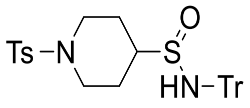

The title compound was prepared according to **General Procedure A** using 1-tosylpiperidine-4-carboxylic acid (0.2 mmol, 57 mg) and *N*-sulfinyltritylamine (0.3 mmol, 92 mg), $Fe(OTf)_3$ (0.03 mmol, 15 mg) and triethylamine (0.04 mmol, 5.4 µL) in DMSO (2 mL). Purification by silica gel flash column chromatography (10-70% EtOAc in isohexane) afforded the *title compound* as a white solid (92 mg, 84%). The spectral data is consistent with the literature.[1]

Scale-up:

The reaction was scaled up in flow carried out in a commercially available instrument—Lumidox II® system (Analytical Sales and Services, New Jersey) with the flow reactor attachment (SKU: 266100) using a ThalesNano H-Cube Pro pump and Humber Minichiller 300 OLE.

A solution of *N*-sulfinyltritylamine (2.20 g, 7.2 mmol), 1-tosylpiperidine-4-carboxylic acid (1.70 g, 6.0 mmol), $Fe(OTf)_3$ (453 mg, 0.9 mol) and TEA (167 µL, 1.20 mmol) in DMSO (100 mL) was irradiated at 405 nm (Lumidox II®, 405 nm, level 5/5, 25 °C external cooling, flow rate = 0.25 mL/min). The resultant solution was diluted with water (250 mL) and extracted with diethyl ether (3 × 250 mL), dried over $MgSO_4$ and concentrated *in vacuo* to afford the crude product. The crude product was purified by column chromatography on silica gel (5-50 % EtOAc/isohexane) to afford 1-tosyl-*N*-tritylpiperidine-4-sulfinamide (2.72 g, 82 %) as a flocculent white solid. The spectral data is consistent with the literature.[1]

^{1}**H NMR** (500 MHz, DMSO) δ 7.64–7.58 (m, 2H), 7.46 (d, J = 8.0 Hz, 2H), 7.38 (s, 1H), 7.31–7.23 (m, 8H), 7.22–7.17 (m, 5H), 3.32 (s, 1H), 3.34–3.26 (m, 2H), 3.08–2.99 (m, 1H), 2.52–2.46 (m, 2H), 2.43 (s, 3H), 1.98–1.86 (m, 2H), 1.56–1.44 (m, 2H); ^{13}**C NMR** (126 MHz, DMSO) δ 145.0, 143.6, 132.2, 129.8, 129.4, 127.6, 127.5, 127.0, 72.7, 58.3, 45.0, 44.8, 25.7, 25.6, 21.0; **IR** (ATR): ṽ (cm^{-1}) =3189, 3061, 2844, 1596, 1490, 1445; **LRMS** (ESI$^+$, *m/z*) 545.2 [M+H]$^+$.

1-(3-methoxybenzoyl)-*N*-tritylpiperidine-4-sulfinamide 1q

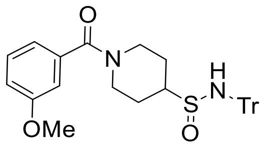

The title compound was prepared according to **General Procedure A** using 1-(3-methoxybenzoyl)piperidine-4-carboxylic acid (0.2 mmol, 53 mg) and *N*-sulfinyltritylamine (0.3 mmol, 92 mg), Fe(OTf)$_3$ (0.03 mmol, 15 mg) and triethylamine (0.04 mmol, 5.4 µL) in DMSO (2 mL). Purification by semi-preparative HPLC (10-40% MeCN) afforded the *title compound* as a white solid (61 mg, 64%, rotamers unresolved at 90 °C).

^{1}**H NMR** (500 MHz, $CDCl_3$) δ 7.36 – 7.23 (m, 17H), 6.96 – 6.87 (m, 2H), 5.06 (s, 1H), 3.84 – 3.77 (m, 5H), 3.04 – 2.95 (m, 2H), 2.91 – 2.81 (m, 1H), 2.06 – 1.96 (m, 2H), 1.71 – 1.53 (m, 2H); ^{13}**C NMR** (126 MHz, $CDCl_3$) δ 170.3, 159.8, 144.7, 137.1, 129.8, 129.5, 128.2, 127.6, 118.9, 115.8, 112.3, 73.3, 62.4, 55.5, 46.9, 41.5, 26.3; **Melting point** (CH_2Cl_2) = 118 – 120 °C; **IR** (ATR): ṽ (cm^{-1}) = 3220, 3058, 2964, 1684, 1576, 1442; **LRMS** (ESI$^+$, *m/z*) 547.3 [M+Na]$^+$; **HRMS** (ESI$^+$, m/z): Calc. for $C_{32}H_{32}N_2O_3SNa$ [M+Na]$^+$ = 547.2026, Found = 547.2018

2-Methyl-*N*-tritylpropane-2-sulfinamide 1r

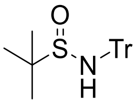

The title compound was prepared according to **General Procedure A** using pivalic acid (0.2 mmol, 20.4 mg) and *N*-sulfinyltritylamine (0.3 mmol, 92 mg), Fe(OTf)$_3$ (0.03 mmol, 15 mg) and triethylamine (0.04 mmol, 5.4 µL) in DMSO (2.0 mL). Purification by silica gel flash column chromatography (15–30% EtOAc in Petrol) afforded the

title compound as a white solid (51 mg, 70%). The spectral data is consistent with the literature.[1]

^{1}H NMR (400 MHz, $CDCl_3$) δ 7.29 – 7.13 (m, 15H), 4.43 (s, 1H), 1.17 (s, 9H); **^{13}C NMR** (101 MHz, $CDCl_3$) δ 145.0, 129.6, 127.9, 127.4, 72.9, 57.2, 23.0; **IR** (ATR): ṽ (cm^{-1}) = 3237, 3062, 2921, 1691, 1597, 1494; **LRMS** (ESI$^+$, m/z) 364.2 [M+H]$^+$; **HRMS** (ESI$^+$, m/z): Calc. for $C_{23}H_{26}NOS$ [M+H]$^+$ = 364.1730, Found = 364.1733.

1-(4-fluorophenyl)-2-methyl-*N*-tritylpropane-2-sulfinamide 1s

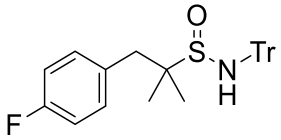

The title compound was prepared according to **General Procedure A** using 3-(4-fluorophenyl)-2,2-dimethylpropanoic acid (0.2 mmol, 39 mg) and *N*-sulfinyltritylamine (0.3 mmol, 92 mg), $Fe(OTf)_3$ (0.03 mmol, 15 mg) and triethylamine (0.04 mmol, 5.4 µL) in DMSO (2 mL). Purification by semi-preparative HPLC (10-40% MeCN) afforded the *title compound* as a colourless oil (17 mg, 19%).

^{1}H NMR (500 MHz, $CDCl_3$) δ 7.36–7.21 (m, 15H), 7.12 (dd, J = 8.3, 5.4 Hz, 2H), 6.95 (t, J = 8.4 Hz, 2H), 4.61 (s, 1H), 2.90 (d, J = 13.4 Hz, 1H), 2.82 (d, J = 13.4 Hz, 1H), 1.19 (s, 3H)), 1.11 (s, 3H); **^{13}C NMR** (126 MHz, $CDCl_3$) δ 162.0 (d, J = 245.1 Hz), 144.9, 132.3 (d, J = 7.8 Hz), 132.1 (d, J = 3.3 Hz), 129.7, 128.0, 127.6, 115.1 (d, J = 21.2 Hz), 73.3, 61.0, 40.7, 20.9, 19.8; **^{19}F NMR** (471 MHz, DMSO) δ -116.82 (tt, J = 9.1, 5.4 Hz); **IR** (ATR): ṽ (cm^{-1}) = 3366 (br.), 3058, 2967, 1599, 1508, 1445; **LRMS** (ESI$^+$, *m/z*) 457.2 [M+H]$^+$; **HRMS** (ESI$^+$, *m/z*) Calc. for $C_{29}H_{28}FNOSK$ [M+K]$^+$ = 496.1507, Found = 496.1512

***N*-trityladamantane-1-sulfinamide 1t**

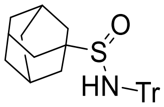

The title compound was prepared according to **General Procedure A** using adamantane-1-carboxylic acid (0.2 mmol, 36 mg) and *N*-sulfinyltritylamine (0.3 mmol, 92 mg), $Fe(OTf)_3$ (0.03 mmol, 15 mg) and triethylamine (0.04 mmol, 5.4 µL) in DMSO (2 mL). Purification by semi-preparative HPLC (10-40% MeCN) afforded the *title*

compound as a white solid (76 mg, 86%). The spectral data is consistent with the literature.[1]

1H NMR (500 MHz, $CDCl_3$) δ 7.38–7.26 (m, 15H), 4.64 (s, 1H), 2.17 (app. s, 3H), 1.93 (d, J = 12.0 Hz, 3H), 1.84 (d, J = 12.0 Hz, 3H), 1.76 (d, J = 12.8 Hz, 3H), 1.71 (d, J = 12.8 Hz, 3H); **13C NMR** (126 MHz, $CDCl_3$) δ 145.1, 129.6, 127.9, 127.4, 72.8, 59.0, 36.5, 35.3, 28.8; **IR** (ATR): ṽ (cm^{-1}) = 3289, 3058, 2907, 2850, 1596, 1491, 1445; **LRMS** (ESI^+, *m/z*) 442.2 $[M+H]^+$.

1-(quinolin-6-yl)-*N*-tritylmethanesulfinamide 1u

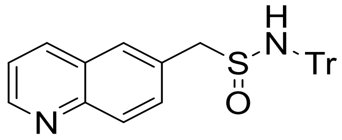

The title compound was prepared according to **General Procedure A** using 2-(quinolin-6-yl)acetic acid (0.2 mmol, 37 mg) and *N*-sulfinyltritylamine (0.3 mmol, 92 mg), $Fe(OTf)_3$ (0.03 mmol, 15 mg) and 1-methylimidazole (0.1 mmol, , 8.0 µL) in DMSO (2 mL). Purification by semi-preparative HPLC (10-40% MeCN) afforded the *title compound* as a yellow oil (47 mg, 52%).

^{1}H NMR (500 MHz, $CDCl_3$) δ 8.94 (dd, J = 4.4, 1.7 Hz, 1H), 8.22–8.18 (m, 1H), 8.17 (d, J = 8.6 Hz, 1H), 7.79 (s, 1H), 7.62 (dd, J = 8.6, 1.9 Hz, 1H), 7.48 (dd, J = 8.3, 4.4 Hz, 1H), 7.23 (dd, J = 5.3, 1.9 Hz, 9H), 7.21–7.16 (m, 6H), 4.83 (s, 1H), 4.14 (d, J = 12.7 Hz, 1H), 4.02 (d, J = 12.7 Hz, 1H); **^{13}C NMR** (126 MHz, $CDCl_3$) δ 149.9, 144.8, 141.2, 137.6, 132.9, 130.2, 129.1, 128.9, 128.7, 128.3, 128.2, 127.6, 121.7, 73.1, 62.3; **IR** (ATR): ṽ (cm^{-1}) = 3412, 3170, 3050, 1599, 1496; **LRMS** (ESI^+, *m/z*) 449.2 $[M+H]^+$; **HRMS** (ESI^+, m/z): Calc. for $C_{29}H_{25}N_2OS$ $[M+H]^+$ = 449.1682, Found = 449.1679

tert-Butyl 2-(((tritylamino)sulfinyl)methyl)-7-azaspiro[3.5]nonane-7-carboxylate 1v

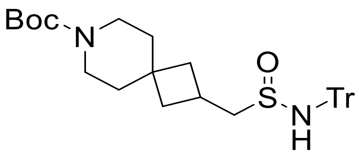

The title compound was prepared according to **General Procedure A** using 2-(7-(tert-butoxycarbonyl)-7-azaspiro[3.5]nonan-2-yl)acetic acid (0.2 mmol, 57 mg) and *N*-sulfinyltritylamine (0.3 mmol, 92 mg), $Fe(OTf)_3$ (0.03 mmol, 15 mg) and triethylamine (0.04 mmol, 5.4 µL) in DMSO (2.0 mL). Purification by silica gel flash column chromatography (15 – 100% EtOAc in Petrol) afforded the title compound as a white solid (48 mg, 44%).

^{1}H NMR (400 MHz, $CDCl_3$) δ 7.39 – 7.24 (m, 15H), 5.00 (s, 1H), 3.37 – 3.21 (m, 4H), 2.97 – 2.84 (m, 1H), 2.83 – 2.74 (m, 1H), 2.73 – 2.61 (m, 1H), 2.07 – 1.96 (m, 2H), 1.58 – 1.51 (m, 3H), 1.46 (s, 12H); **^{13}C NMR** (101 MHz, $CDCl_3$) δ 154.9, 144.9, 129.1, 128.1, 127.4, 79.3, 72.9, 64.6, 39.4 (Rotamer A), 37.9, 37.6, 35.8 (Rotamer B), 35.0, 28.5, 23.7; **Melting point** (CH_2Cl_2) = 126 – 130 °C; **IR** (ATR): ṽ (cm^{-1}) = 2920, 2848, 1677, 1492, 1447; **LRMS** (ESI^+, m/z) 545.5 $[M+H]^+$; **HRMS** (ESI^+, m/z): Calc. for $C_{33}H_{41}N_2O_3S$ $[M+H]^+$ = 545.2832, Found = 545.2820.

Tert-butyl 4-(5-(3-((tritylamino)sulfinyl)bicyclo[1.1.1]pentan-1-yl)-1,2,4-oxadiazol-3-yl)piperidine-1-carboxylate 1w

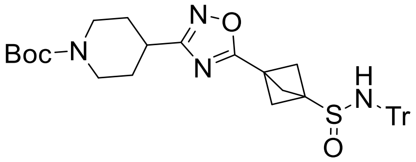

The title compound was prepared according to **General Procedure A** using 3-(3-(1-(tert-butoxycarbonyl)piperidin-4-yl)-1,2,4-oxadiazol-5-yl)bicyclo[1.1.1]pentane-1-carboxylic acid (0.2 mmol, 73 mg) and *N*-sulfinyltritylamine (0.3 mmol, 92 mg), $Fe(OTf)_3$ (0.03 mmol, 15 mg) and triethylamine (0.04 mmol, 5.4 µL) in DMSO (2 mL). Purification by semi-preparative HPLC (10-40% MeCN) afforded the *title compound* as a white solid (98 mg, 78%).

Scale up:

The reaction was scaled up in flow carried out in a commercially available instrument—Lumidox II® system (Analytical Sales and Services, New Jersey) with the flow reactor attachment (SKU: 266100) using a ThalesNano H-Cube Pro pump and Humber Minichiller 300 OLE.

A solution of 3-(3-(1-(tert-butoxycarbonyl)piperidin-4-yl)-1,2,4-oxadiazol-5-yl)bicyclo[1.1.1] pentane-1-carboxylic acid (1.0 g, 2.75 mmol), *N*-sulfinyltritylamine (1.0 g, 3.30 mmol), $Fe(OTf)_3$ (208 mg, 0.41 mmol) and TEA (77 μL, 0.55 mmol) in DMSO (55 mL) was irradiated at 405 nm (Lumidox II®, 405 nm, level 5/5, 40 °C external temperature, flow rate = 0.1 ml/min). The reaction mixture was diluted with sat. aq. $NaHCO_3$ (250 mL), extracted with diethyl ether (3 × 100 mL), dried over $MgSO_4$ and concentrated *in vacuo*. The crude product was purified by chromatography on silica gel (5-70% EtOAc/isohexane) the *title compound* as a white solid (1.27 g, 74%).

^{1}H NMR (500 MHz, $CDCl_3$) δ 7.41–7.19 (m, 15H), 4.91 (d, J = 12.3 Hz, 1H), 4.13 (t, J = 7.1 Hz, 2H), 3.00–2.84 (m, 3H), 2.58–2.52 (m, 6H), 2.01–1.95 (m, 2H), 1.75 (dtd, J = 13.3, 11.5, 4.3 Hz, 2H), 1.46 (s, 9H); **^{13}C NMR** (126 MHz, $CDCl_3$) δ 174.8, 173.1, 154.8, 144.8, 129.2, 128.3, 127.7, 79.8, 53.6, 51.7, 43.5, 41.1, 34.2, 32.8, 29.6, 28.6; **Melting point** (CH_2Cl_2) = 118 – 121 °C; **IR** (ATR): ṽ (cm^{-1}) = 2975, 2927, 1690, 1585, 1448, 1422; **LRMS** (ESI^+, *m/z*) 647.2 $[M+Na]^+$; **HRMS** (ESI^+, m/z): Calc. for $C_{36}H_{40}N_4O_4SNa$ $[M+Na]^+$ = 647.2663, Found = 647.2676

(3R)-3-((3R,5S,7R,8R,10S,13R,14S,17R)-3,7-dihydroxy-10,13-dimethylhexadecahydro-1H-cyclopenta[a]phenanthren-17-yl)-*N*-tritylbutane-1-sulfinamide 1x

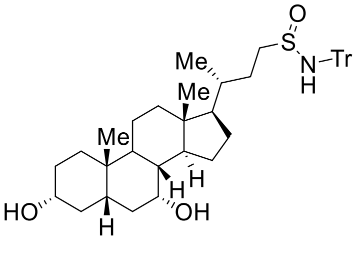

The title compound was prepared according to **General Procedure A** using chenodeoxycholic acid (0.2 mmol, 79 mg) and *N*-sulfinyltritylamine (0.3 mmol, 92 mg), $Fe(OTf)_3$ (0.03 mmol, 15 mg) and triethylamine (0.04 mmol, 5.6 μL) in DMSO (2 mL). Purification by silica gel flash column chromatography (10-50% EtOAc in isohexane) afforded the *title compound* as a white solid (120 mg, 94%, 1:1 inseparable mixture of diastereomers). The spectral data is consistent with the literature.[1]

^{1}H NMR (400 MHz, $CDCl_3$) δ 7.38 – 7.23 (m, 15H), 4.88 (s, 0.5H), 4.87 (s, 0.5H), 3.89 – 3.79 (m, 1H), 3.51 – 3.41 (m, 1H), 2.80 – 2.68 (m, 1H), 2.69 – 2.55 (m, 1H), 2.20 (q, J = 12.4 Hz, 1H), 2.01 – 1.90 (m, 2H), 1.86 – 1.76 (m, 4H), 1.76 – 1.56 (m, 4H), 1.55

– 1.42 (m, 4H), 1.41 – 1.06 (m, 10H), 1.00 – 0.80 (m, 7H), 0.63 (s, 3H); **^{13}C NMR** (101 MHz, $CDCl_3$) δ 145.0, 145.0, 129.4, 129.3, 128.2, 128.1, 127.5, 127.5, 73.0, 72.1, 68.6, 55.9, 55.7, 55.1, 55.0, 50.5, 42.8, 41.6, 40.0, 39.7, 39.5, 35.5, 35.3, 35.2, 35.1, 34.8, 34.8, 32.9, 30.8, 30.4, 29.8, 29.5, 29.2, 28.3, 28.2, 23.8, 22.9, 20.7, 18.7, 18.6, 11.9, 11.9. *(13 carbon signals are unresolved from the corresponding signals in the other diastereomer)*; **LRMS** (ESI$^+$, *m/z*) 676.4 [M+Na]$^+$; **HRMS** (ESI$^+$, m/z): Calc. for $C_{42}H_{55}NO_3SNa$ [M+Na]$^+$ = 676.3795, Found = 676.3790.

2-phenyl-*N*-(2,4,4-trimethylpentan-2-yl)ethane-1-sulfinamide 2a

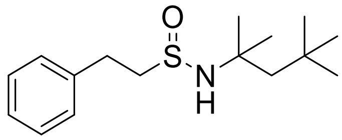

The title compound was prepared according to **General Procedure A** using hydrocinnamic acid (0.2 mmol, 30 mg) and *N*-sulfinyl-tert-ocytylamine (0.3 mmol, 53 mg), $Fe(OTf)_3$ (0.03 mmol, 15 mg) and triethylamine (0.04 mmol, 5.4 µL) in DMSO (2 mL). Purification by silica gel flash column chromatography (10-50% EtOAc in isohexane) afforded the *title compound* as a white solid (55 mg, 98%). The spectral data is consistent with the literature.[1]

^{1}H NMR (400 MHz, $CDCl_3$) δ 7.28 – 7.21 (m, 2H), 7.19 – 7.13 (m, 3H), 3.57 (s, 1H), 2.99 – 2.82 (m, 4H), 1.45 (s, 3H), 1.31 (s, 6H), 0.93 (s, 9H); **^{13}C NMR** (101 MHz, $CDCl_3$) δ 139.2, 128.7, 128.5, 126.6, 58.4, 57.9, 56.1, 32.3, 31.8, 31.8, 29.4, 29.4; **IR** (ATR): ṽ (cm^{-1}) = 3175, 2951, 2868, 1604, 1496, 1455; **LRMS** (ESI$^+$, *m/z*) 304.2 [M+Na]$^+$.

***N*-(tert-butoxy)-2-phenylethane-1-sulfinamide 2b**

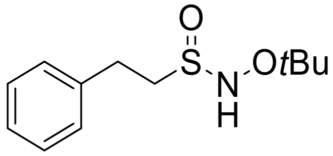

The title compound was prepared according to **General Procedure A** using hydrocinnamic acid (0.2 mmol, 30 mg) and *N*-sulfinyl-*O*-tert-butyl (0.3 mmol, 41 mg), $Fe(OTf)_3$ (0.03 mmol, 15 mg) and triethylamine (0.04 mmol, 5.4 µL) in DMSO (2 mL).

Purification by silica gel flash column chromatography (10-50% EtOAc in isohexane) afforded the *title compound* as a white solid (47 mg, 98%). The spectral data is consistent with the literature.[1]

^{1}H NMR (400 MHz, $CDCl_3$) δ 7.30 – 7.23 (m, 2H), 7.20 – 7.15 (m, 3H), 6.75 (s, 1H), 3.05 – 2.93 (m, 4H), 1.15 (s, 9H); **^{13}C NMR** (101 MHz, $CDCl_3$) δ 138.7, 128.8, 128.5, 126.8, 80.3, 52.5, 28.9, 26.6; **IR** (ATR): ṽ (cm^{-1}) = 3347, 2971, 2929, 2884, 1649, 1464; **LRMS** (ESI^+, *m/z*) 264.0 $[M+Na]^+$

2-phenyl-*N*-(triisopropylsilyl)ethane-1-sulfinamide 2c

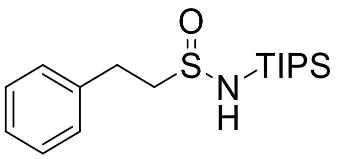

The title compound was prepared according to **General Procedure B** using hydrocinnamic acid (0.3 mmol, 45 mg), *N*-sulfinyl-triisopropylsilylamine (0.2 mmol, 44.0 mg), $Fe(NO_3)_3{\cdot}9H_2O$ (0.045 mmol, 18 mg) and triethylamine (0.06 mmol, 8.4 μL) in DMSO (2 mL). Purification by silica gel flash column chromatography (10-50% EtOAc in isohexane) afforded the *title compound* as a colourless oil (64 mg, 98%). The spectral data is consistent with the literature.[1]

^{1}H NMR (400 MHz, $CDCl_3$) δ 7.27 – 7.13 (m, 5H), 3.41 (s, 1H), 3.06 – 2.86 (m, 4H), 1.15 – 1.04 (m, 3H), 1.02 – 0.96 (m, 18H); **^{13}C NMR** (101 MHz, $CDCl_3$) δ 139.2, 128.8, 128.5, 126.6, 61.7, 29.3, 17.9, 17.8, 11.7; **IR** (ATR): ṽ (cm^{-1}) = 3168, 3029, 2943, 1604, 1497, 1462; **LRMS** (ESI^+, *m/z*): 326.7 $[M+H]^+$.

Methyl 3-(((triisopropylsilyl)amino)sulfinyl)propanoate 3a

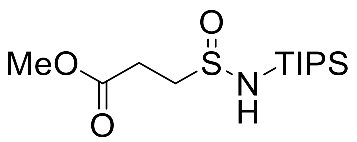

The title compound was prepared according to **General Procedure A** using 4-methoxy-4-oxobutanoic acid (0.3 mmol, 92 mg), *N*-sulfinyl-triisopropylsilylamine (0.2 mmol, 44.0 mg), $Fe(NO_3)_3{\cdot}9H_2O$ (0.045 mmol, 18 mg) and triethylamine (0.06 mmol, 8.4 μL) in DMSO (2 mL). Purification by silica gel flash column chromatography (10-50% EtOAc in isohexane) afforded the *title compound* as a colourless oil (56 mg, 91%).

^{1}H NMR (400 MHz, $CDCl_3$) δ 3.95 (s, 1H), 3.65 (s, 3H), 3.08 – 2.97 (m, 1H), 2.97 – 2.85 (m, 1H), 2.85 – 2.75 (m, 1H), 2.72 – 2.59 (m, 1H), 1.16 – 1.05 (m, 3H), 1.05 – 0.99 (m, 18H); **^{13}C NMR** (101 MHz, $CDCl_3$) δ 172.5, 54.1, 52.1, 27.4, 17.8, 17.8, 11.7**;** **IR** (ATR): ṽ (cm^{-1}) =3198, 2947, 2868, 1742; **LRMS** (ESI$^+$, *m/z*): 308.2 $[M+H]^+$; **HRMS** (ESI$^+$, m/z): Calc. for $C_{13}H_{29}NO_3SSiNa$ $[M+Na]^+$ = 330.1530, Found = 330.1526.

Tert-butyl (2-(((triisopropylsilyl)amino)sulfinyl)ethyl)carbamate 3b

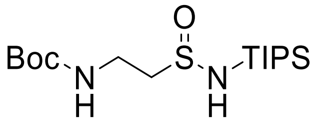

The title compound was prepared according to **General Procedure A** using 3-((tert-butoxycarbonyl)amino)propanoic acid (0.3 mmol, 109 mg), *N*-sulfinyl-triisopropylsilylamine (0.2 mmol, 44.0 mg), $Fe(NO_3)_3 \cdot 9H_2O$ (0.045 mmol, 18 mg) and triethylamine (0.06 mmol, 8.4 µL) in DMSO (2 mL). Purification by silica gel flash column chromatography (10-50% EtOAc in isohexane) afforded the *title compound* as a colourless oil (66 mg, 91%).

^{1}H NMR (400 MHz, $CDCl_3$) δ 5.25 (s, 1H), 3.68 (s, 1H), 3.58 – 3.43 (m, 2H), 3.09 – 2.99 (m, 1H), 2.75 – 2.66 (m, 1H), 1.36 (s, 9H), 1.17 – 1.05 (m, 3H), 1.05 – 0.98 (m, 18H); **^{13}C NMR** (101 MHz, $CDCl_3$) δ 13C NMR (101 MHz, CDCl3) δ 155.9, 79.6, 59.4, 35.6, 28.4, 17.8, 17.8, 11.6; **IR** (ATR): ṽ (cm^{-1}) = 3211, 2948, 2868, 1743; **LRMS** (ESI$^+$, *m/z*) 365.3 $[M+H]^+$; **HRMS** (ESI$^+$, m/z): Calc. for $C_{16}H_{37}N2O_3SSi$ $[M+H]^+$ = 365.2289, Found = 365.2297.

***N*-(Triisopropylsilyl)hexane-1-sulfinamide 3c**

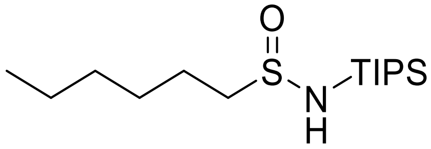

The title compound was prepared according to **General Procedure A** using heptanoic acid (0.3 mmol, 92 mg), *N*-sulfinyl-triisopropylsilylamine (0.2 mmol, 44.0 mg), $Fe(NO_3)_3 \cdot 9H_2O$ (0.045 mmol, 18 mg) and triethylamine (0.06 mmol, 8.4 µL) in DMSO

(2 mL). Purification by silica gel flash column chromatography (10-50% EtOAc in isohexane) afforded the title compound as a colourless oil (48 mg, 78%).

^{1}H NMR (400 MHz, $CDCl_3$) δ 3.35 (s, 1H), 2.65 (t, J = 7.7 Hz, 2H), 1.69 – 1.57 (m, 2H), 1.43 – 1.31 (m, 2H), 1.26 – 1.22 (m, 4H), 1.15 – 1.06 (m, 3H), 1.06 – 0.97 (m, 18H), 0.85 – 0.79 (m, 3H); **^{13}C NMR** (101 MHz, $CDCl_3$) δ 61.2, 31.4, 28.4, 23.3, 22.4, 17.9, 17.8, 14.0, 11.7; **IR** (ATR): ṽ (cm^{-1}) = 3158, 2959, 2868, 1695, 1465
LRMS (ESI$^+$, m/z) 306.1 [M+H]$^+$; **HRMS** (ESI$^+$, m/z): Calc.for $C_{15}H_{36}NOSSi$ [M+H]$^+$ = 306.2281, Found = 306.2293.

3-Methyl-*N*-(triisopropylsilyl)butane-1-sulfinamide 3d

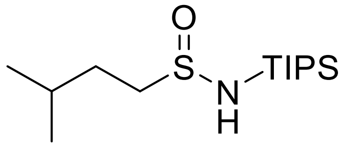

The title compound was prepared according to **General Procedure A** using 3-methylbutanoic acid (0.3 mmol, 87 mg), *N*-sulfinyl-triisopropylsilylamine (0.2 mmol, 44.0 mg), $Fe(NO_3)_3{\cdot}9H_2O$ (0.045 mmol, 18 mg) and triethylamine (0.06 mmol, 8.4 μL) in DMSO (2 mL). Purification by silica gel flash column chromatography (10-50% EtOAc in isohexane) afforded the title compound as a colourless oil (37 mg, 63%).

^{1}H NMR (400 MHz, $CDCl_3$) δ 3.36 (s, 1H), 2.66 (t, J = 7.9 Hz, 2H), 1.69 – 1.57 (m, 1H), 1.57 – 1.46 (m, 2H), 1.17 – 1.06 (m, 3H), 1.06 – 0.97 (m, 18H), 0.91 – 0.85 (m, 6H); **^{13}C NMR** (101 MHz, $CDCl_3$) δ 59.2, 31.8, 27.5, 22.4, 22.2, 17.9, 17.8, 11.7; **IR** (ATR): ṽ (cm^{-1}) = 3152, 2950, 2852, 1496
LRMS (ESI$^+$, m/z) 292.1 [M+H]$^+$; **HRMS** (ESI$^+$, m/z): Calc. for $C_{14}H_{34}NOSSi$ [M+H] = 292.2125, Found = 292.2131.

***N*-(Triisopropylsilyl)propane-2-sulfinamide 3e**

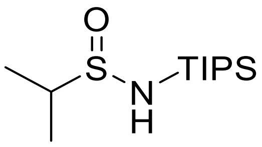

The title compound was prepared according to **General Procedure A** using isobutyric acid (0.3 mmol, 79 mg), *N*-sulfinyl-triisopropylsilylamine (0.2 mmol, 44.0 mg), $Fe(NO_3)_3{\cdot}9H_2O$ (0.045 mmol, 18 mg) and triethylamine (0.06 mmol, 8.4 µL) in DMSO (2 mL). Purification by silica gel flash column chromatography (10-100% EtOAc in isohexane) afforded the title compound as a colourless oil (37 mg, 71%).

^{1}H NMR (400 MHz, $CDCl_3$) δ 3.15 (s, 1H), 2.58 (hept, J = 6.9 Hz, 1H), 1.19 (dd, J = 6.9, 1.8 Hz, 6H), 1.17 – 1.06 (m, 3H), 1.07 – 0.98 (m, 18H); **^{13}C NMR** (101 MHz, $CDCl_3$) δ 57.0, 17.9, 17.8, 15.0, 14.9, 11.8; **IR** (ATR): ṽ (cm^{-1}) = 3150, 2944, 2892, 2867, 1464; **LRMS** (ESI^+, m/z) 264.5 $[M+H]^+$; **HRMS** (ESI^+, m/z): Calc. for $C_{12}H_{30}NOSSi$ [M+H] = 264.1811, Found = 264.1804.

N-(Triisopropylsilyl)cyclopentanesulfinamide 3f

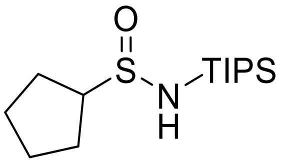

The title compound was prepared according to **General Procedure A** using cyclopentanecarboxylic acid (0.3 mmol, 87 mg), *N*-sulfinyl-triisopropylsilylamine (0.2 mmol, 44.0 mg), $Fe(NO_3)_3{\cdot}9H_2O$ (0.045 mmol, 18 mg) and triethylamine (0.06 mmol, 8.4 µL) in DMSO (2 mL). Purification by silica gel flash column chromatography (10-50% EtOAc in isohexane) afforded the title compound as a colourless oil (40 mg, 70%).

^{1}H NMR (400 MHz, $CDCl_3$) δ 3.20 (s, 1H), 2.92 (tt, J = 8.6, 6.3 Hz, 1H), 2.04 – 1.95 (m, 1H), 1.91 – 1.80 (m, 2H), 1.78 – 1.53 (m, 5H), 1.16 – 1.06 (m, 3H), 1.06 – 0.99 (m, 18H); **^{13}C NMR** (101 MHz, $CDCl_3$) δ 68.0, 27.8, 26.0, 25.7, 25.6, 17.9, 17.8, 11.8; **IR** (ATR): ṽ (cm^{-1}) = 3173, 2945, 2892, 2867, 1676, 1464; **LRMS** (ESI^+, m/z) 290.6 $[M+H]^+$; **HRMS** (ESI^+, m/z): Calc. for $C_{14}H_{32}NOSSi$ [M+H] = 290.1968, Found = 290.1973.

N-(Triisopropylsilyl)cyclohexanesulfinamide 3g

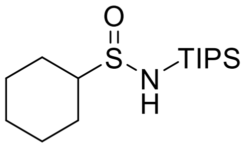

The title compound was prepared according to **General Procedure A** using cyclohexanecarboxylic acid (0.3 mmol, 91 mg), *N*-sulfinyl-triisopropylsilylamine (0.2 mmol, 44.0 mg), $Fe(NO_3)_3 \cdot 9H_2O$ (0.045 mmol, 18 mg) and triethylamine (0.06 mmol, 8.4 µL) in DMSO (2 mL). Purification by silica gel flash column chromatography (10-50% EtOAc in isohexane) afforded the title compound as a colourless oil (53 mg, 88%).

^{1}H NMR (400 MHz, $CDCl_3$) δ 3.21 (s, 1H), 2.36 – 2.24 (m, 1H), 2.04 – 1.93 (m, 2H), 1.88 – 1.75 (m, 2H), 1.66 – 1.58 (m, 1H), 1.32 – 1.22 (m, 5H), 1.16 – 1.07 (m, 3H), 1.05 – 0.98 (m, 18H); **^{13}C NMR** (101 MHz, $CDCl_3$) δ 66.4, 26.1, 25.7, 25.6, 25.5, 25.3, 17.9, 17.8, 11.8; **IR** (ATR): ṽ (cm^{-1}) = 2931, 2867, 1464, 1384; **LRMS** (ESI^+, m/z) 304.2 $[M+H]^+$; **HRMS** (ESI^+, m/z): Calc. for $C_{15}H_{34}NOSSi$ $[M+H]^+$ = 304.2125, Found = 304.2121.

2-Methyl-*N*-(triisopropylsilyl)propane-2-sulfinamide 3h

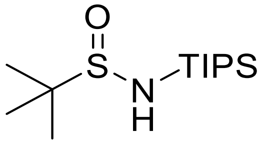

The title compound was prepared according to **General Procedure A** using pivalic acid (0.3 mmol, 83 mg), *N*-sulfinyl-triisopropylsilylamine (0.2 mmol, 44.0 mg), $Fe(NO_3)_3 \cdot 9H_2O$ (0.045 mmol, 18 mg) and triethylamine (0.06 mmol, 8.4 µL) in DMSO (2 mL). Purification by silica gel flash column chromatography (10-100% EtOAc in isohexane) afforded the title compound as a colourless oil (38 mg, 68%).

^{1}H NMR (400 MHz, $CDCl_3$) δ 2.98 (s, 1H), 1.21-1.09 (m, 12H), 1.04 (dd, J = 8.9, 6.9 Hz, 18H); **^{13}C NMR** (101 MHz, $CDCl_3$) δ 56.5, 22.4, 17.9, 17.8, 11.9; **IR** (ATR): ṽ (cm^{-1}) = 2982, 2867, 1480; **LRMS** (ESI^+, m/z) 278.1 $[M+H]^+$; **HRMS** (ESI^+, m/z): Calc. for $C_{13}H_{31}NOSSiNa$ [M+Na] = 300.1788, Found = 300.1783.

tert-butyl (tert-butoxycarbonyl)(((triisopropylsilyl)amino)sulfinyl)-D-alaninate 3i

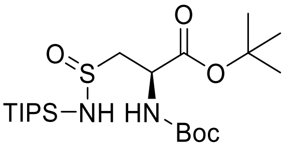

The title compound was prepared according to **General Procedure B** using (S)-3-((tert-butoxycarbonyl)amino)-4-methoxy-4-oxobutanoic acid (0.30 mmol, 74 mg), *N*-

sulfinyl-triisopropylsilylamine (0.2 mmol, 44.0 mg), $Fe(NO_3)_3 \cdot 9H_2O$ (0.045 mmol, 18 mg) and triethylamine (0.06 mmol, 8.4 µL) in DMSO (2 mL). Purification by silica gel flash column chromatography (10-100% EtOAc in isohexane) afforded the *title compound* as a colourless oil (76 mg, 82%, 1:1 mixture of inseparable diastereomers).

^{1}H NMR (400 MHz, $CDCl_3$) δ 5.74 (s, 0.5 H), 5.53 (s, 0.5 H), 4.54 – 4.41 (m, 1H), 4.15 (s, 0.5 H), 4.05 (s, 0.5 H), 3.37 – 3.31 (m, 0.5 H), 3.31 – 3.24 (m, 0.5 H), 3.16 – 3.04 (m, 0.5 H), 2.95 (dd, J = 13.2, 4.6 Hz, 0.5 H), 1.42 (s, 4.5 H), 1.41 (s, 4.5 H), 1.37 (s, 9H), 1.15 – 1.06 (m, 3H), 1.06 – 0.97 (m, 18H); **^{13}C NMR** (101 MHz, $CDCl_3$) δ 170.1, 154.0, 83.1, 82.9, 77.4, 66.0, 66.0, 61.0, 60.9, 28.4, 28.1, 18.0, 17.9, 17.9, 17.9, 11.8. *(5 carbon signals are unresolved from the corresponding signals in the other diastereomer)*; **IR** (ATR): ṽ (cm^{-1}) = 3245, 2964, 2944, 2869, 1718, 1506, 1460, 1393, 1368; **LRMS** 465.2 $[M+H]^+$; **HRMS** (ESI^+, m/z): Calc. for $C_{21}H_{45}N_2O_5SSi$ $[M+H]^+$ = 465.2813, Found = 465.2808.

tert-butyl (2S)-2-((tert-butoxycarbonyl)amino)-4-(((triisopropylsilyl)amino)sulfinyl)butanoate 3j

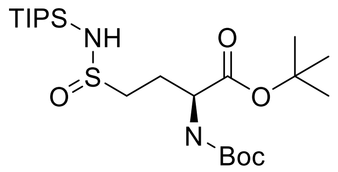

The title compound was prepared according to **General Procedure B** using (S)-5-(tert-butoxy)-4-((tert-butoxycarbonyl)amino)-5-oxopentanoic acid (0.3 mmol, 91 mg), *N*-sulfinyl-triisopropylsilylamine (0.2 mmol, 44.0 mg), $Fe(NO_3)_3 \cdot 9H_2O$ (0.045 mmol, 18 mg) and triethylamine (0.06 mmol, 8.4 µL) in DMSO (3 mL). Purification by silica gel flash column chromatography (10-100% EtOAc in isohexane) afforded the title compound as a colourless oil (71 mg, 74%, 1:1 mixture of inseparable diastereomers).

^{1}H NMR (400 MHz, $CDCl_3$) δ 5.30 (d, J = 7.8 Hz, 0.5H), 5.20 (d, J = 8.2 Hz, 0.5H), 4.34 – 4.24 (m, 1H), 3.79 (s, 0.5H), 3.69 (s, 0.5H), 2.90 – 2.65 (m, 2H), 2.28 – 2.16 (m, 1H), 2.06 – 1.88 (m, 1H), 1.46 (s, 9H), 1.43 (s, 9H), 1.22 – 1.12 (m, 3H), 1.10 – 1.04 (m, 18H); **^{13}C NMR** (101 MHz, $CDCl_3$) δ 171.1, 171.1, 155.6, 155.6, 82.7, 82.7, 80.2, 80.1, 56.3, 56.2, 53.2, 53.0, 28.4, 28.1, 27.1, 26.6, 18.0, 18.0, 17.9, 17.9, 11.9, 11.8 *(2 carbon signals are unresolved from the corresponding signals in the other diastereomer)*; **IR** (ATR): ṽ (cm^{-1}) = 2964, 2929, 2868, 1720, 1700, 1459, 1368, 1288;

LRMS 479.3 [M+H]$^+$; **HRMS** (ESI$^+$, m/z): Calc. for $C_{22}H_{47}N_2O_5SSi$ [M+H]$^+$ = 479.2969, Found = 479.2959.

tert-butyl 4-(5-(3-(((triisopropylsilyl)amino)sulfinyl)bicyclo[1.1.1]pentan-1-yl)-1,2,4-oxadiazol-3-yl)piperidine-1-carboxylate 3k

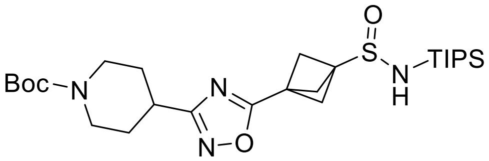

The title compound was prepared according to **General Procedure B** using 3-(3-(1-(tert-butoxycarbonyl)piperidin-4-yl)-1,2,4-oxadiazol-5-yl)bicyclo[1.1.1]pentane-1-carboxylic acid (0.30 mmol, 0.11 g), *N*-sulfinyl-triisopropylsilylamine (0.2 mmol, 44.0 mg), $Fe(NO_3)_3{\cdot}9H_2O$ (0.045 mmol, 18 mg) and triethylamine (0.06 mmol, 8.4 μL) in DMSO (2 mL). Purification by silica gel flash column chromatography (10-100% EtOAc in isohexane) afforded the *title compound* as a colourless oil (87 mg, 75%).

^{1}H NMR (400 MHz, $CDCl_3$) δ 4.19 – 4.06 (m, 2H) 3.32 (s, 1H), 2.99 – 2.85 (m, 3H) 2.55 – 2.46 (m, 6H) 1.96 (d, J = 13.2 Hz, 2H) 1.82 – 1.70 (m, 2H) 1.45 (s, 9H) 1.25 – 1.14 (m, 3H) 1.13 – 1.04 (m, 18H); **^{13}C NMR** (101 MHz, $CDCl_3$) δ 173.1, 170.5, 155.0, 79.8, 77.4, 51.1, 44.0, 34.3, 29.6, 29.4, 28.6, 17.9, 17.9, 11.8; **IR** (ATR): ṽ (cm^{-1}) = 3473, 3200, 2945, 2867, 1697, 1587, 1519, 1451, 1366; **LRMS** 539.2 [M+H]$^+$; **HRMS** (ESI$^+$, m/z): Calc. for $C_{26}H_{46}N_4O_4SSiNa$ [M+Na]$^+$ = 561.2901, Found = 561.2909.

Tert-butyl 4-(5-(3-(S-fluoro-*N*-tritylsulfonimidoyl)bicyclo[1.1.1]pentan-1-yl)-1,2,4-oxadiazol-3-yl)piperidine-1-carboxylate 5

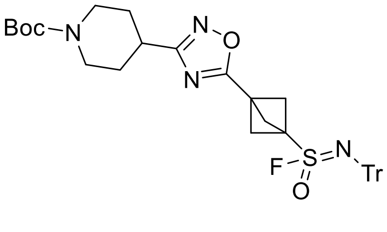

To a stirred solution of tert-butyl 4-(5-(3-((tritylamino)sulfinyl)bicyclo[1.1.1]pentan-1-yl)-1,2,4-oxadiazol-3-yl)piperidine-1-carboxylate (300 mg, 0.47 mmol) in anhydrous MeCN (5 mL) was added Trichlorocyanuric acid (26 μL, 0.2 mmol). The resultant solution was stirred for 30 mins. The reaction mixture was charged with silver(I) fluoride (66 mg, 0.5 mmol) and stirred for a further 1 hr at room temperature.

The resultant suspension was diluted with DCM (10 mL) and washed with water (3 × 5 mL), dried over $MgSO_4$ and concentrated *in vacuo* to afford the crude product.

The crude product was purified by chromatography on silica gel (0-50% EtOAc/isohexane) to afford tert-butyl 4-(5-(3-(S-fluoro-*N*-tritylsulfonimidoyl)bicyclo[1.1.1]pentan-1-yl)-1,2,4-oxadiazol-3-yl)piperidine-1-carboxylate as a white solid (235 mg, 78 %).

^{1}H NMR (500 MHz, $CDCl_3$) δ 7.38–7.33 (m, 6H), 7.33–7.22 (m, 9H), 4.16–4.10 (m, 1H), 3.00–2.91 (m, 1H), 2.92–2.89 (m, 2H), 2.86 (s, 6H), 2.00–1.95 (m, 2H), 1.81–1.70 (m, 2H), 1.47 (s, 9H); **^{13}C NMR** (126 MHz, $CDCl_3$) δ 173.9 (rotamer 1), 173.2 (rotamer 2), 154.8, (d, J = 4.0 Hz), 128.6, 128.0, 127.3, 79.8, 73.6 (d, J = 4.7 Hz), 54.9, 52.4 (d, J = 34.2 Hz), 49.6, 43.3, 34.2A, 32.0, 29.6, 28.6, 27.1; **^{19}F NMR** (471 MHz, $CDCl_3$) δ 71.14; **Melting point** (CH_2Cl_2) = 106 – 109 °C; **IR** (ATR): ṽ (cm^{-1}) = 2941, 2888, 1689, 1591, 1452; **LRMS** (ESI^+, *m/z*) 543.3 $[M\text{-}Boc]^+$; **HRMS** (ESI^+, m/z): Calc. for $C_{36}H_{39}FN_4O_4SNa$ $[M+Na]^+$ = 665.2573, Found = 665.2600.

Tert-butyl 4-(3-(3-(*N*-trityl-1H-imidazole-1-sulfonimidoyl)bicyclo[1.1.1]pentan-1-yl)-1,2,4-oxadiazol-5-yl)piperidine-1-carboxylate 6

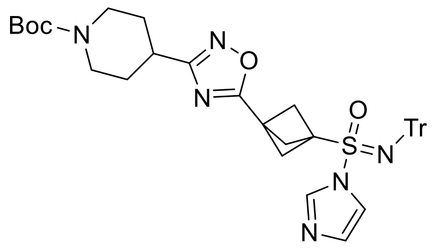

To a solution of tert-butyl 4-(3-(3-((tritylamino)sulfinyl)bicyclo[1.1.1]pentan-1-yl)-1,2,4-oxadiazol-5-yl)piperidine-1-carboxylate (510 mg, 0.8 mmol) in anhydrous THF (5 mL) under N_2 was added trichlorocyanuric acid (95 mg, 0.4 mmol). The resultant solution was stirred at r.t. for 20 mins. The reaction mixture was charged with imidazole (556 mg, 8.2 mmol) and heated to reflux and stirred for 2 hr.

The reaction mixture was diluted with DCM (25 mL) and sat. aq. NH_4Cl (25 mL). The phases were separated and the aqueous was extracted with DCM (3 × 10 mL). The combined organics were washed with brine (2 × 25 mL), dried over $MgSO_4$ and concentrated *in vacuo* to afford a white oil which upon trituration with diethyl ether afforded the *title compound* as a white solid (542 mg, 98 %).

^{1}H NMR (500 MHz, $CDCl_3$, 363.2 K) δ 7.36 – 7.30 (m, 7H), 7.26 – 7.15 (m, 9H), 6.88 (app. s, 1H), 6.79 (app. s, 1H), 3.91 (dt, J = 13.4, 3.8 Hz, 2H), 3.07 – 3.00 (m, 1H), 2.96 (td, J = 13.4, 3.0 Hz, 2H), 2.70 (dd, J = 9.2, 1.7 Hz, 3H), 2.64 (dd, J = 9.2, 1.7 Hz, 3H), 1.97 – 1.88 (m, 2H), 1.63 – 1.52 (m, 2H), 1.42 (s, 9H); **^{13}C NMR** (126 MHz, $CDCl_3$, 363.2 K) δ 173.9 (rotamer 1), 172.8 (rotamer 1), 154.0, 145.8, 136.8, 129.1, 128.2, 127.6, 126.7, 118.3, 78.7, 72.2, 55.5, 53.6, 42.8, 33.0, 30.2, 29.1, 28.1; **Melting point** (CH_2Cl_2) = 114 – 116 °C; **IR**: ṽ =2975, 2927, 1687, 1587, 1475; **LRMS** 590.3 [M-Boc]$^+$; **HRMS** (ESI$^+$, m/z): Calc. for $C_{39}H_{42}N_6O_4SNa$ [M+Na] = 713.2881, Found = 713.2848

tert-butyl 4-(5-(3-(*N*-tritylsulfamoyl)bicyclo[1.1.1]pentan-1-yl)-1,2,4-oxadiazol-3-yl)piperidine-1-carboxylate 7

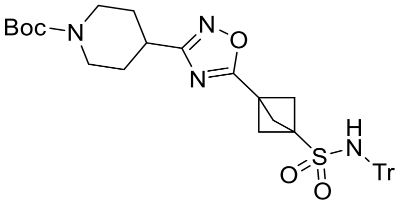

To a stirred solution of tert-butyl 4-(5-(3-((tritylamino)sulfinyl)bicyclo[1.1.1]pentan-1-yl)-1,2,4-oxadiazol-3-yl)piperidine-1-carboxylate (125 mg, 0.2 mmol) in anhydrous MeCN (2 mL) was added Trichlorocyanuric acid (23.2 mg, 0.1 mmol). The resultant solution was stirred for 30 mins. The reaction mixture was quenched with water and stirred overnight room temperature.

The resultant suspension was diluted with diethyl ether (25 mL) and washed with sat. aq. NH_4Cl (3 × 10 mL), dried over $MgSO_4$ and concentrated *in vacuo* to the title compound as a white solid (125 mg, 98 %).

^{1}H NMR (400 MHz, $CDCl_3$) δ 7.45 – 7.31 (m, 15H), 5.50 (s, 1H), 4.17 – 4.10 (m, 2H), 2.98 – 2.85 (m, 3H), 2.25 (s, 6H), 2.00 – 1.92 (m, 2H), 1.81 – 1.66 (m, 2H), 1.48 (s, 9H); **^{13}C NMR** (101 MHz, CDCl3) δ 173.2, 154.8, 146.2, 146.2, 128.6, 128.0, 127.3, 79.8, 73.6, 54.9, 43.3, 34.2, 32.0, 29.6, 28.6, 27.1; **Melting point** (CH_2Cl_2) = 94 – 98 °C; **IR**: ṽ = 2946, 2867, 1693, 1590, 1449; **LRMS** 541.1 [M-Boc]$^+$; **HRMS** (ESI$^+$, m/z): Calc. for $C_{36}H_{40}N_4O_5SNa$ [M+Na]$^+$ = 663.2612, Found = 663.2629.

4. References

(1) Andrews, J. A.; Kalepu, J.; Palmer, C. F.; Poole, D. L.; Christensen, K. E.; Willis, M. C. Photocatalytic Carboxylate to Sulfinamide Switching Delivers a Divergent Synthesis of Sulfonamides and Sulfonimidamides. *J. Am. Chem. Soc.* **2023**, *145* (39), 21623–21629. https://doi.org/10.1021/jacs.3c07974.

5. NMR Spectra

2-phenyl-*N*-tritylethane-1-sulfinamide 1a

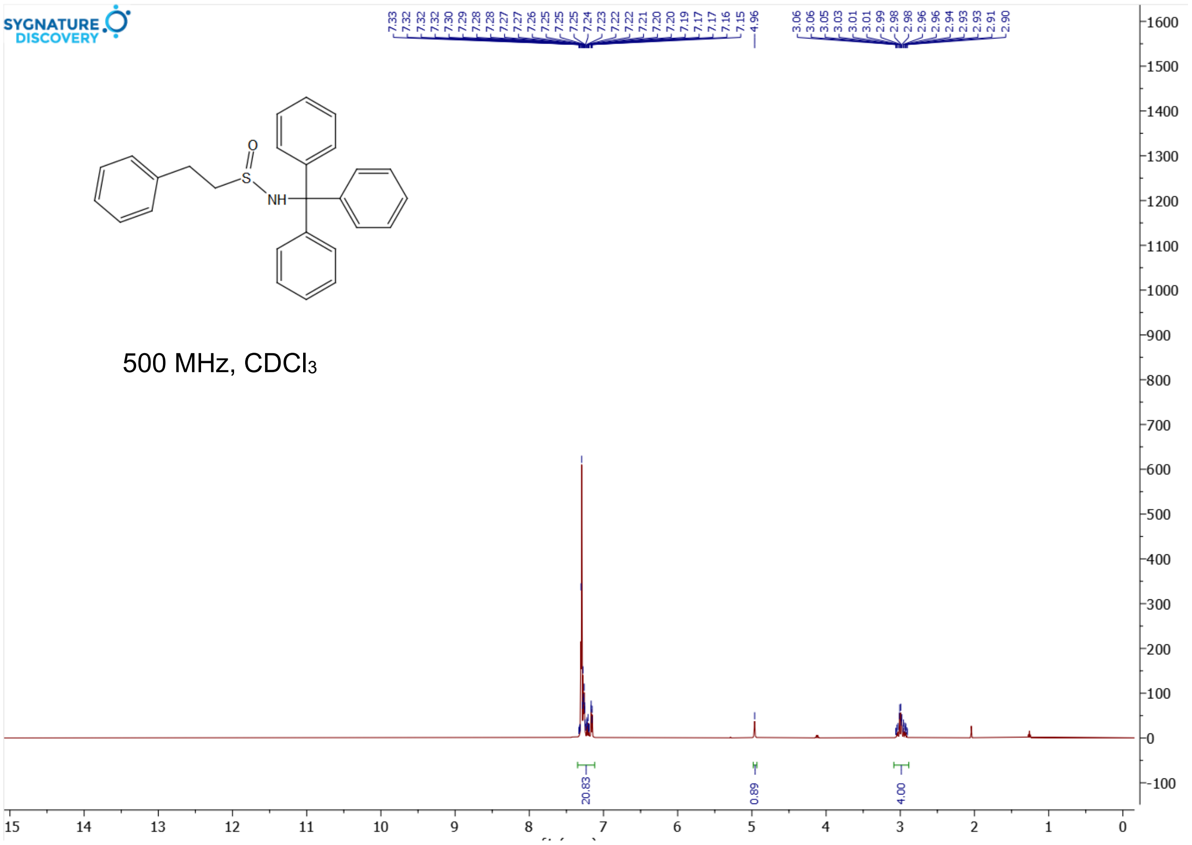

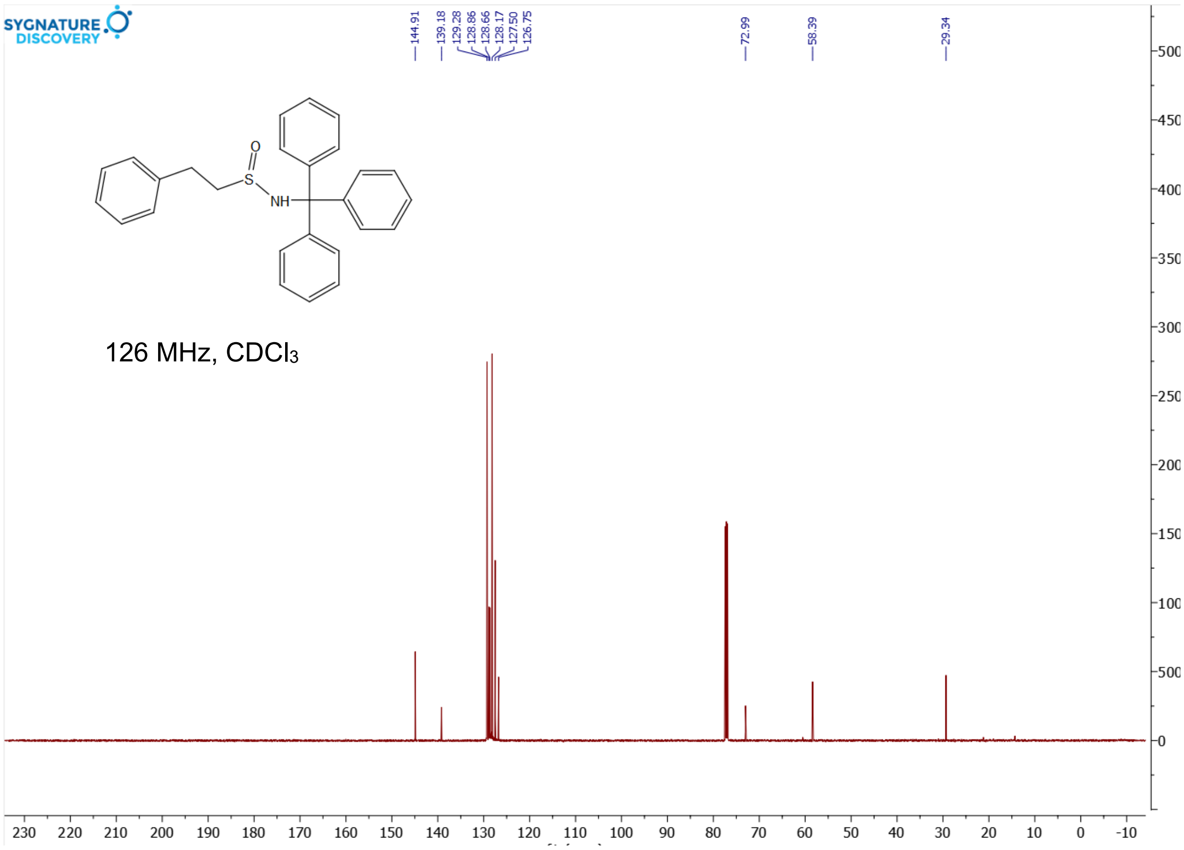

2-(pyridin-3-yl)-*N*-tritylethane-1-sulfinamide 1b

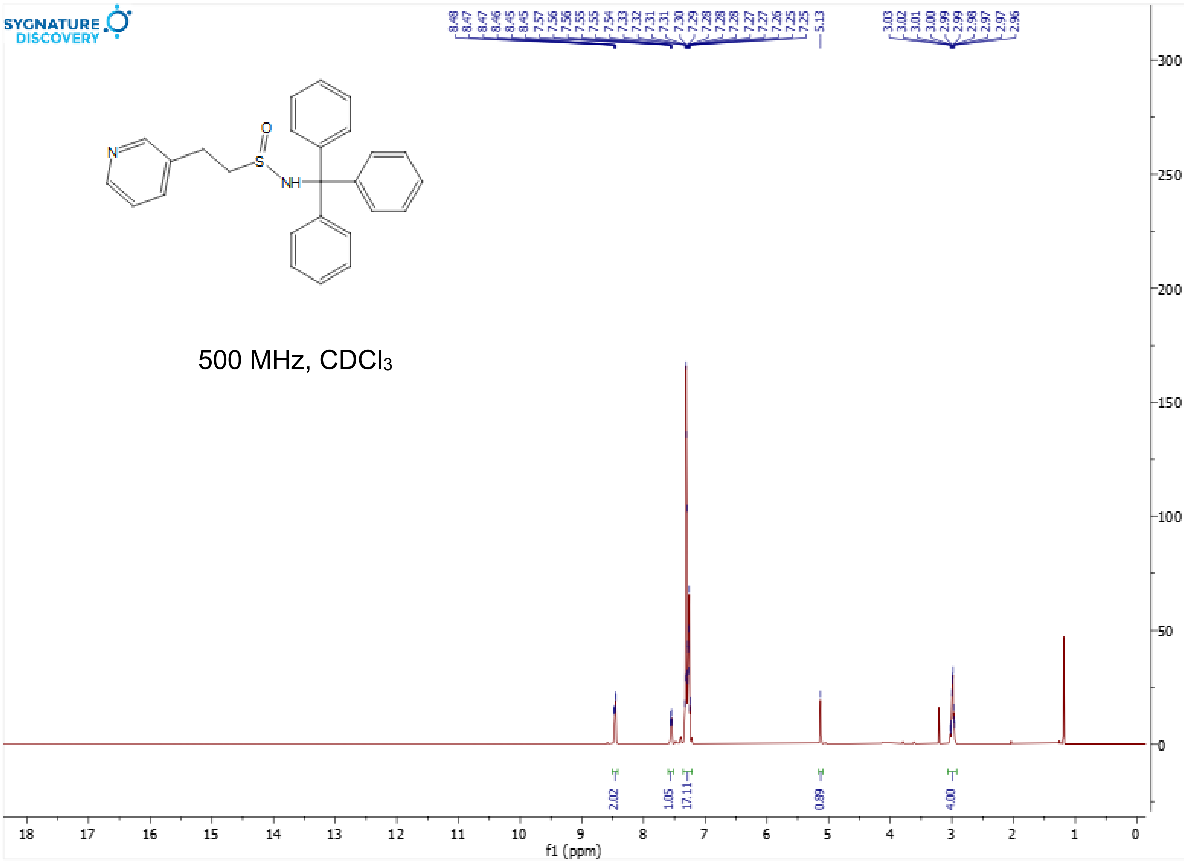

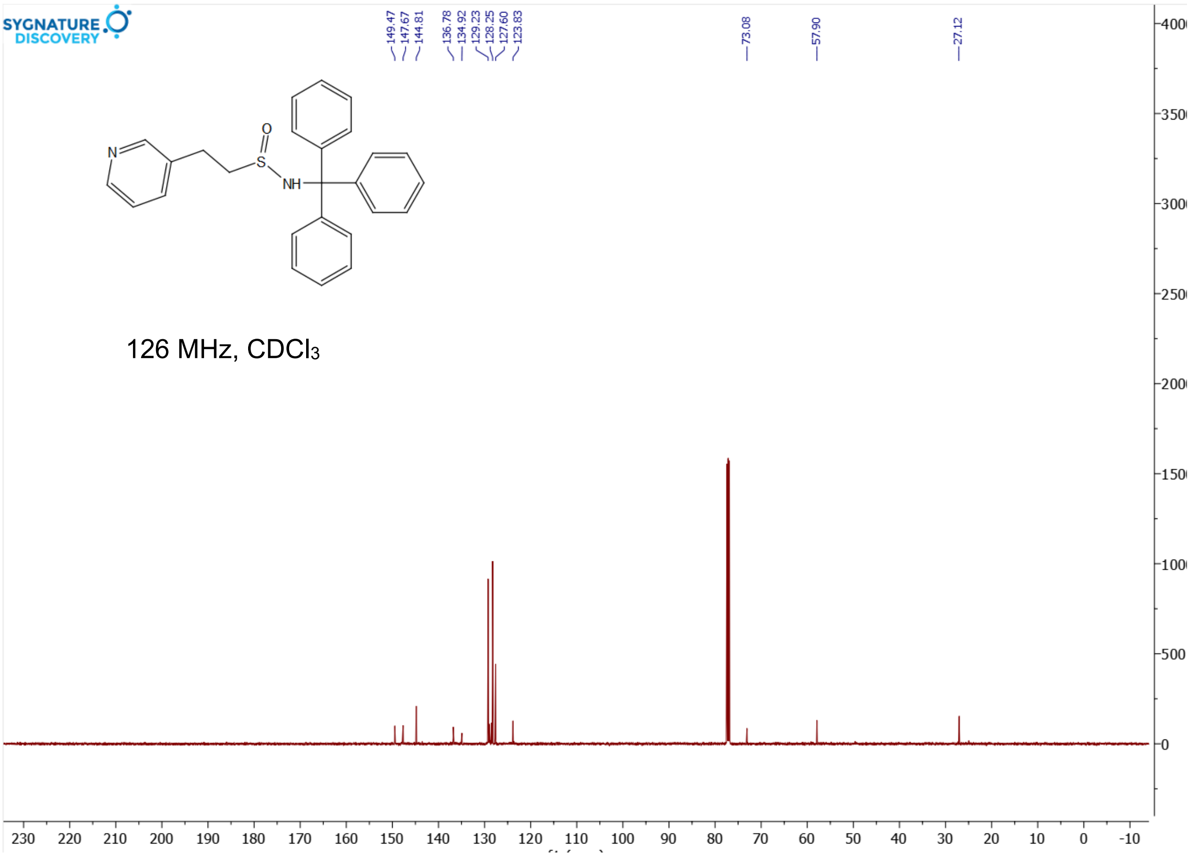

N-Tritylhexane-1-sulfinamide 1c

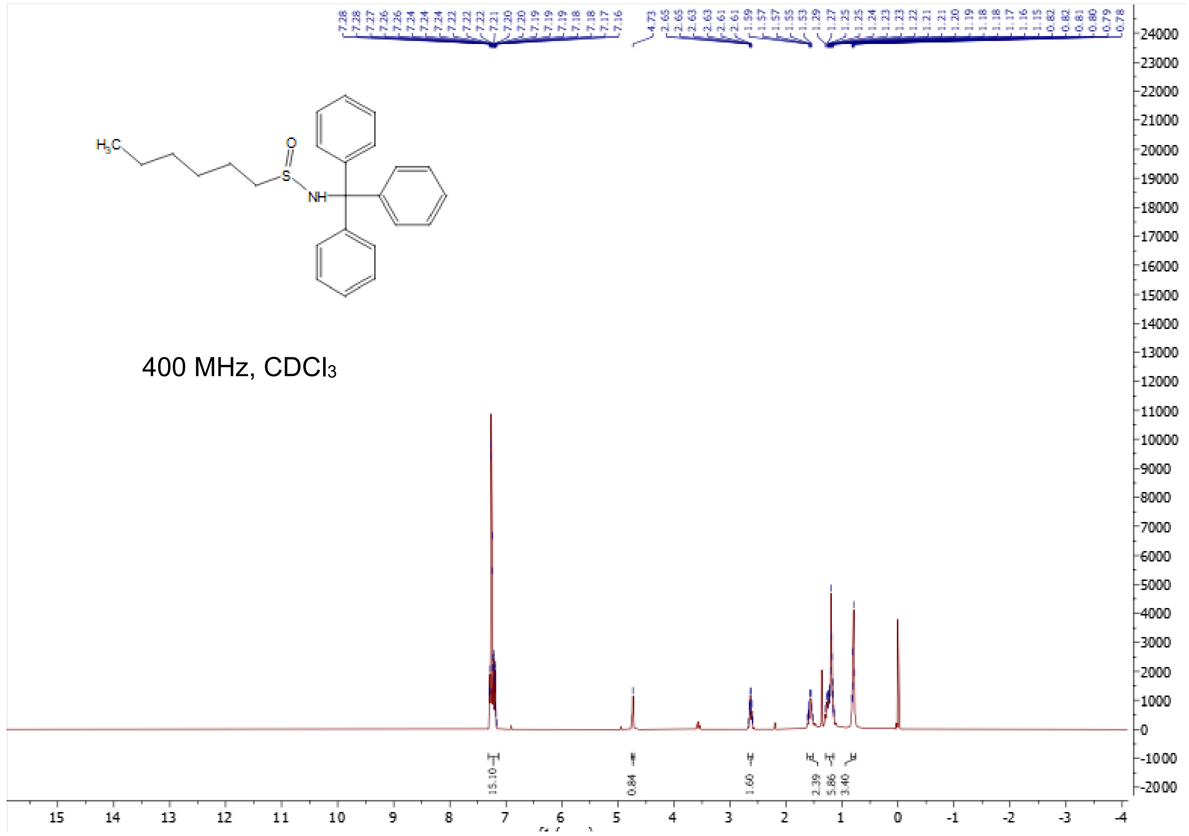

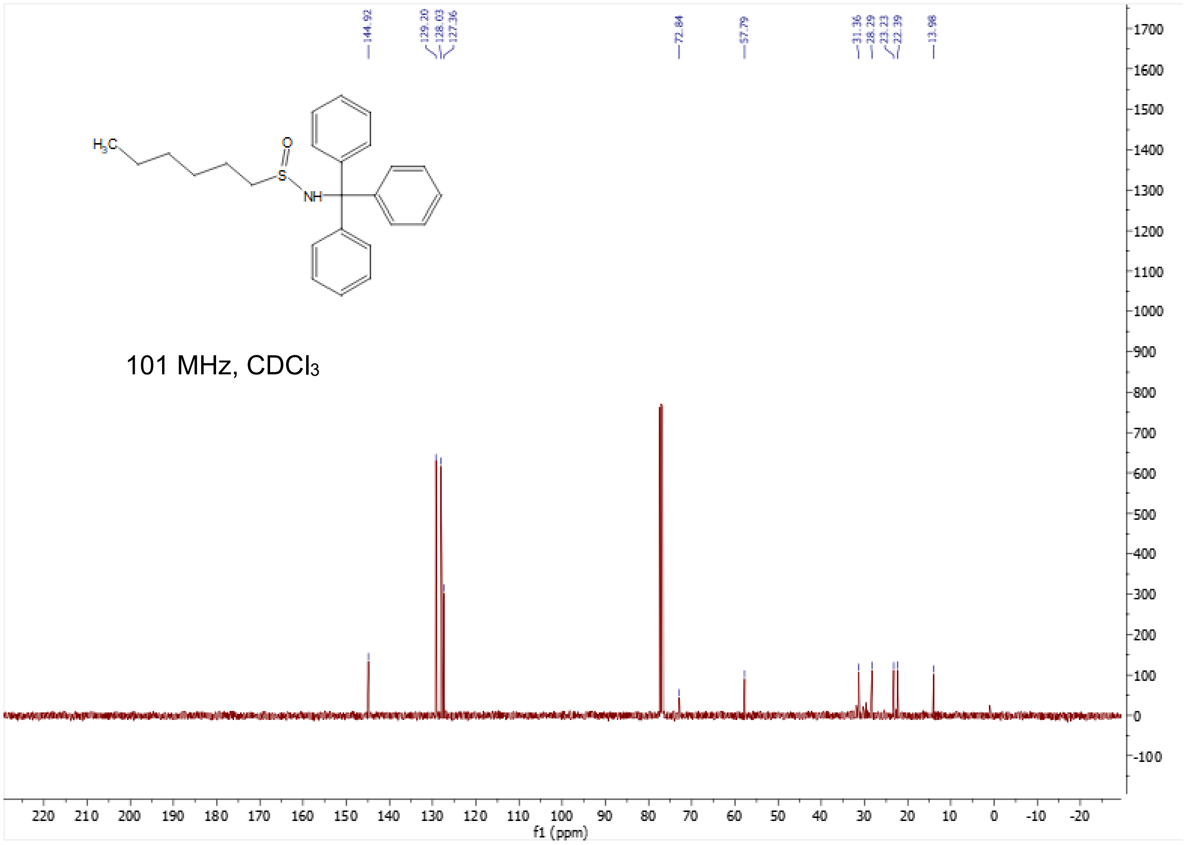

1-(4-fluorophenyl)-2-methyl-*N*-tritylpropane-2-sulfinamide 1d

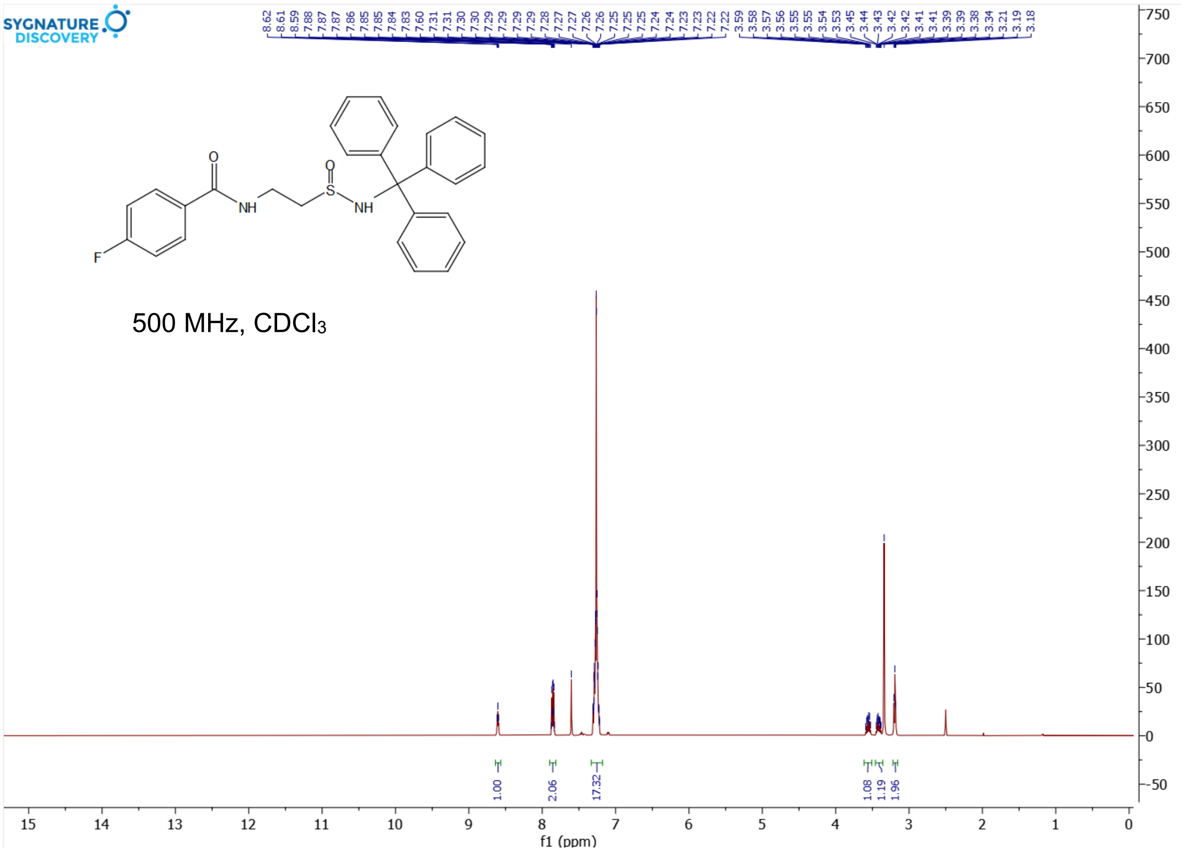

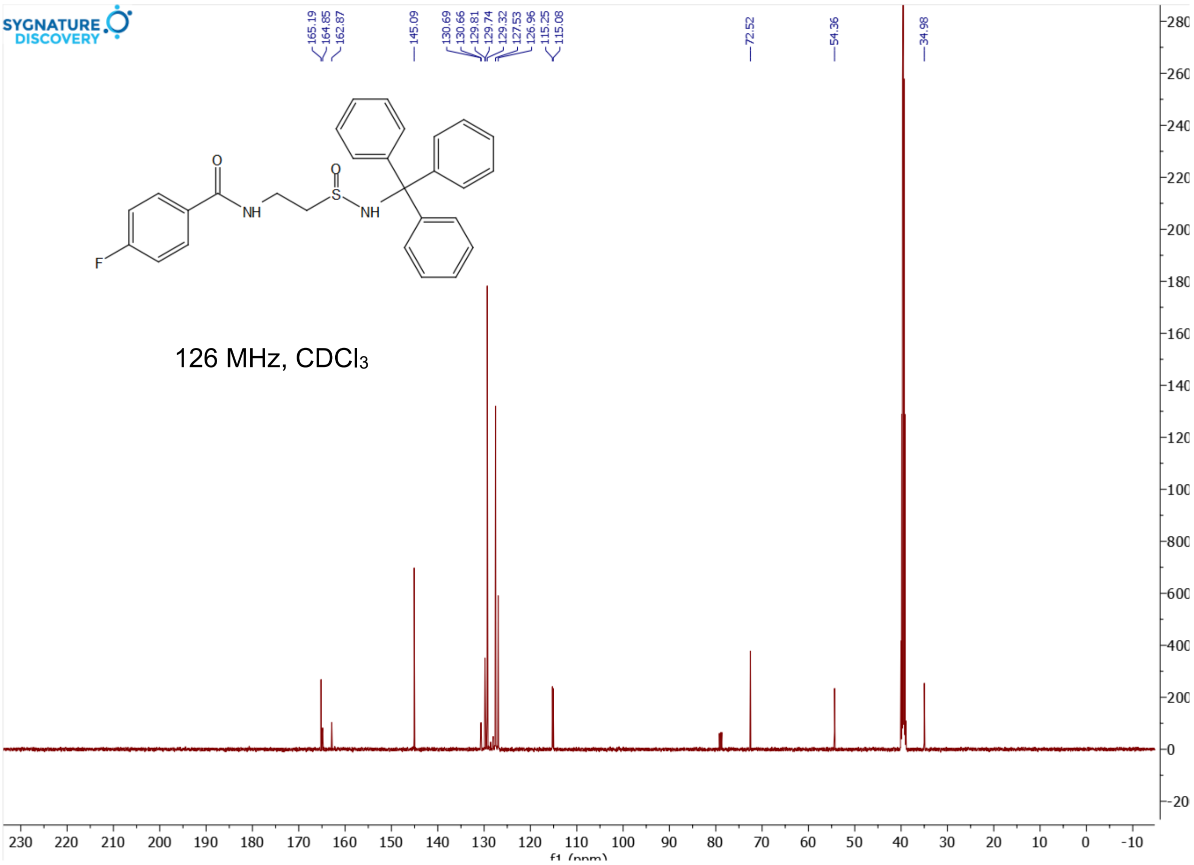

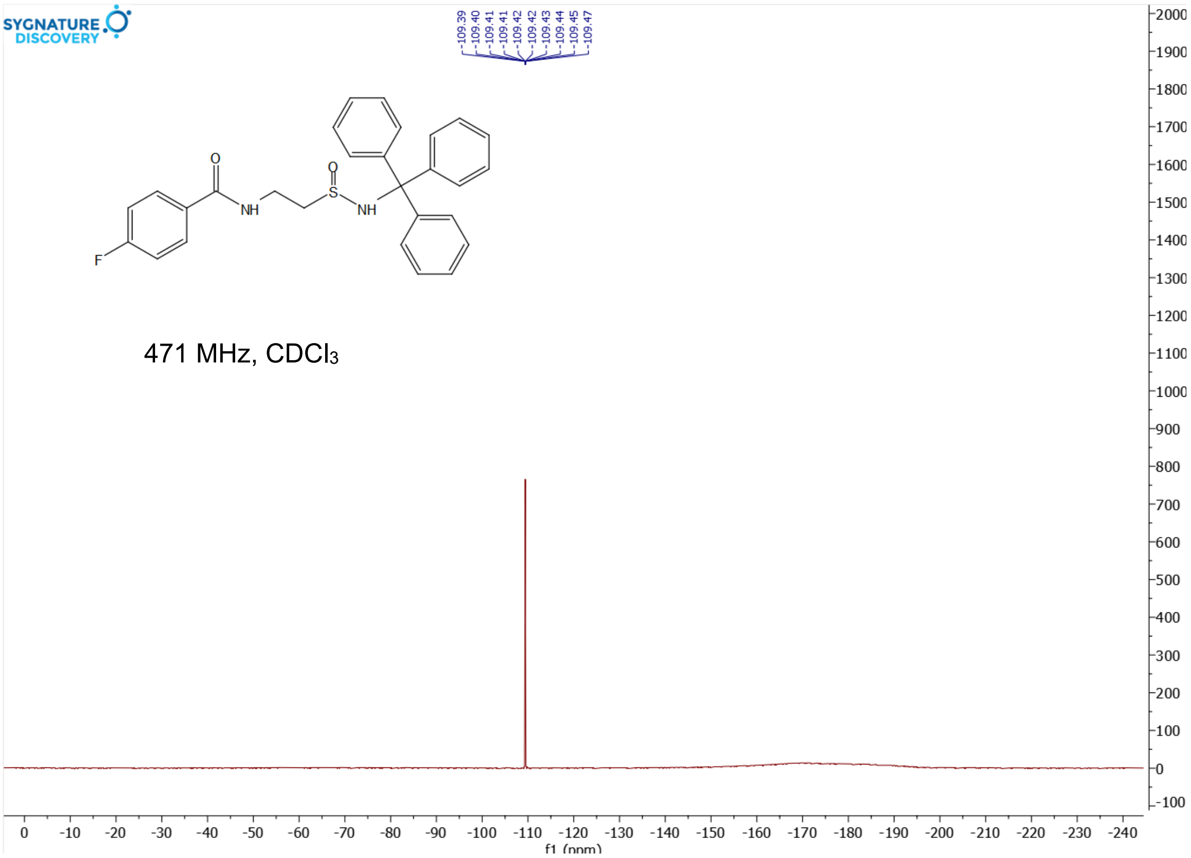

SYGNATURE DISCOVERY
-109.39
-109.40
-109.41
-109.41
-109.42
-109.42
-109.43
-109.44
-109.45
-109.47
471 MHz, CDCl3
f1 (ppm)

3-Methyl-*N*-tritylbutane-1-sulfinamide 1e

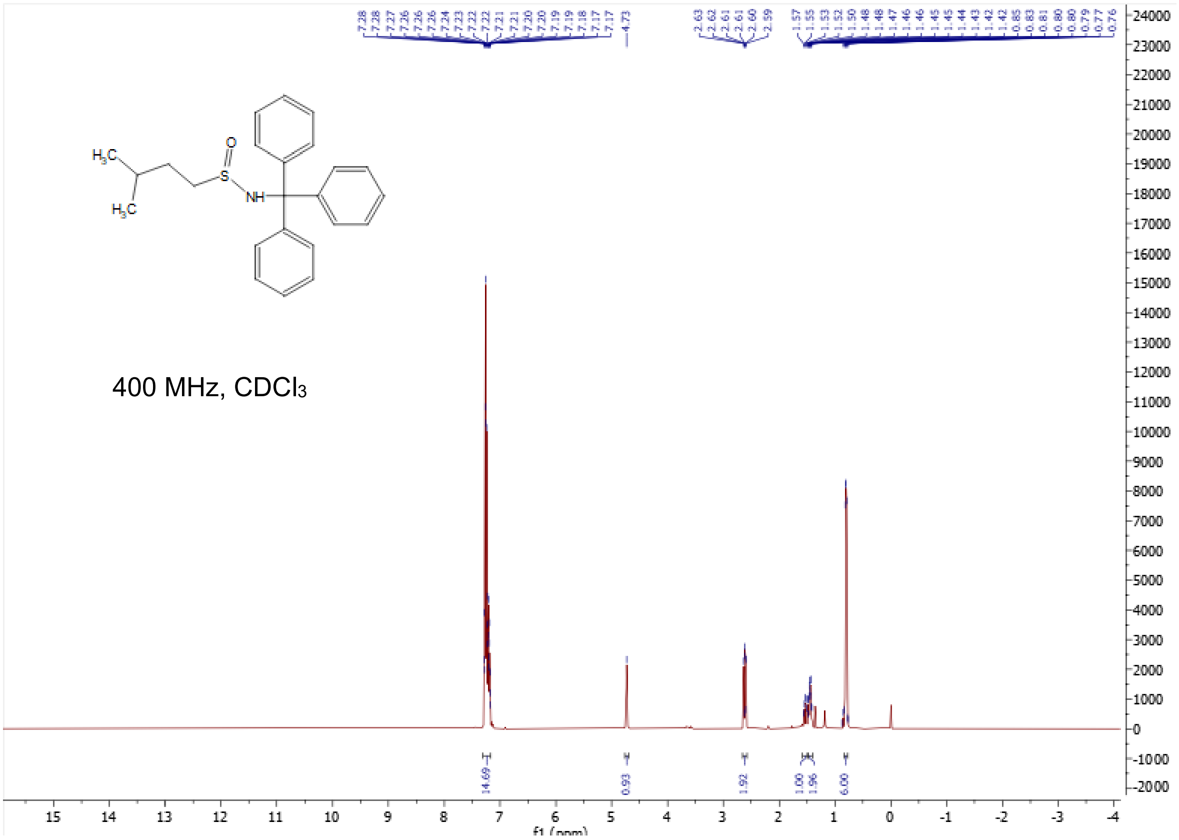

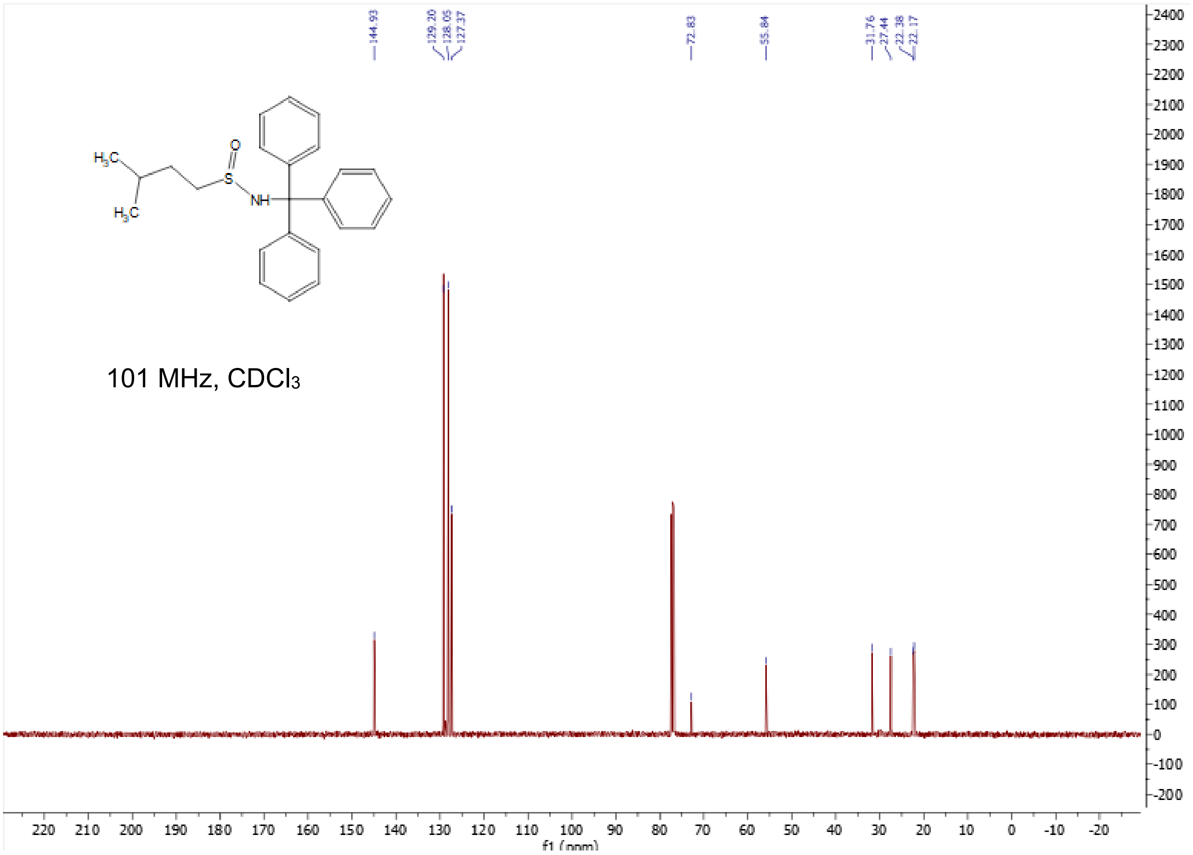

tert-Butyl (4-((tritylamino)sulfinyl)butyl)carbamate 1f

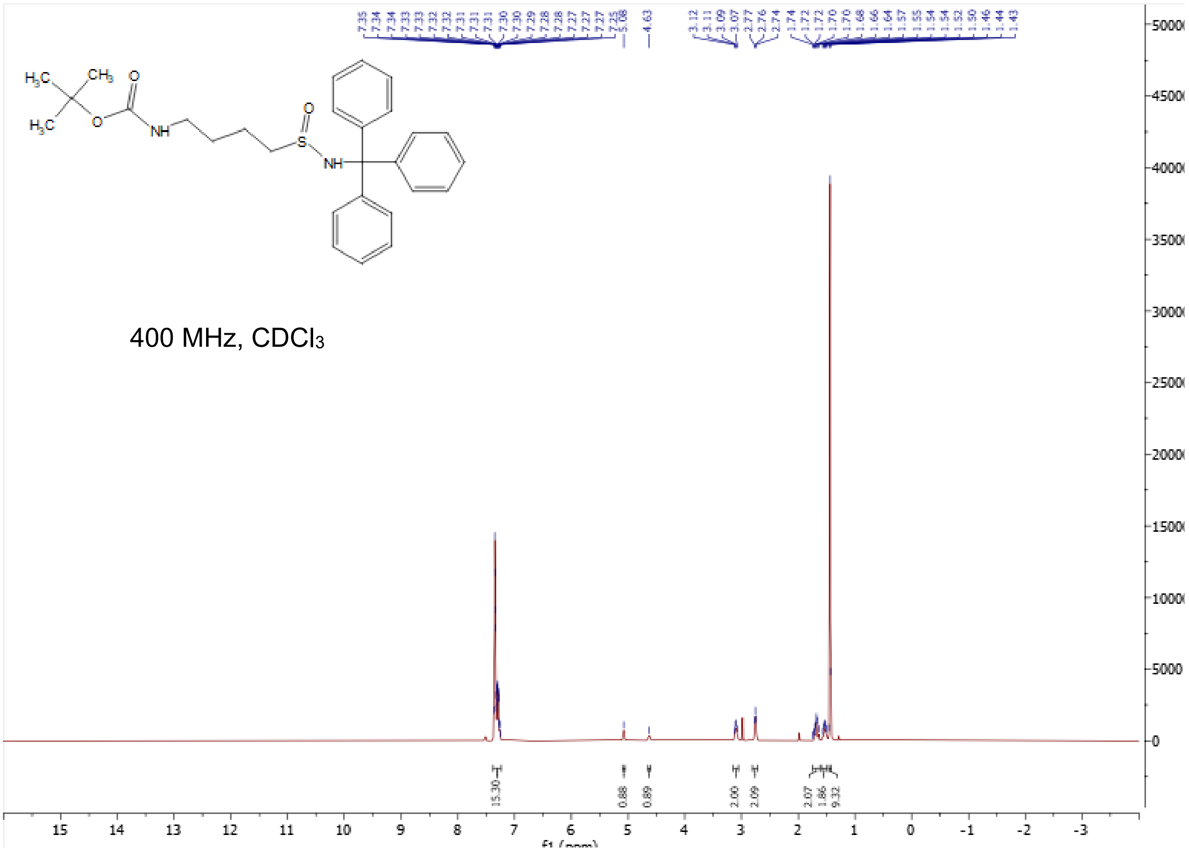

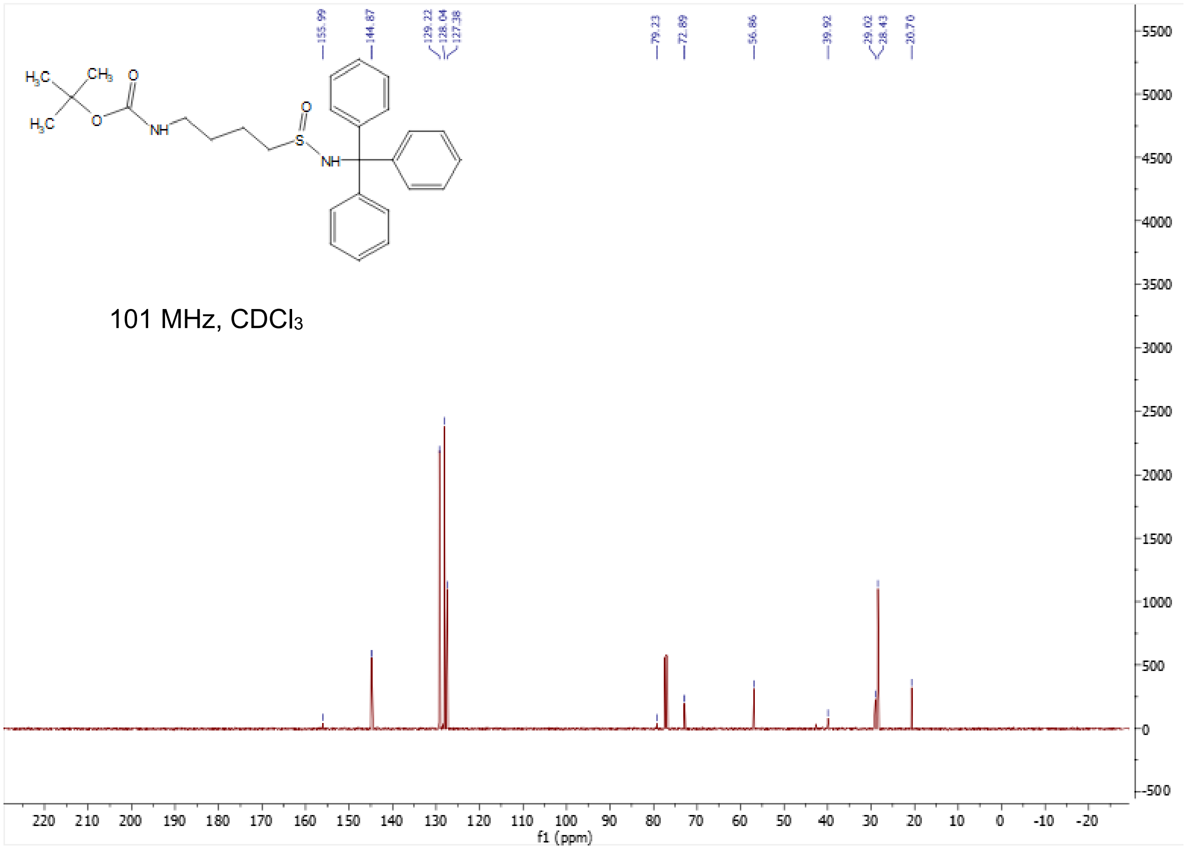

tert-Butyl (7-((tritylamino)sulfinyl)heptyl)carbamate 1g

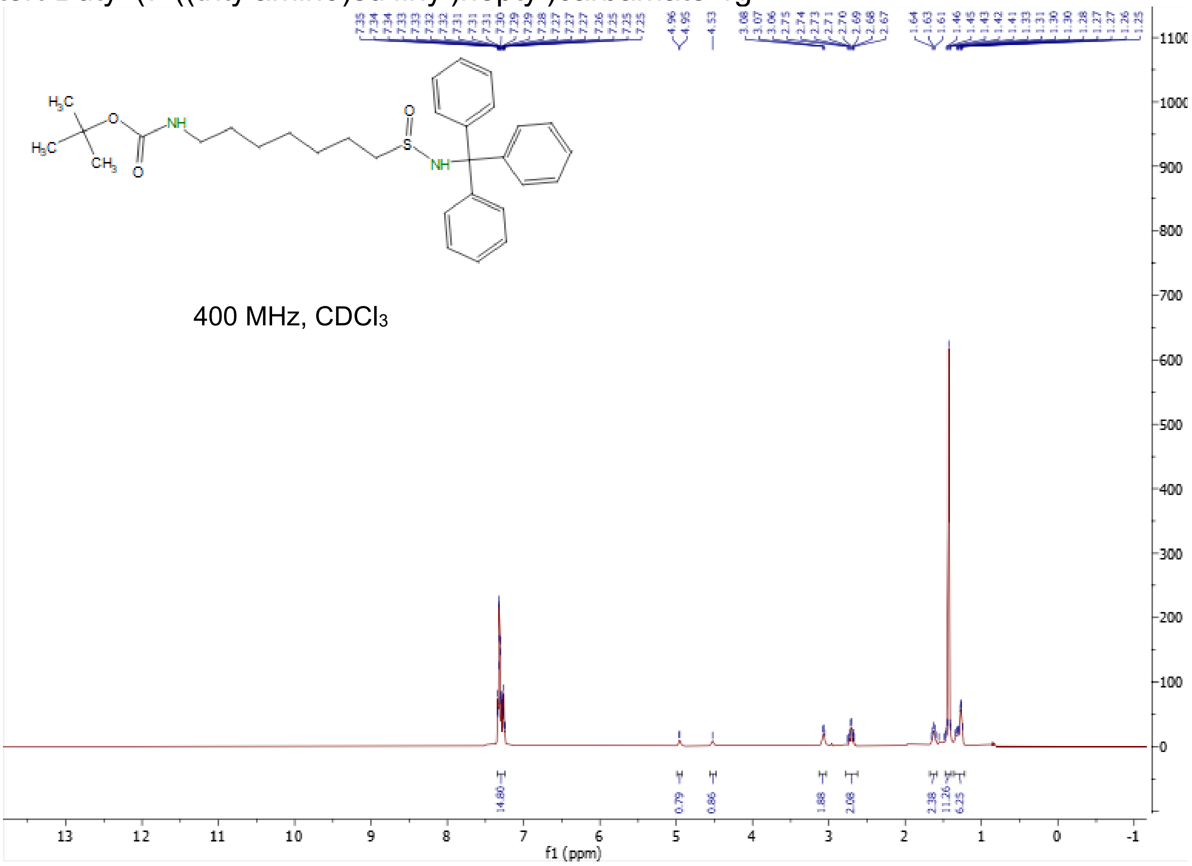

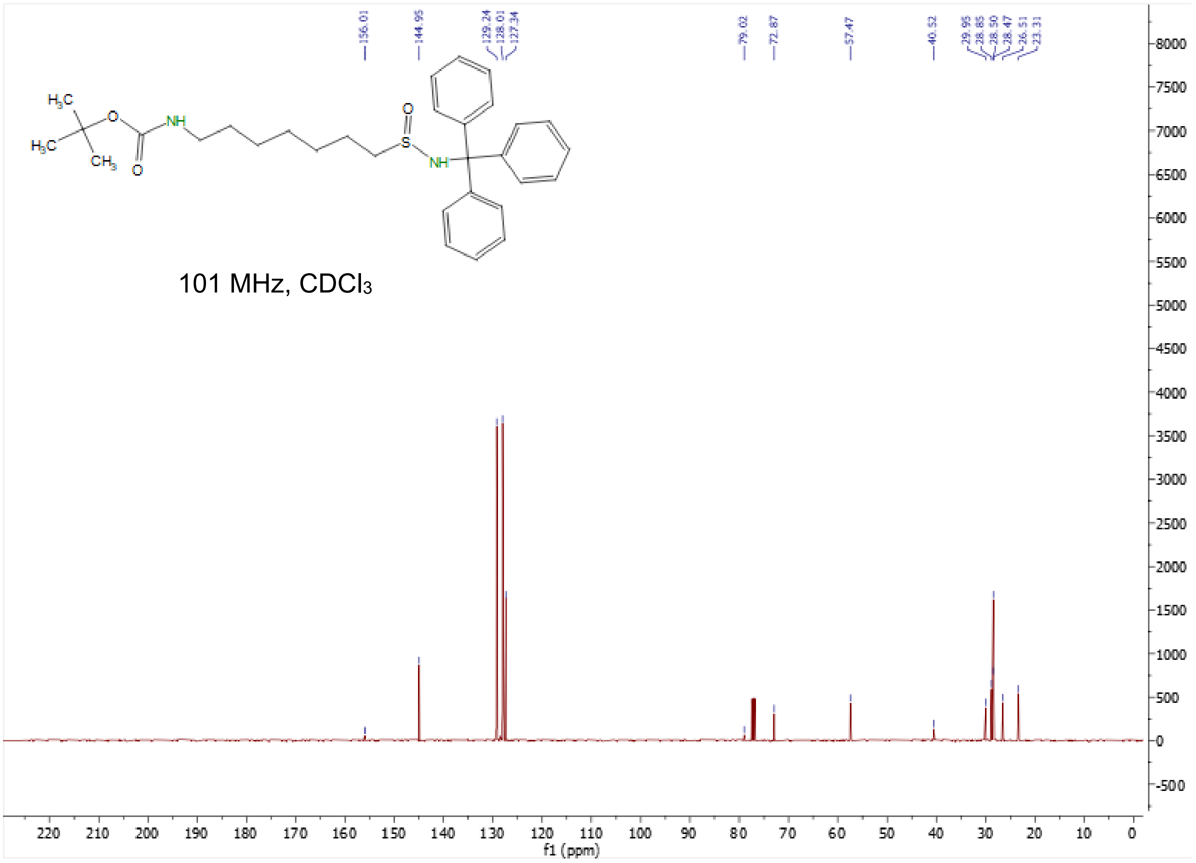

tert-Butyl (2-(2-((tritylamino)sulfinyl)ethoxy)ethyl)carbamate 1h

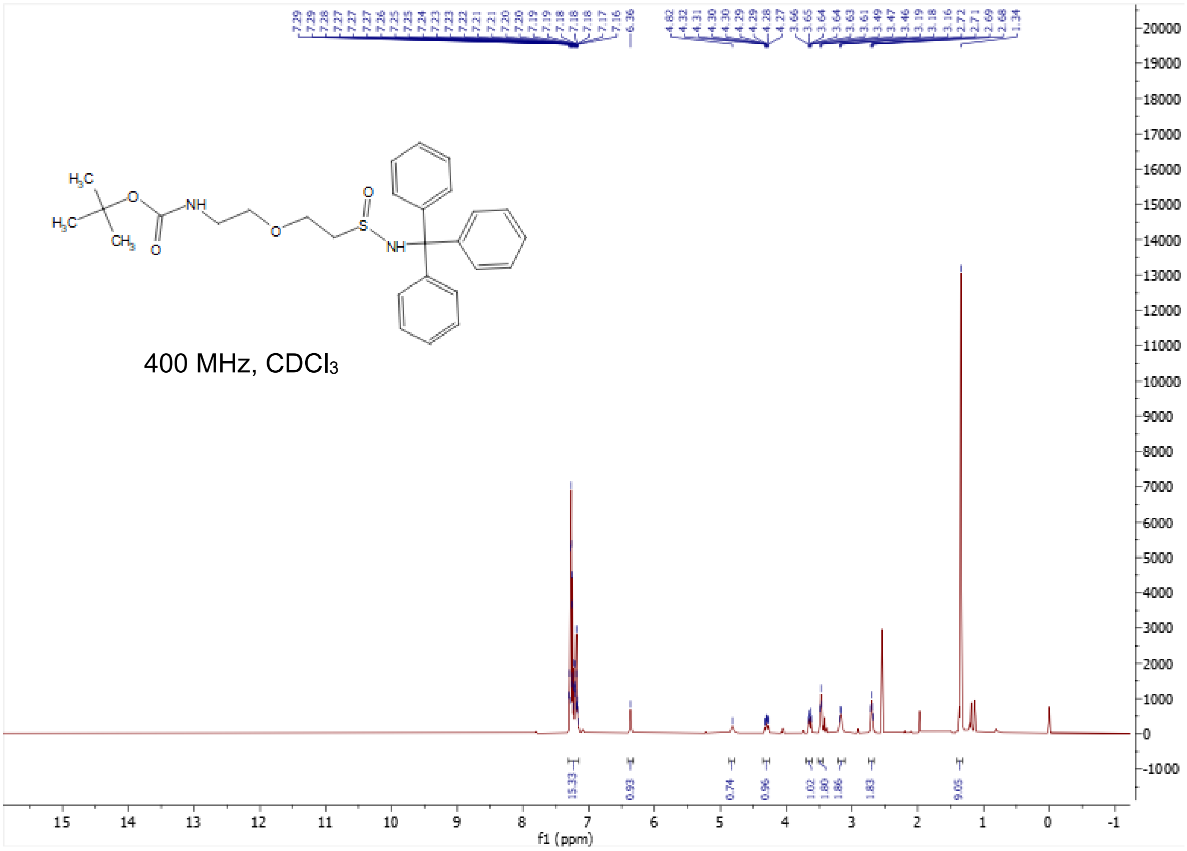

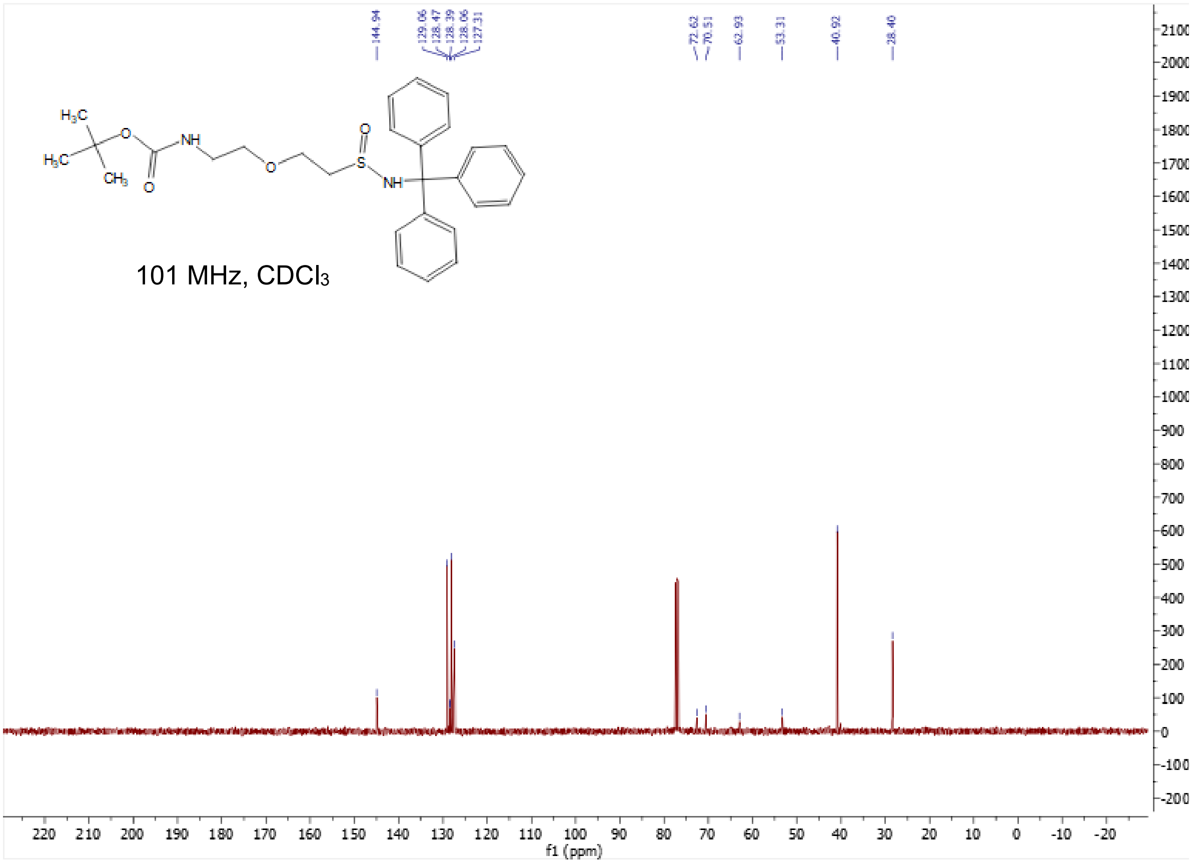

tert-butyl ((2S)-3-methyl-1-((tritylamino)sulfinyl)butan-2-yl)carbamate 1i

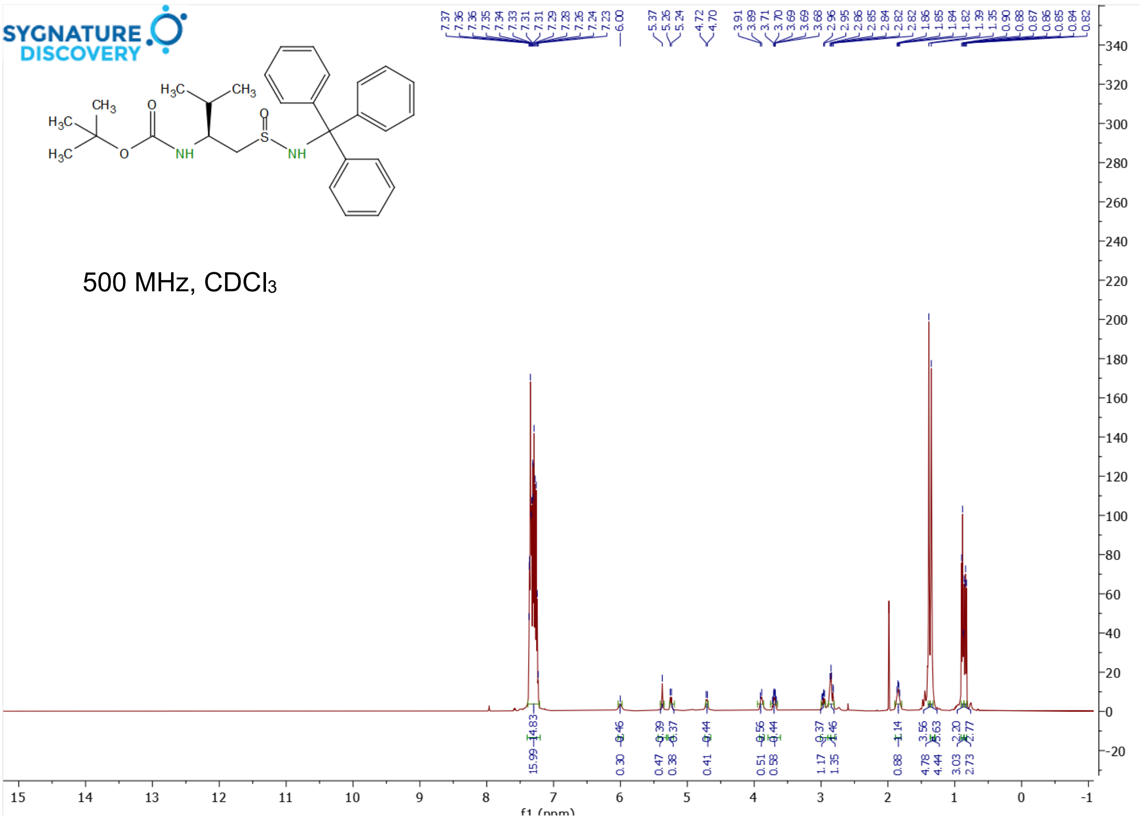

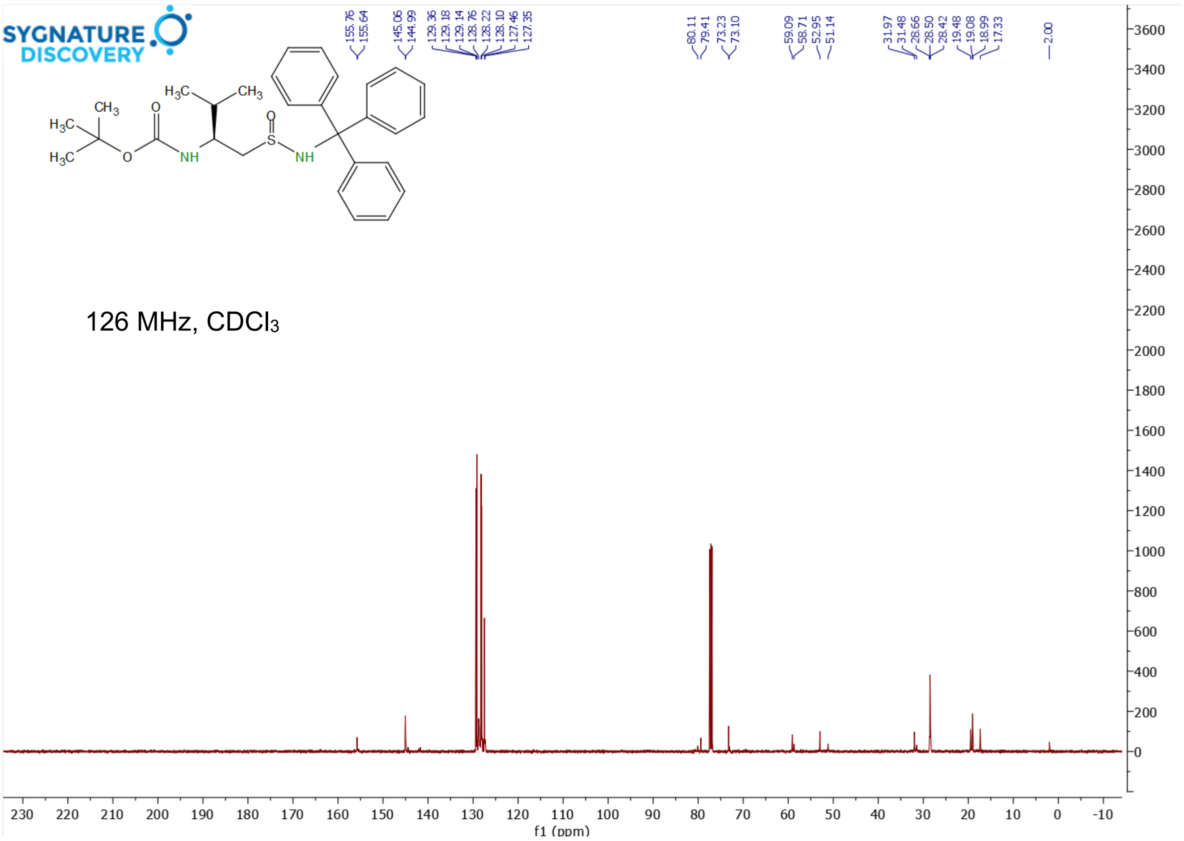

2-(benzo[d]thiazol-2-yl)-*N*-tritylethane-1-sulfinamide 1j

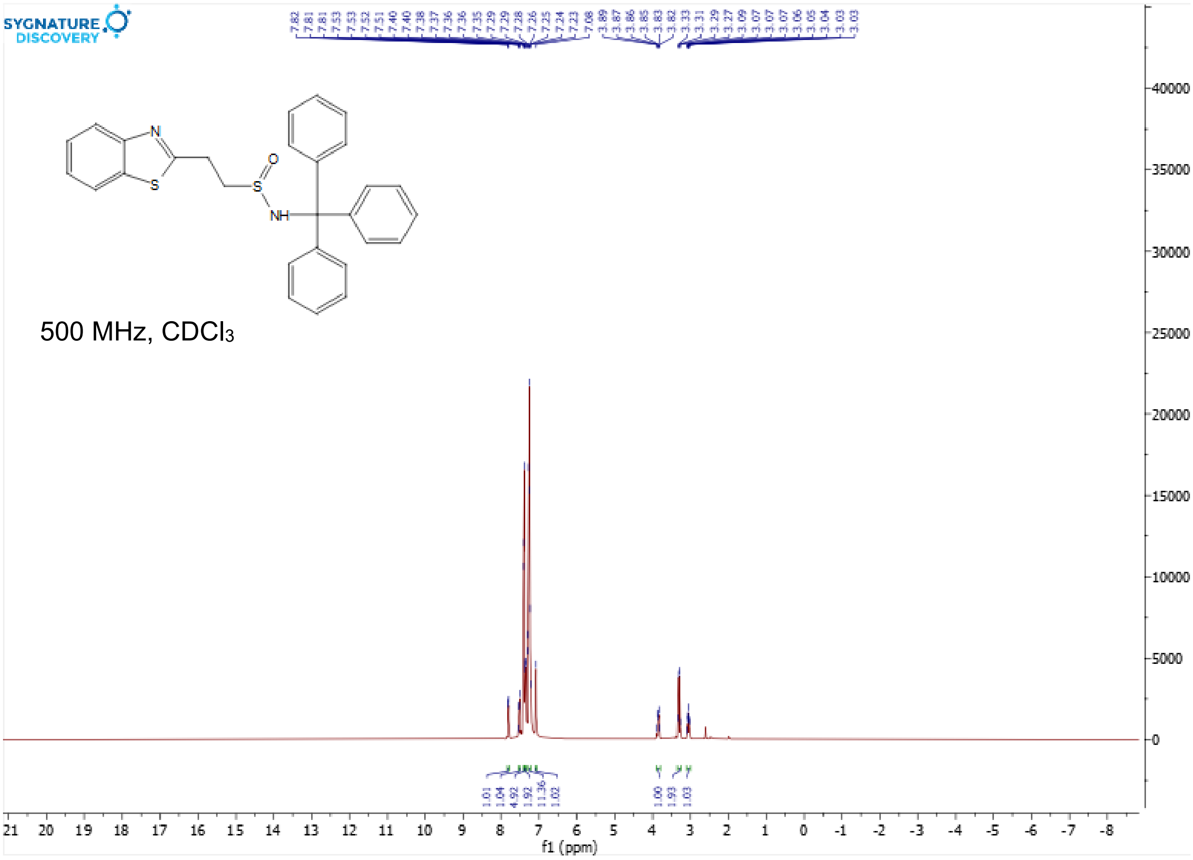

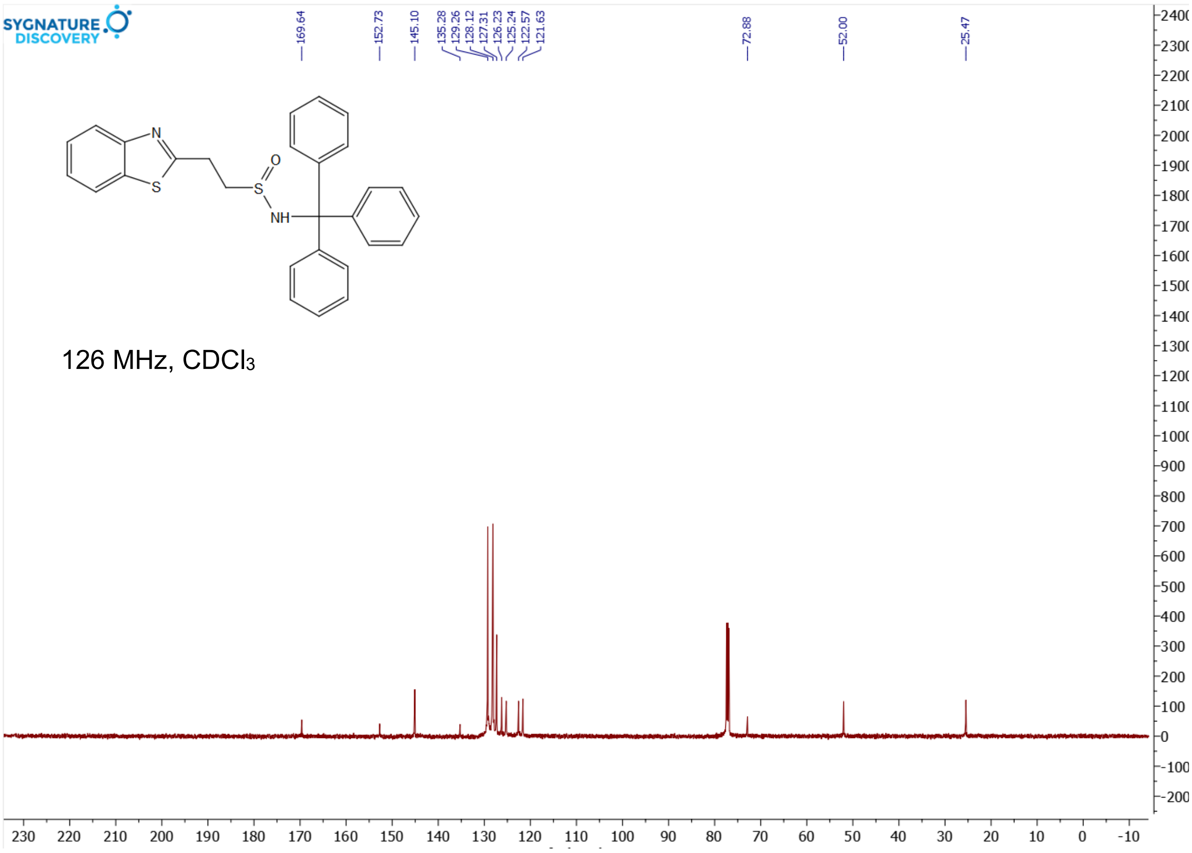

2-(5-phenyloxazol-2-yl)-*N*-tritylethane-1-sulfinamide 1k

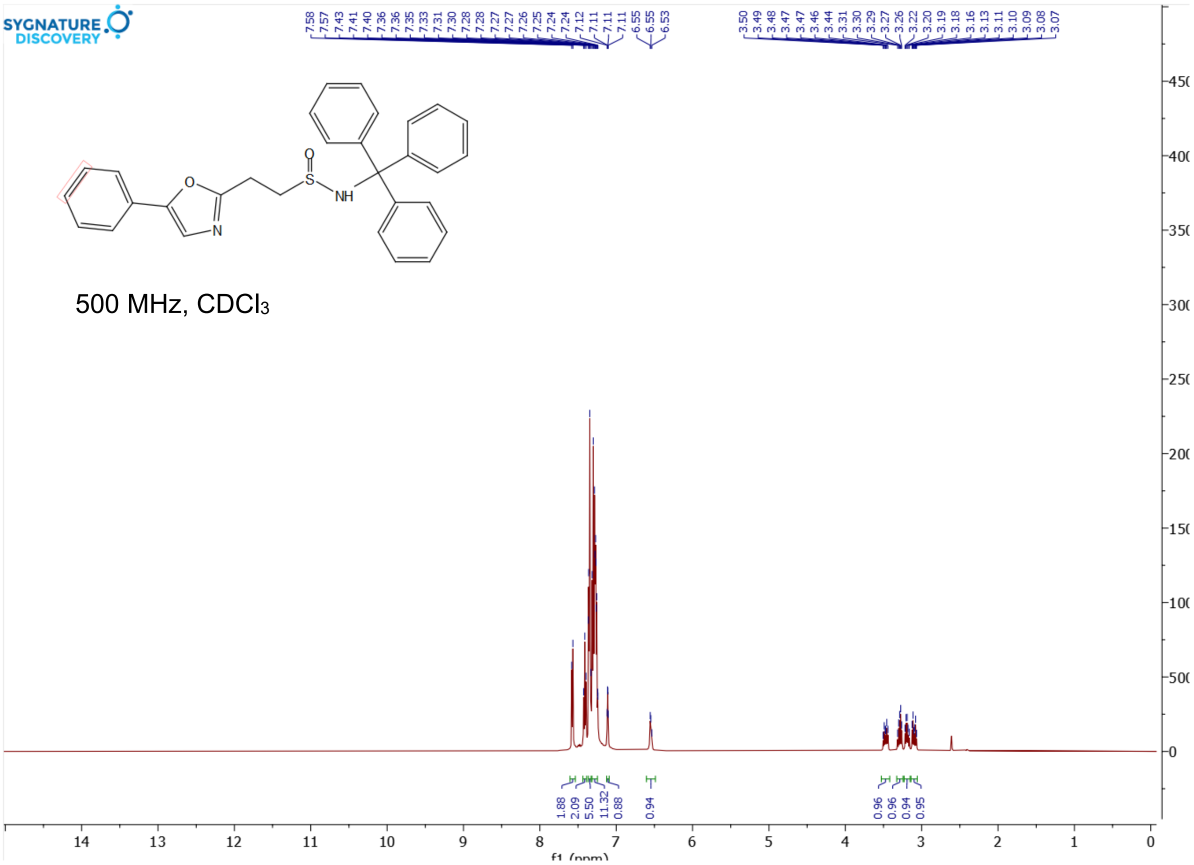

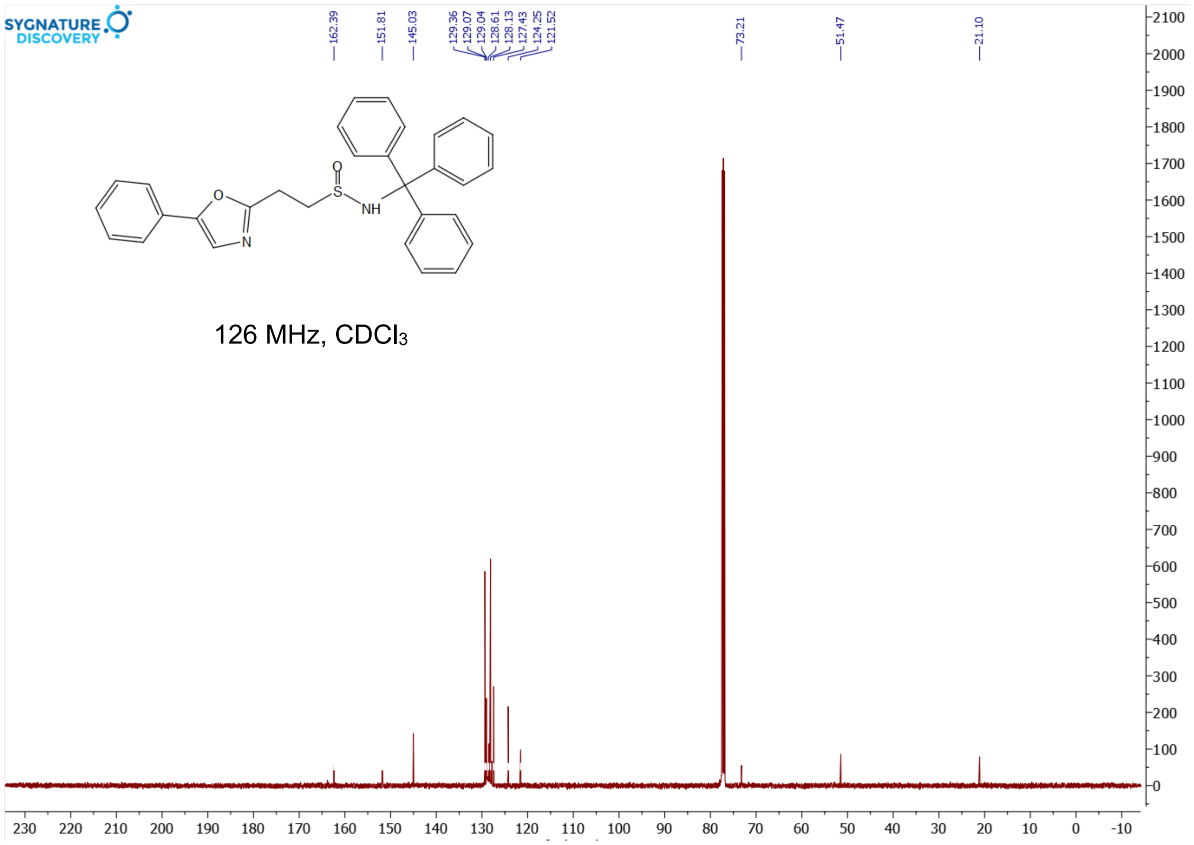

N-Tritylpropane-2-sulfinamide 1l

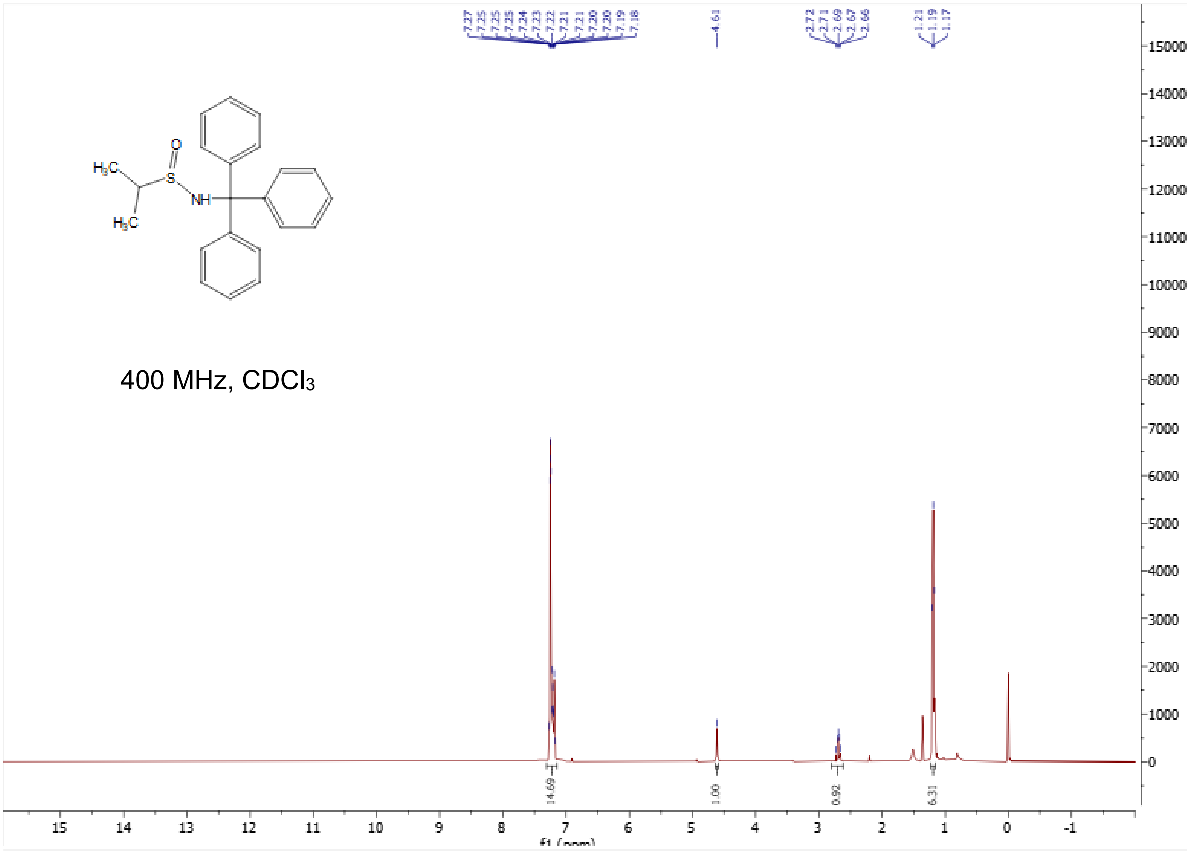

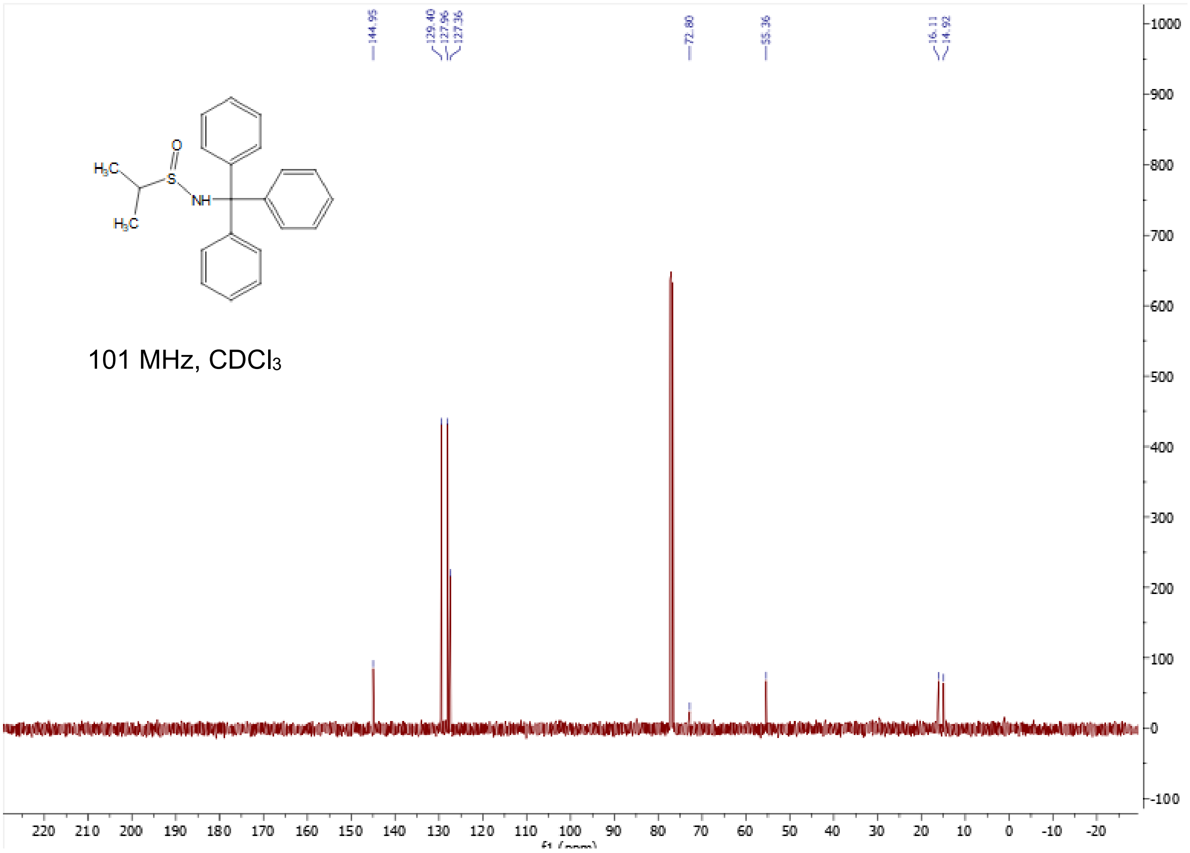

N-tritylcyclopentanesulfinamide 1m

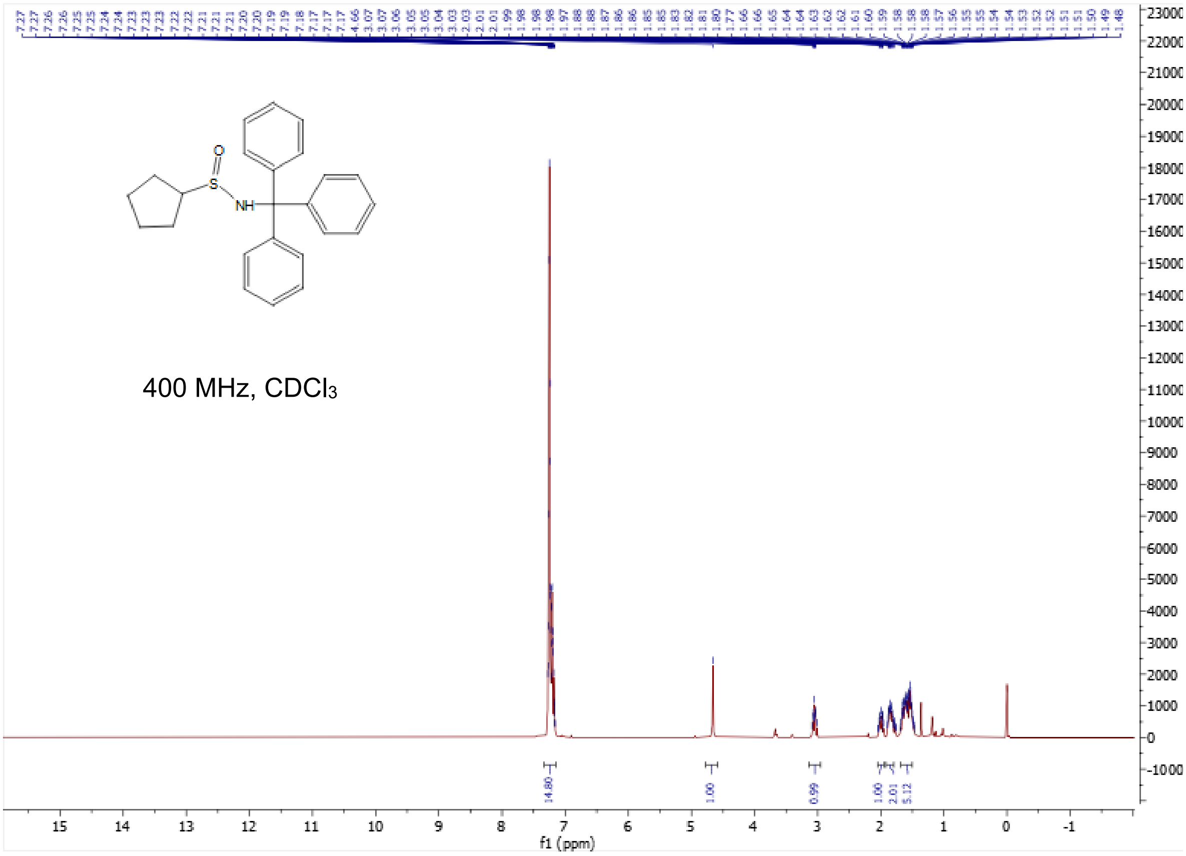

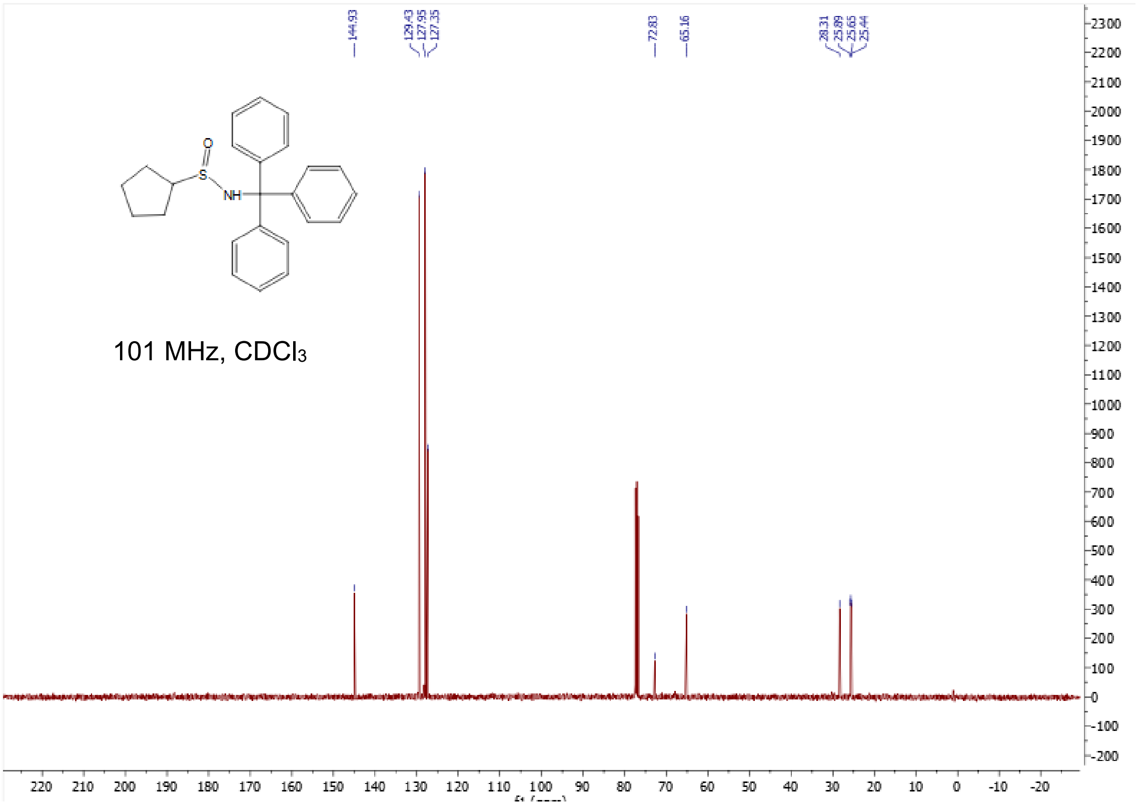

N-Tritylcyclohexanesulfinamide 1n

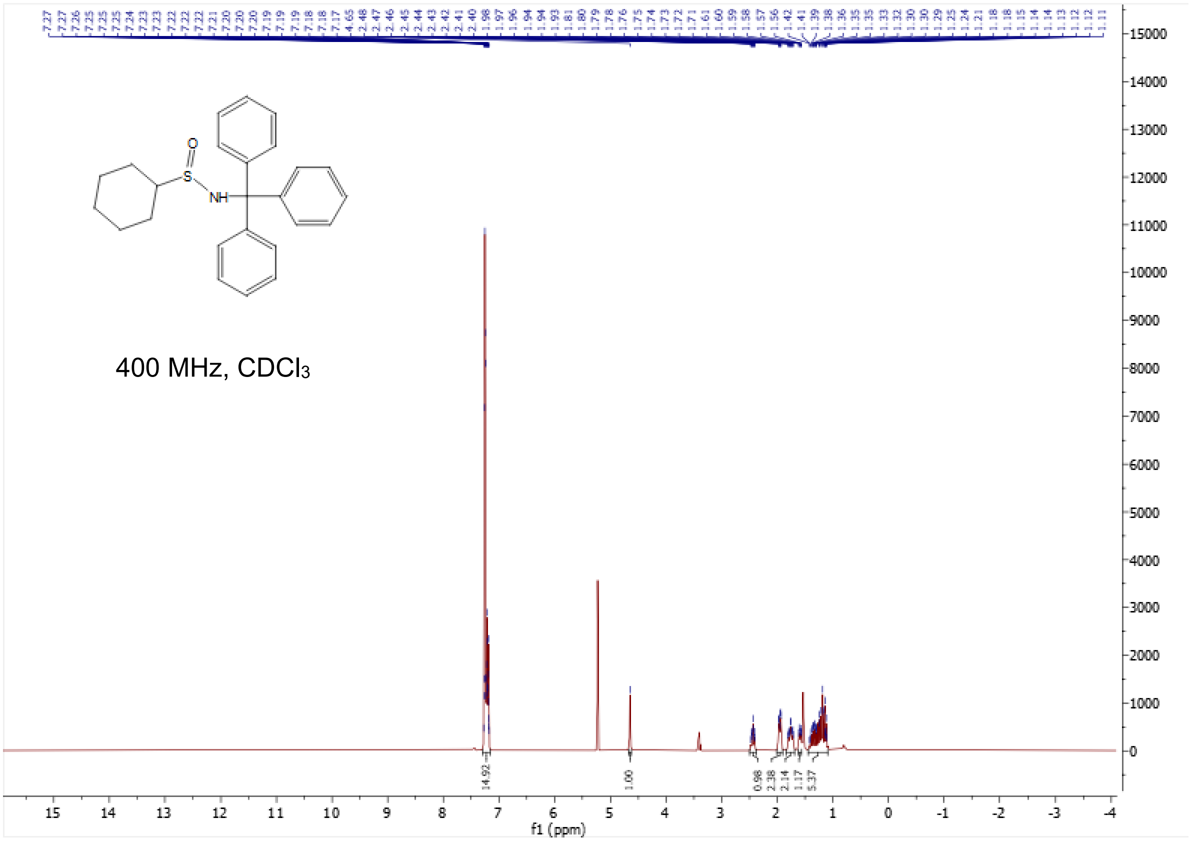

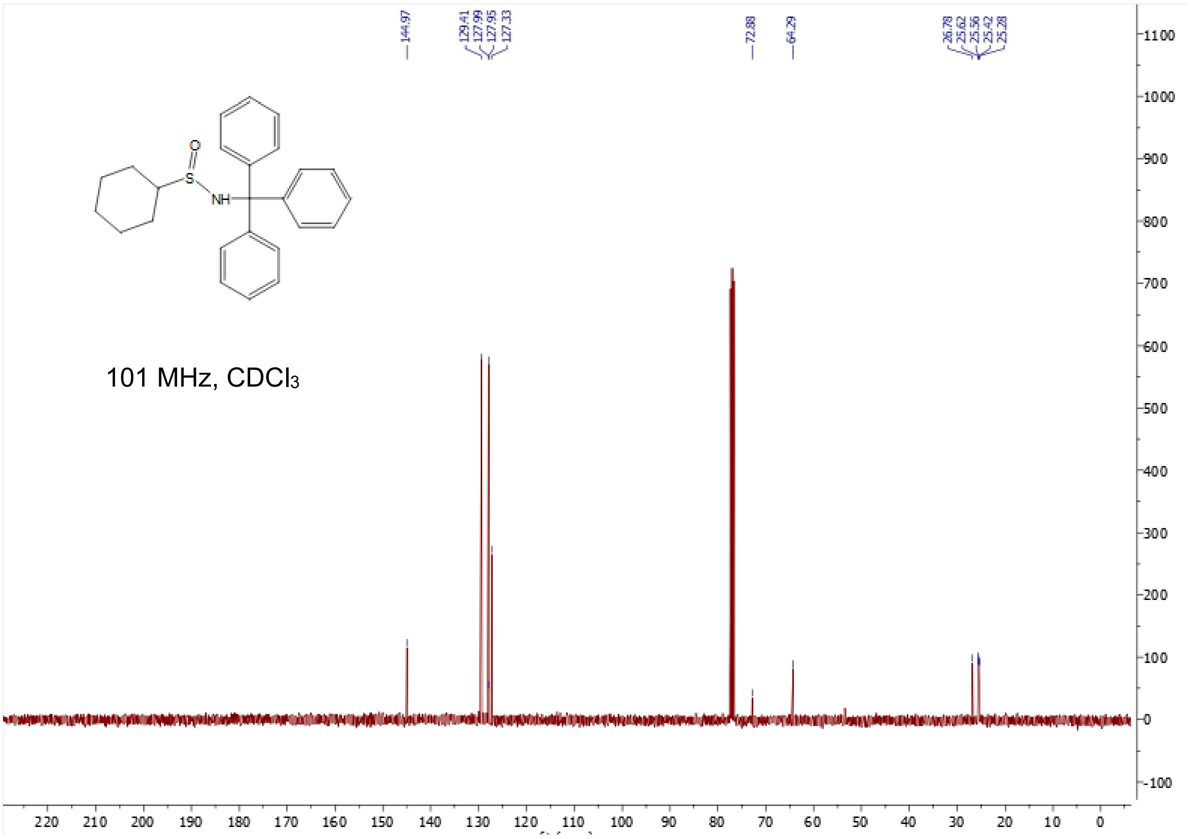

N-trityltetrahydro-2H-pyran-4-sulfinamide 1o

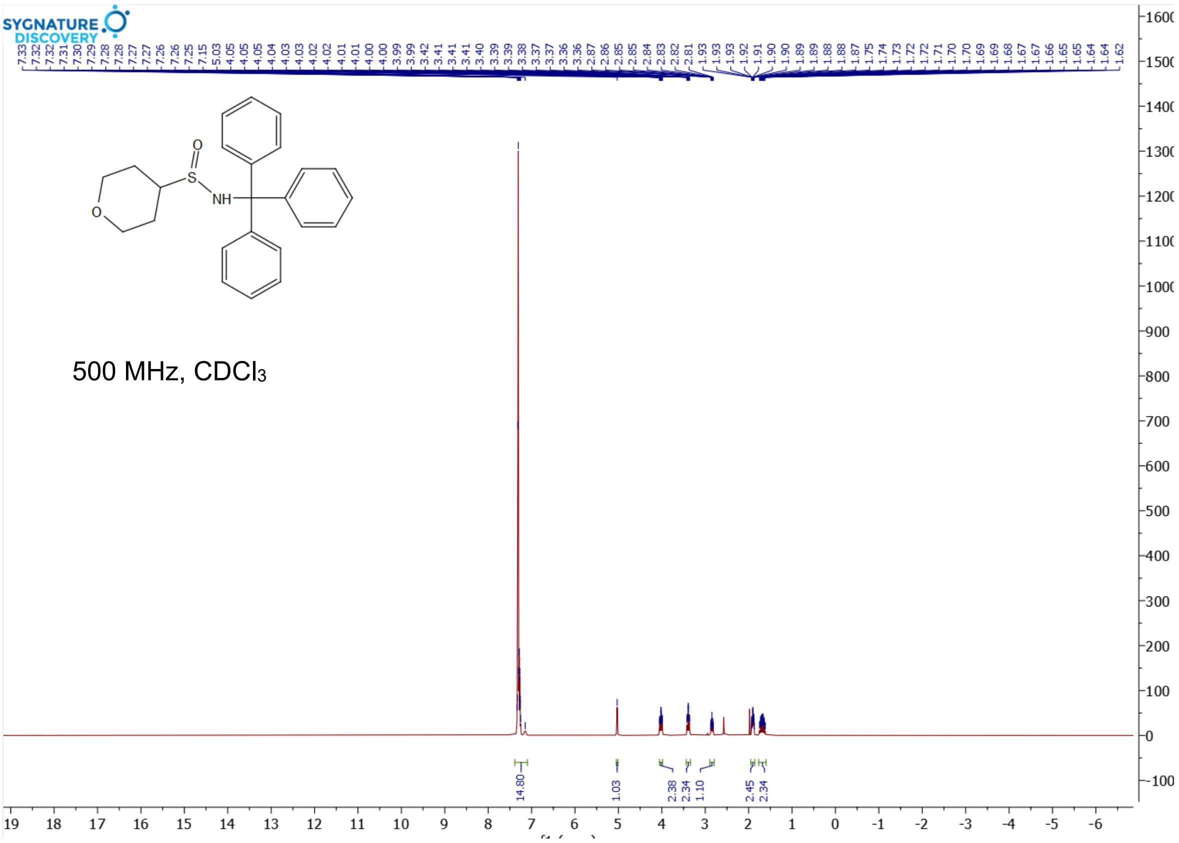

500 MHz, $CDCl_3$

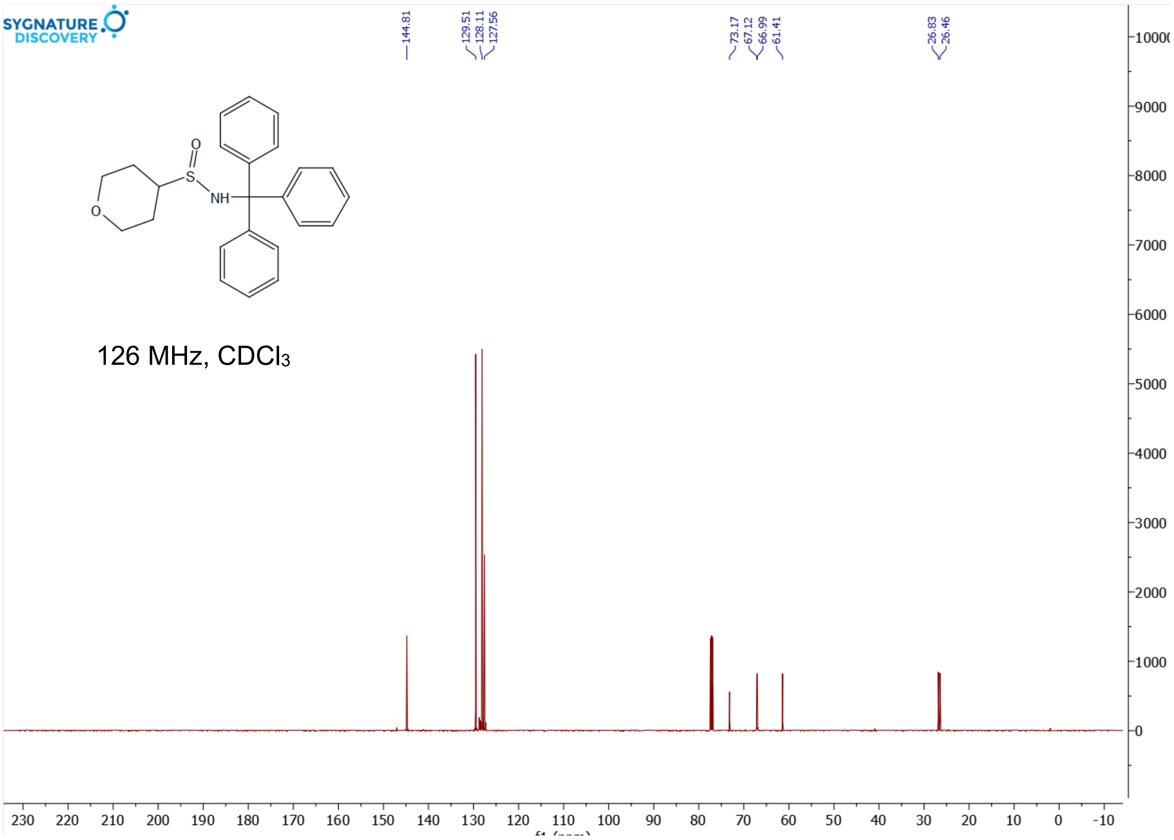

126 MHz, $CDCl_3$

1-tosyl-*N*-tritylpiperidine-4-sulfinamide 1p

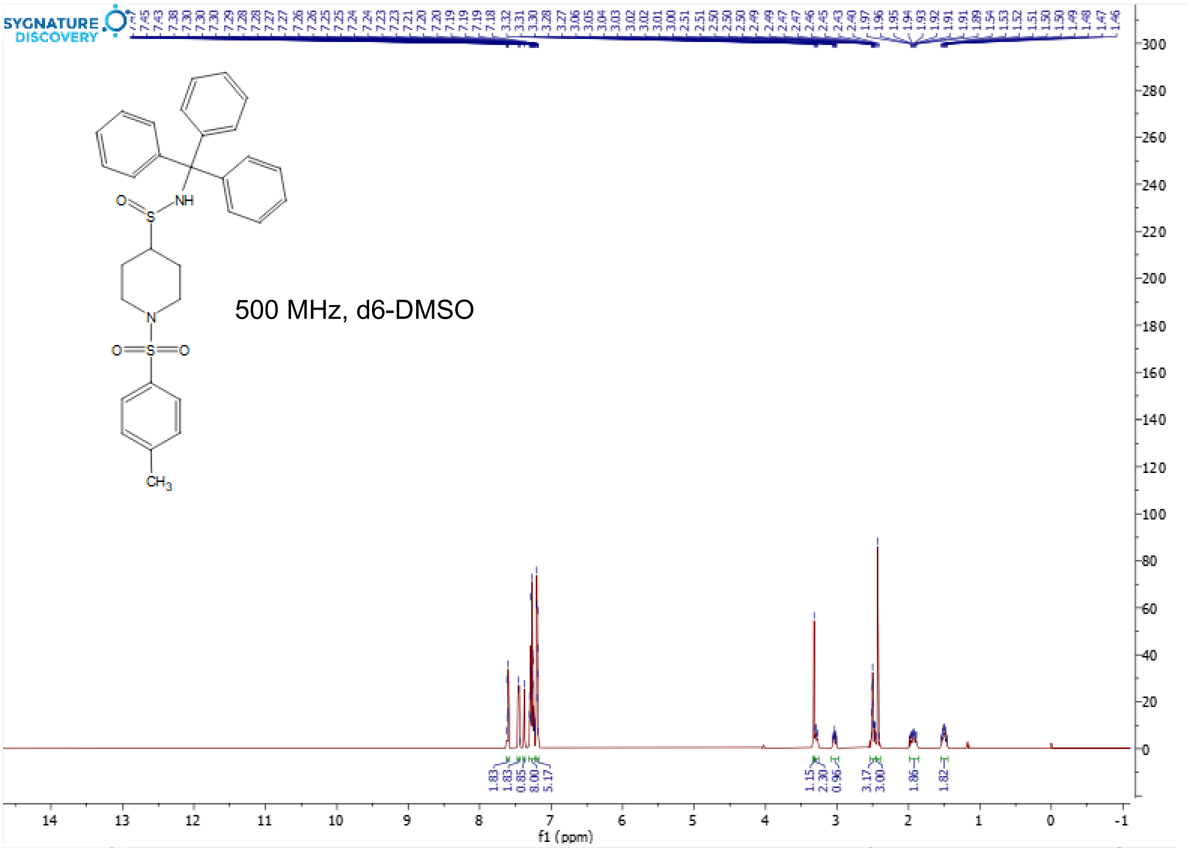

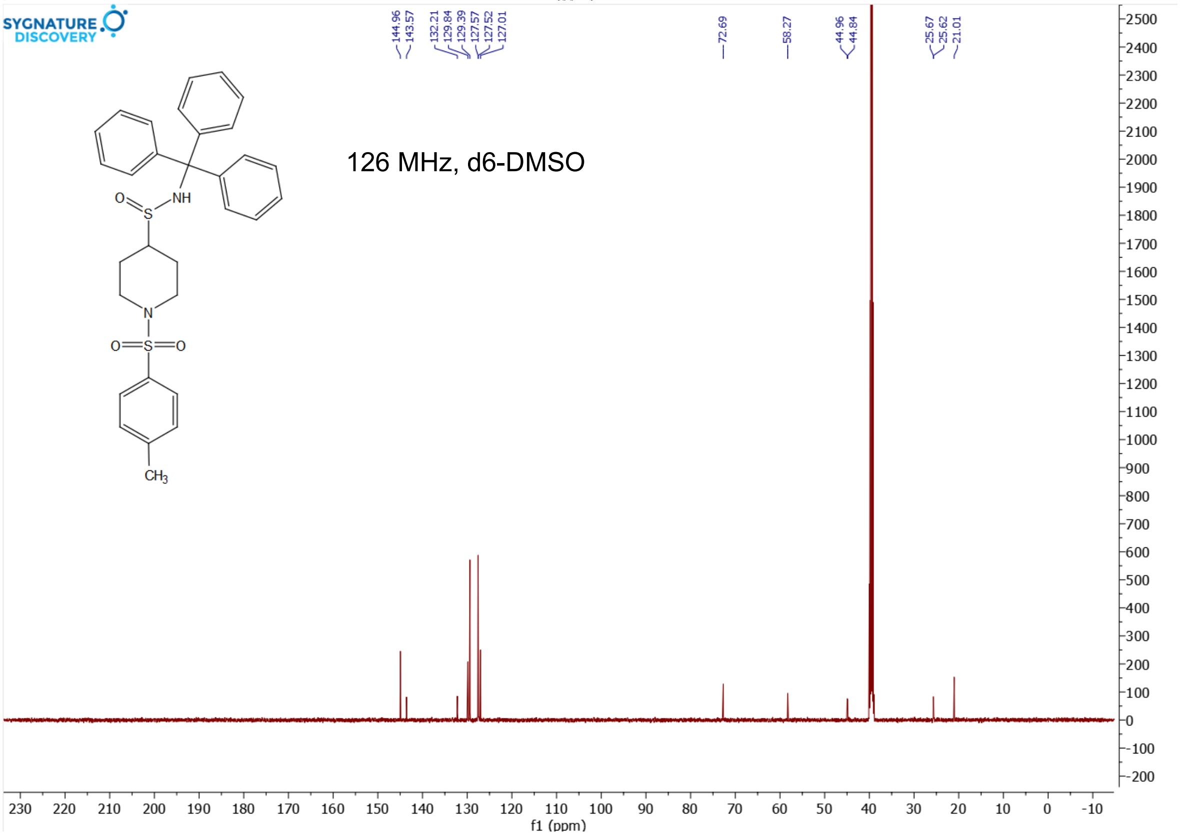

1-(3-methoxybenzoyl)-*N*-tritylpiperidine-4-sulfinamide 1q

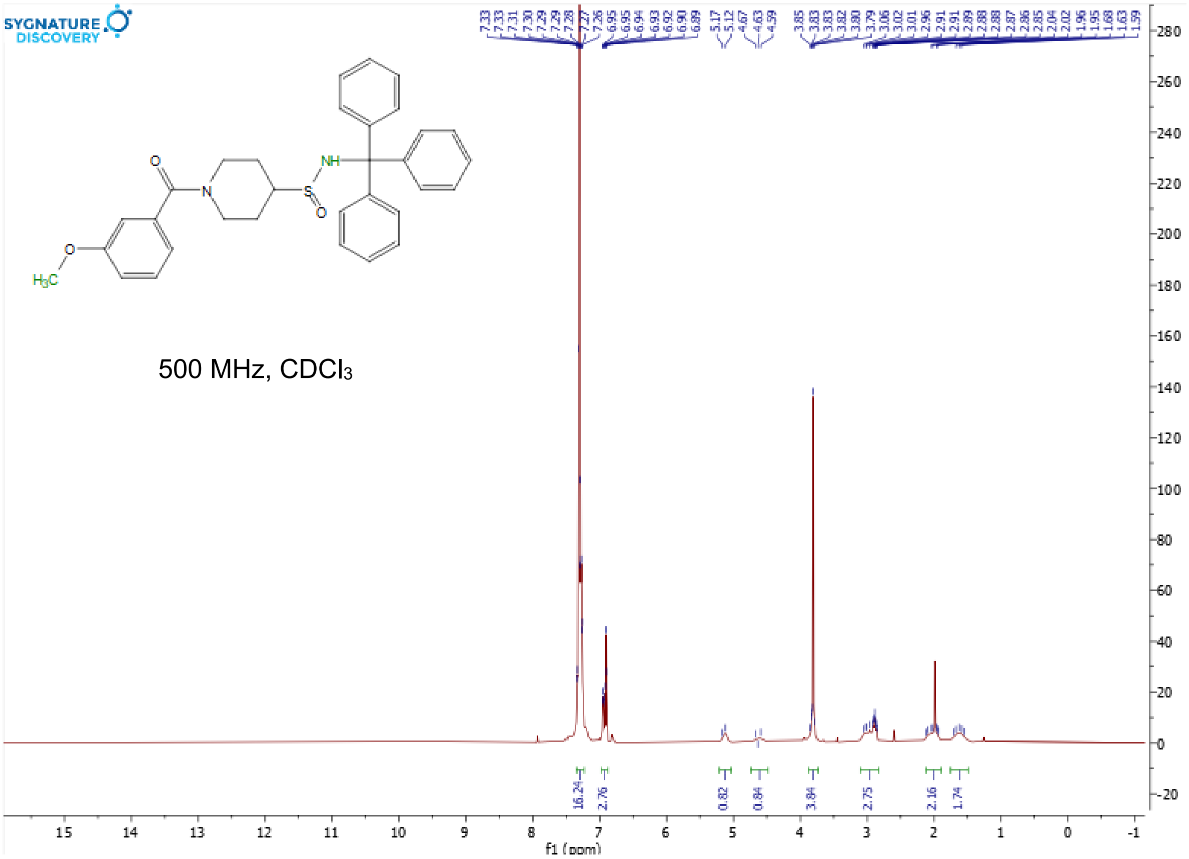

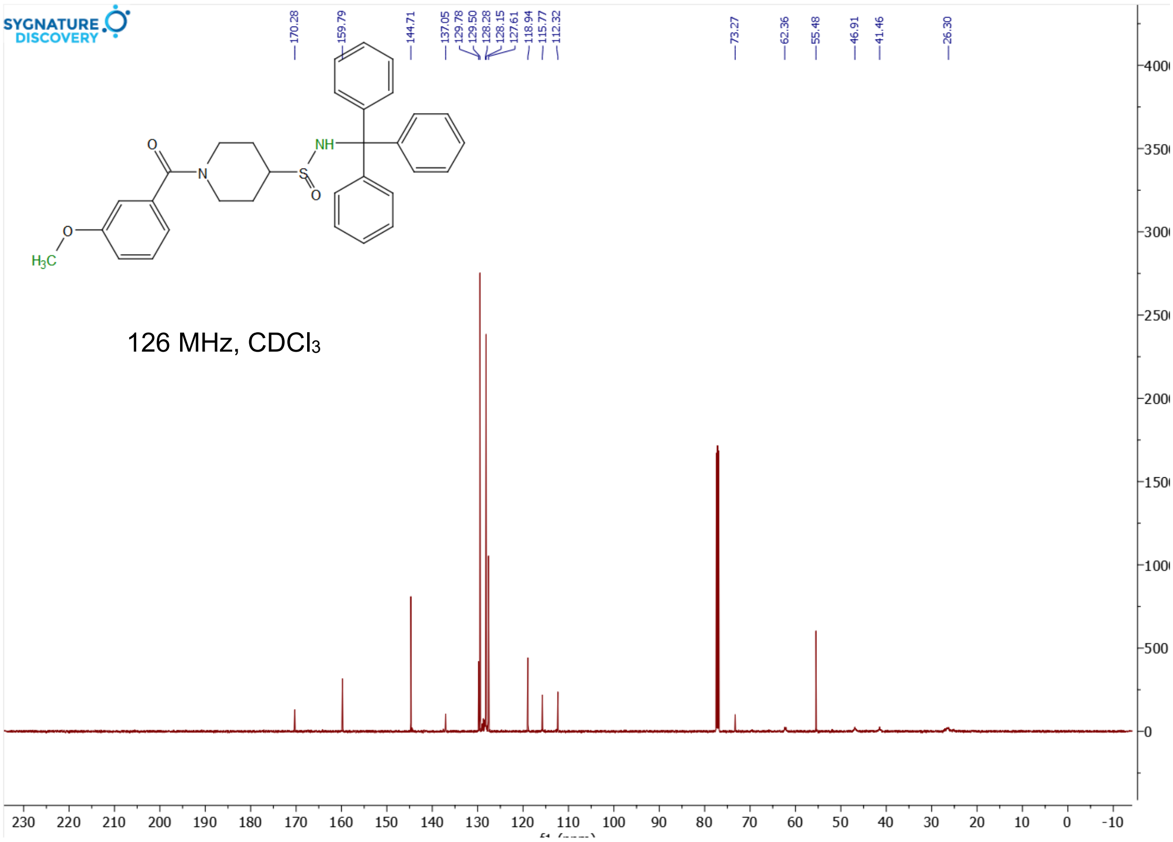

2-Methyl-*N*-tritylpropane-2-sulfinamide 1r

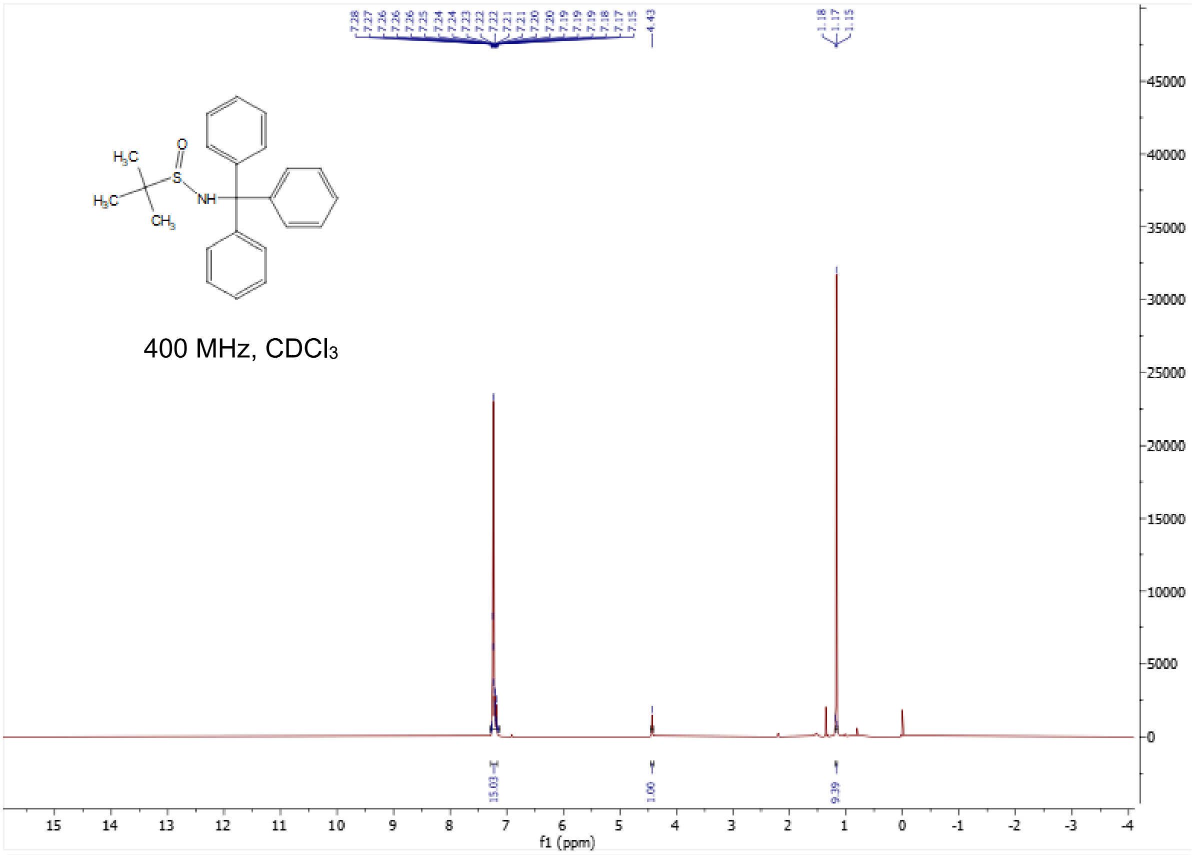

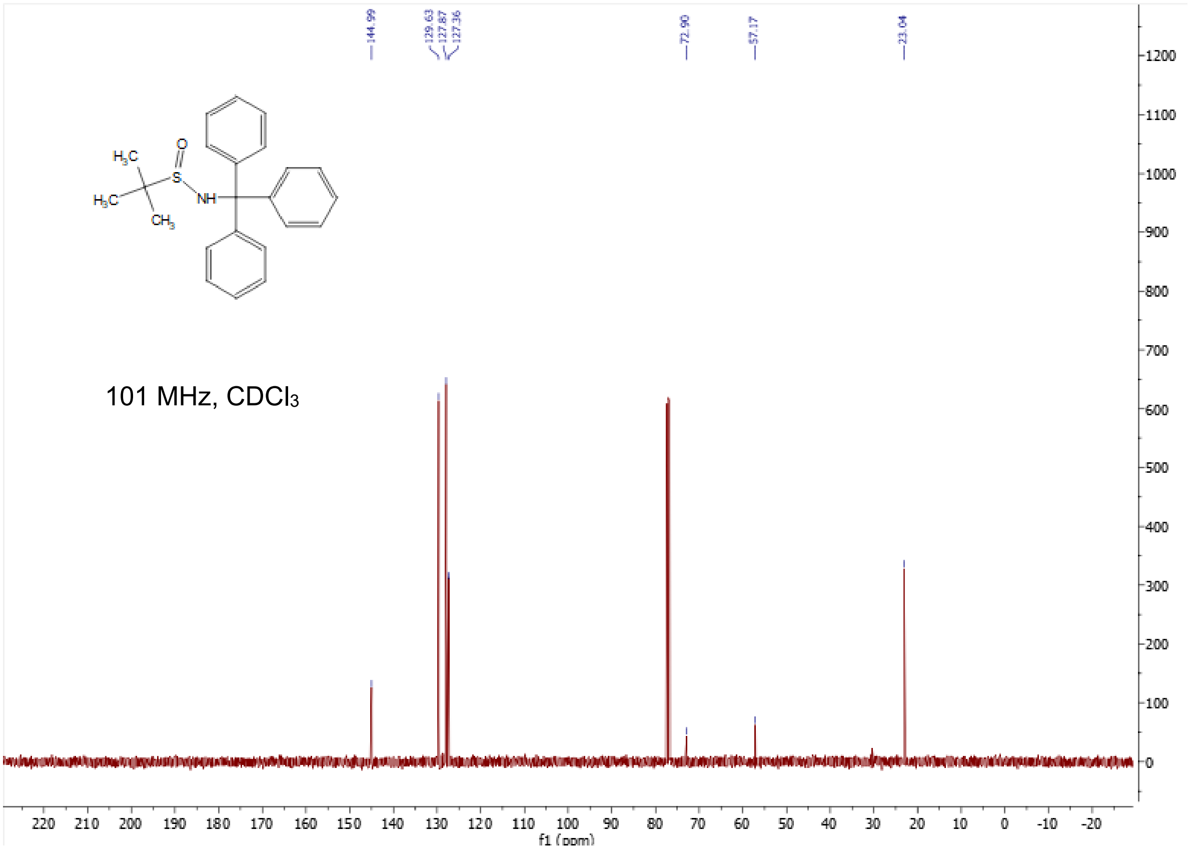

4-fluoro-*N*-(2-((tritylamino)sulfinyl)ethyl)benzamide 1s

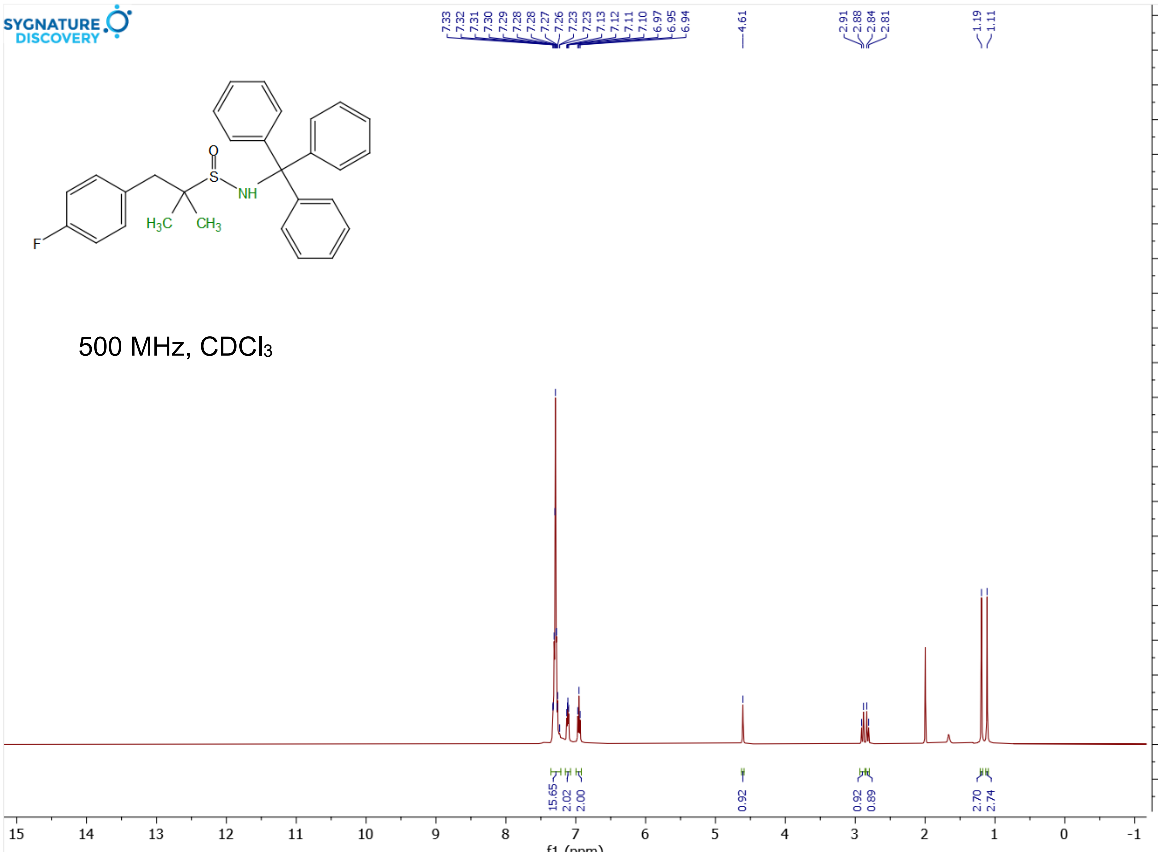

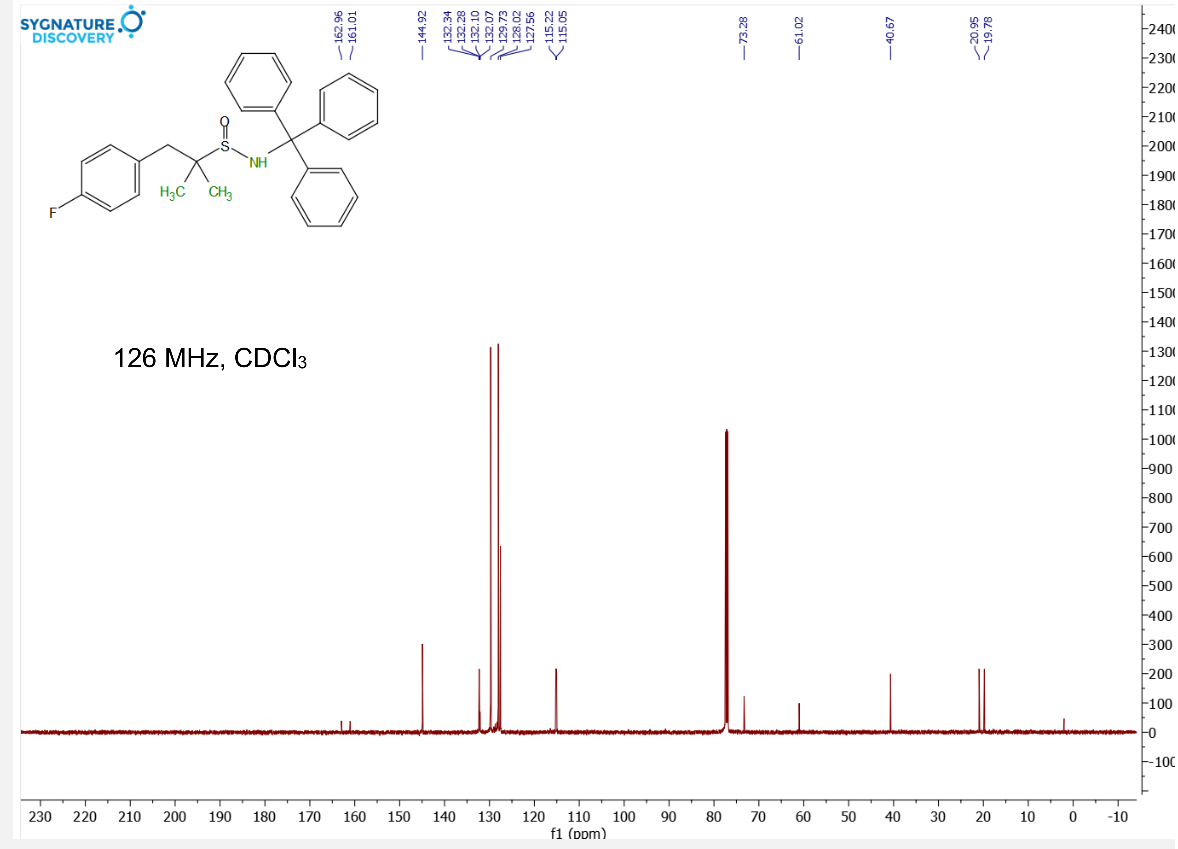

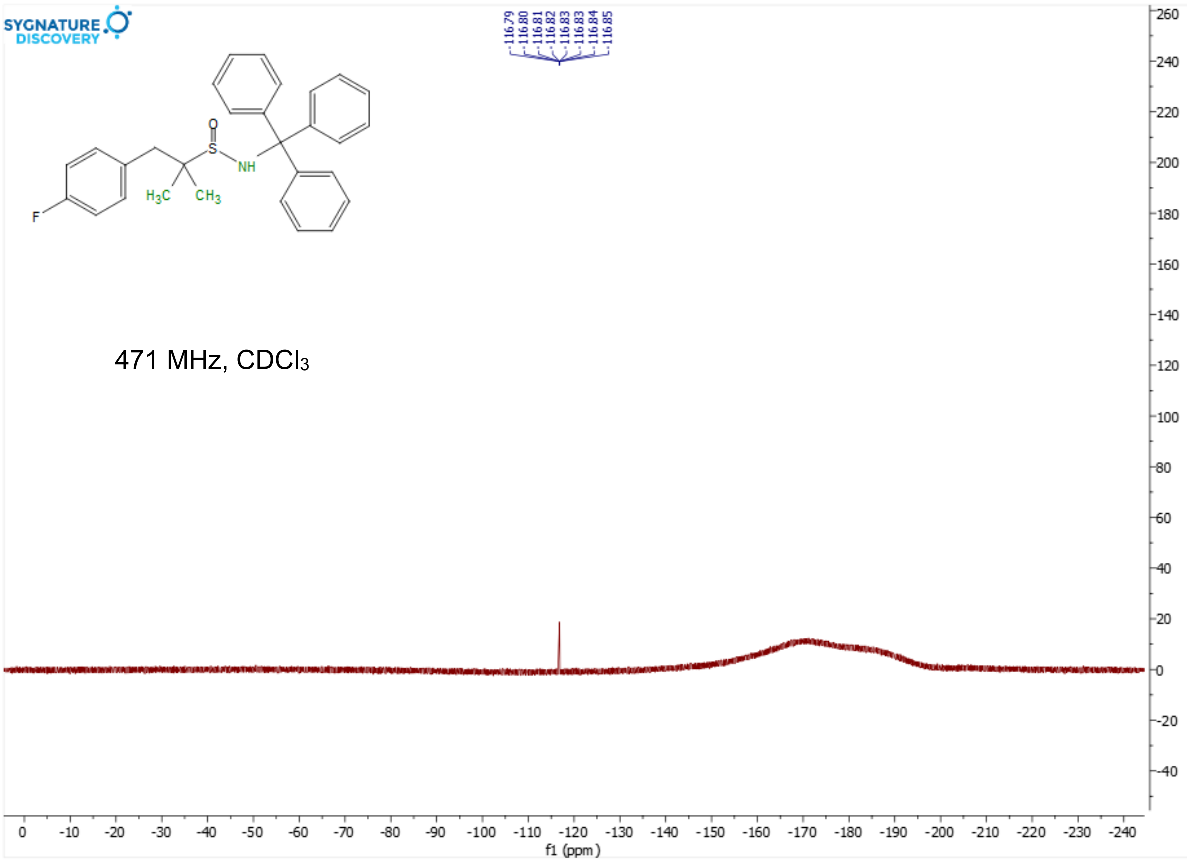
SYGNATURE DISCOVERY
471 MHz, CDCl3
-116.79
-116.80
-116.81
-116.82
-116.83
-116.83
-116.84
-116.85
f1 (ppm)

N-trityladamantane-1-sulfinamide 1t

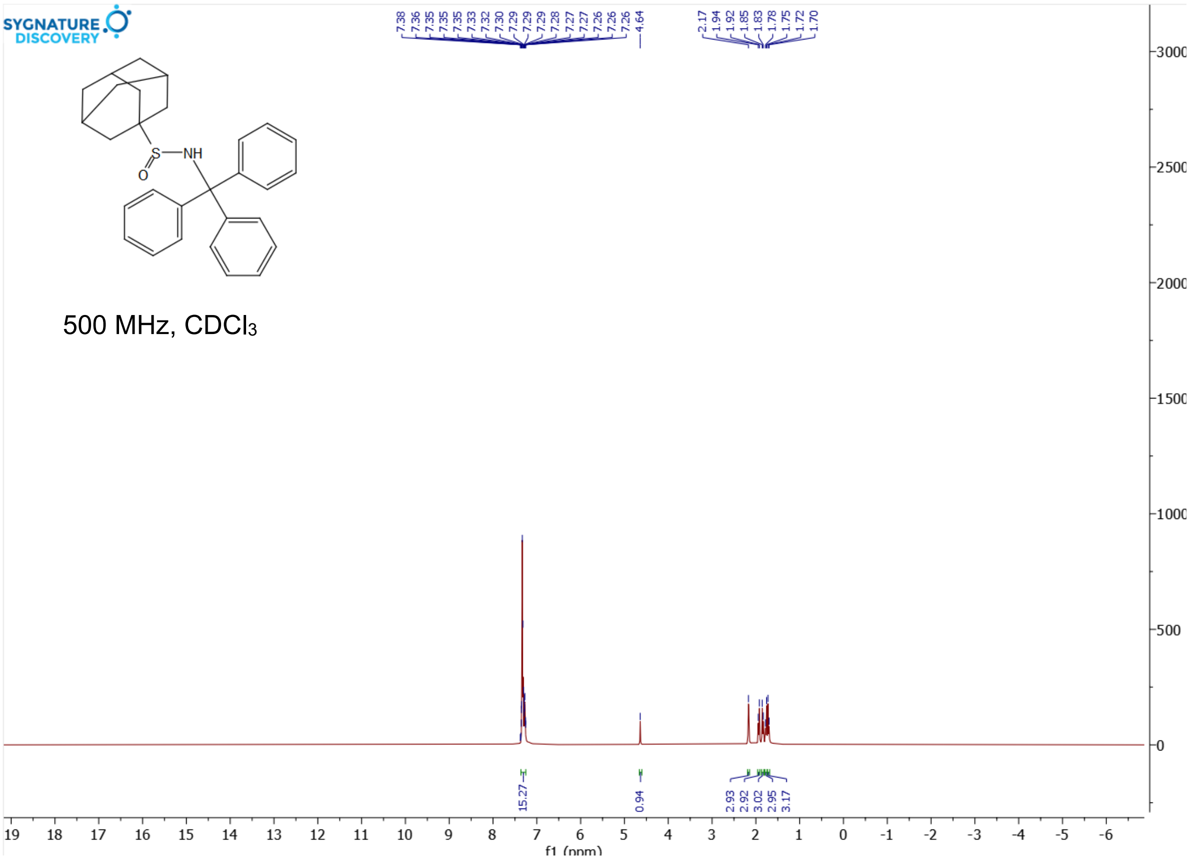

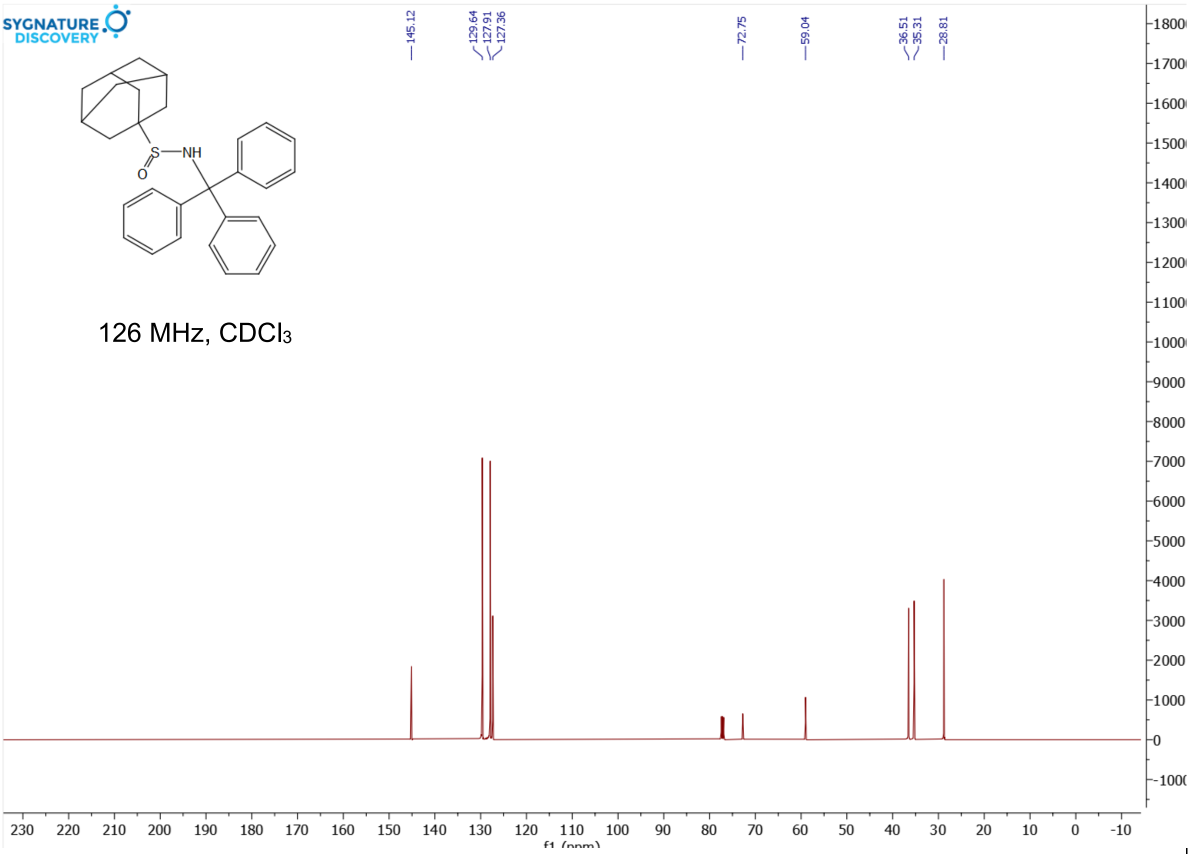

1-(quinolin-6-yl)-*N*-tritylmethanesulfinamide 1u

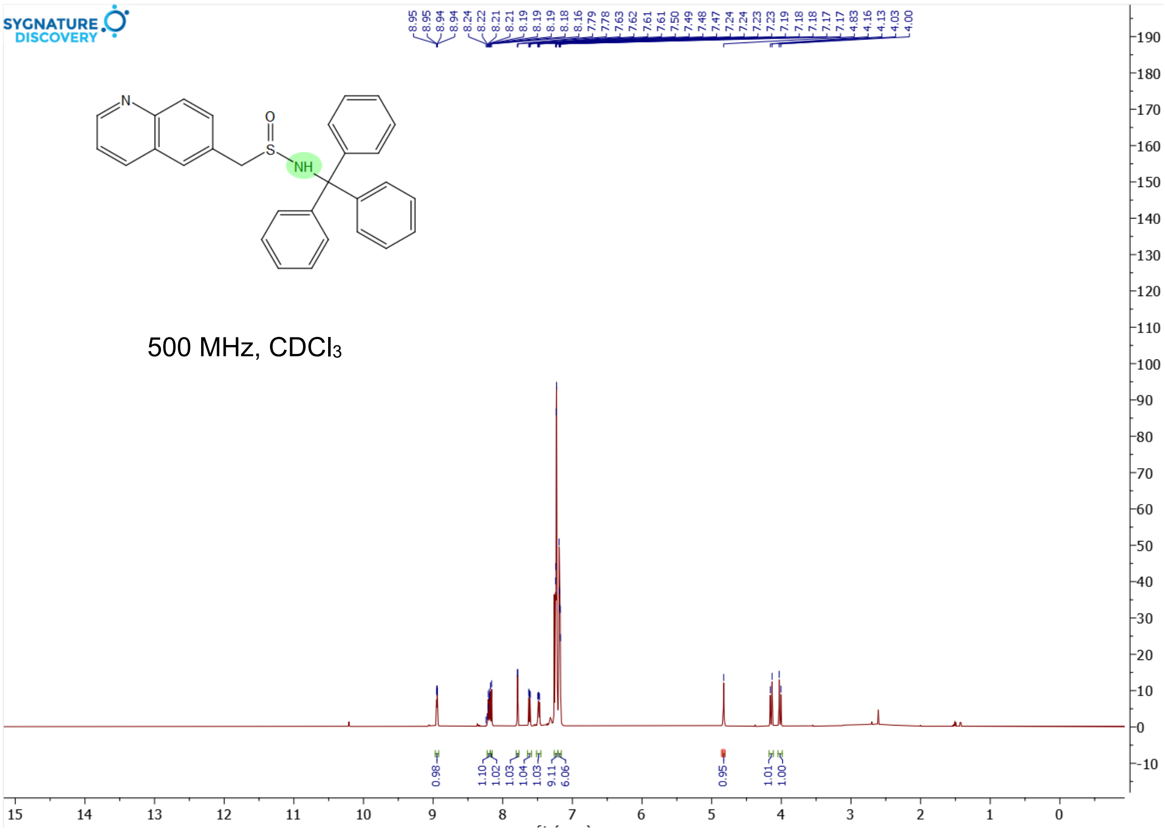

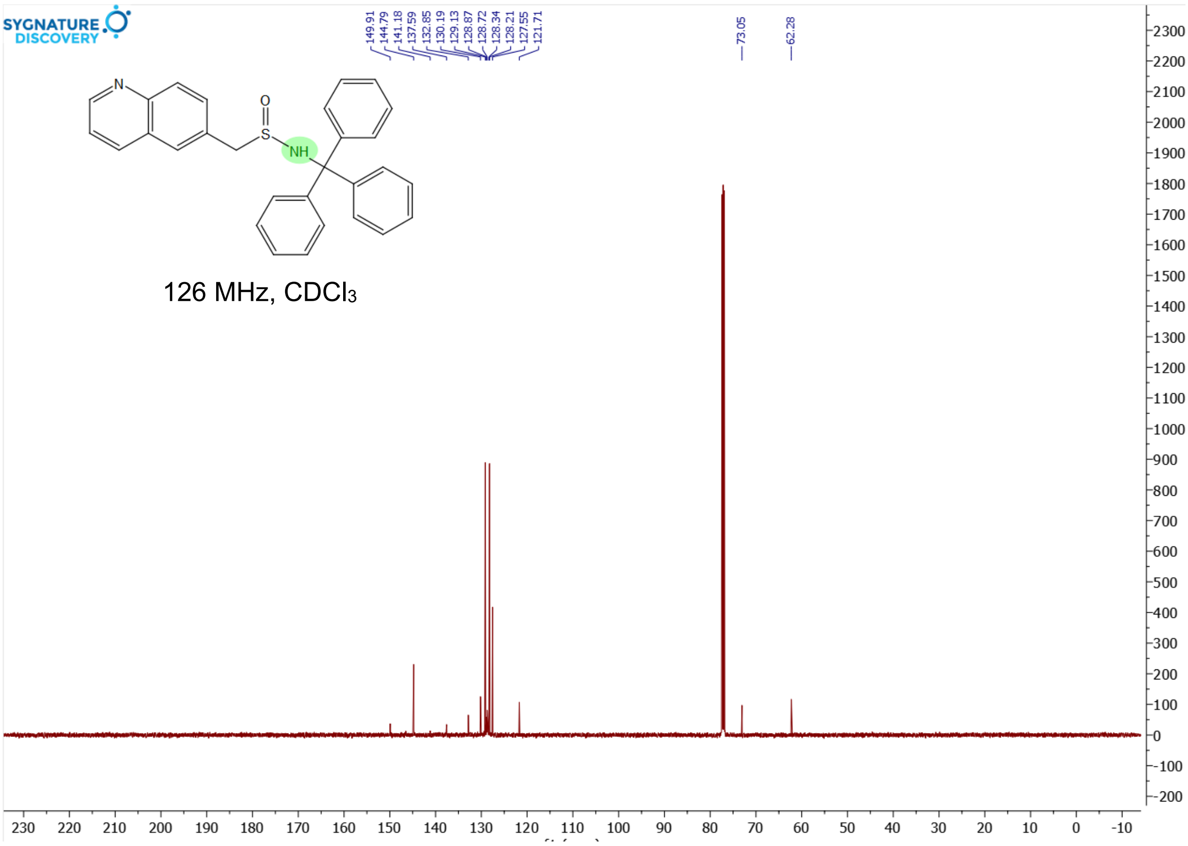

tert-Butyl 2-(((tritylamino)sulfinyl)methyl)-7-azaspiro[3.5]nonane-7-carboxylate 1v

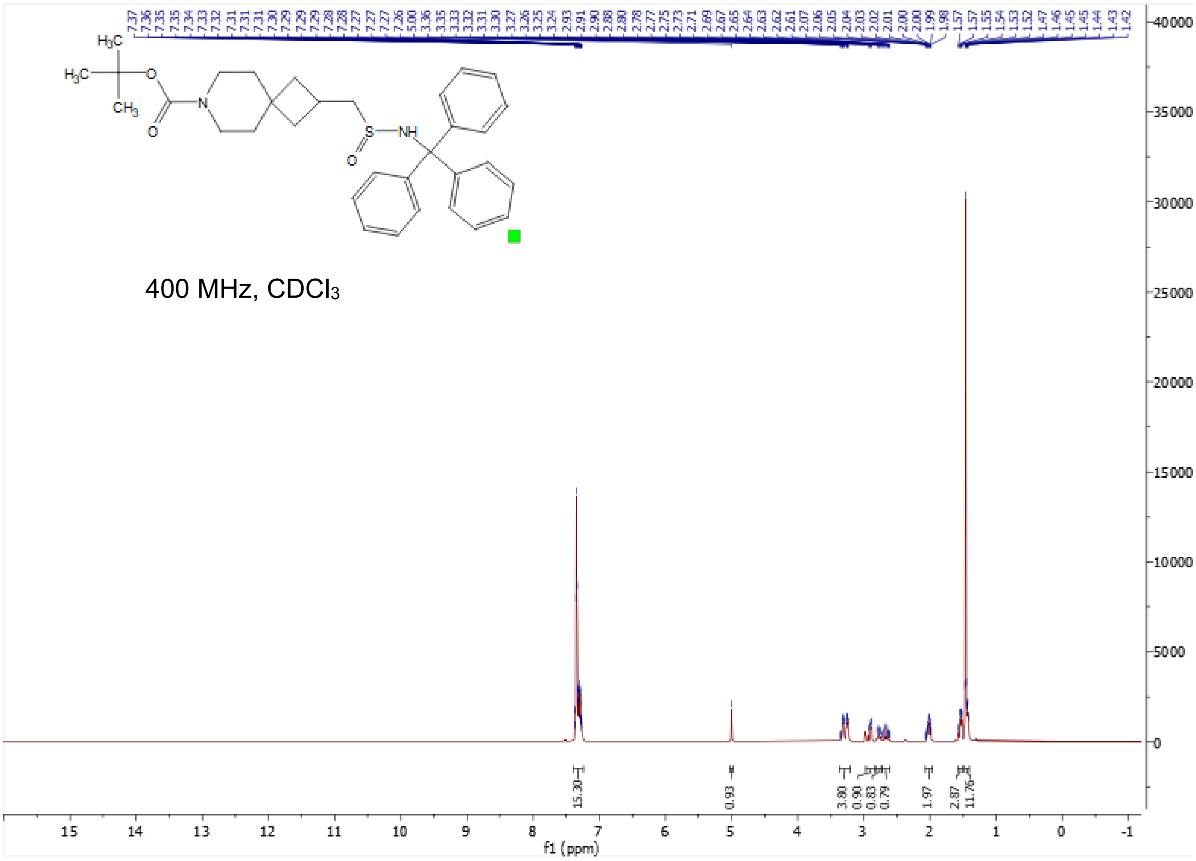

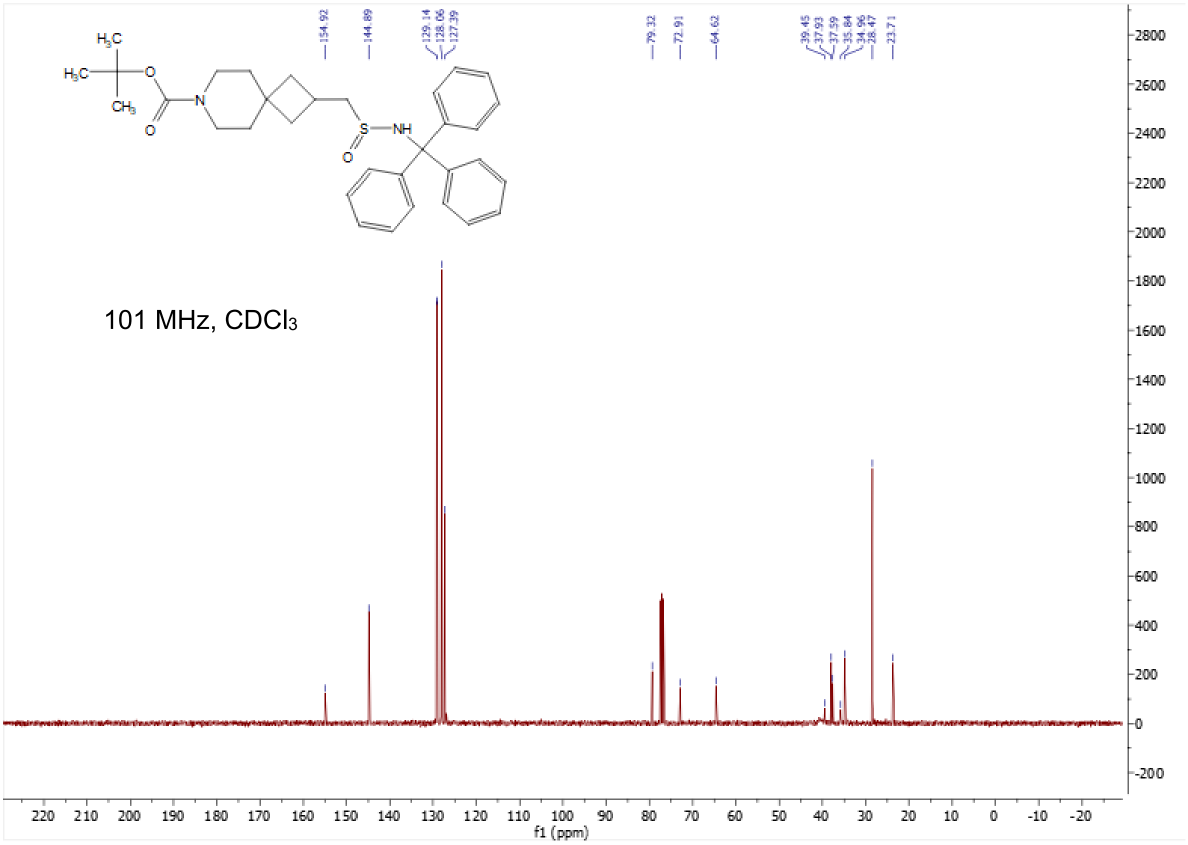

Tert-butyl 4-(5-(3-((tritylamino)sulfinyl)bicyclo[1.1.1]pentan-1-yl)-1,2,4-oxadiazol-3-yl)piperidine-1-carboxylate 1w

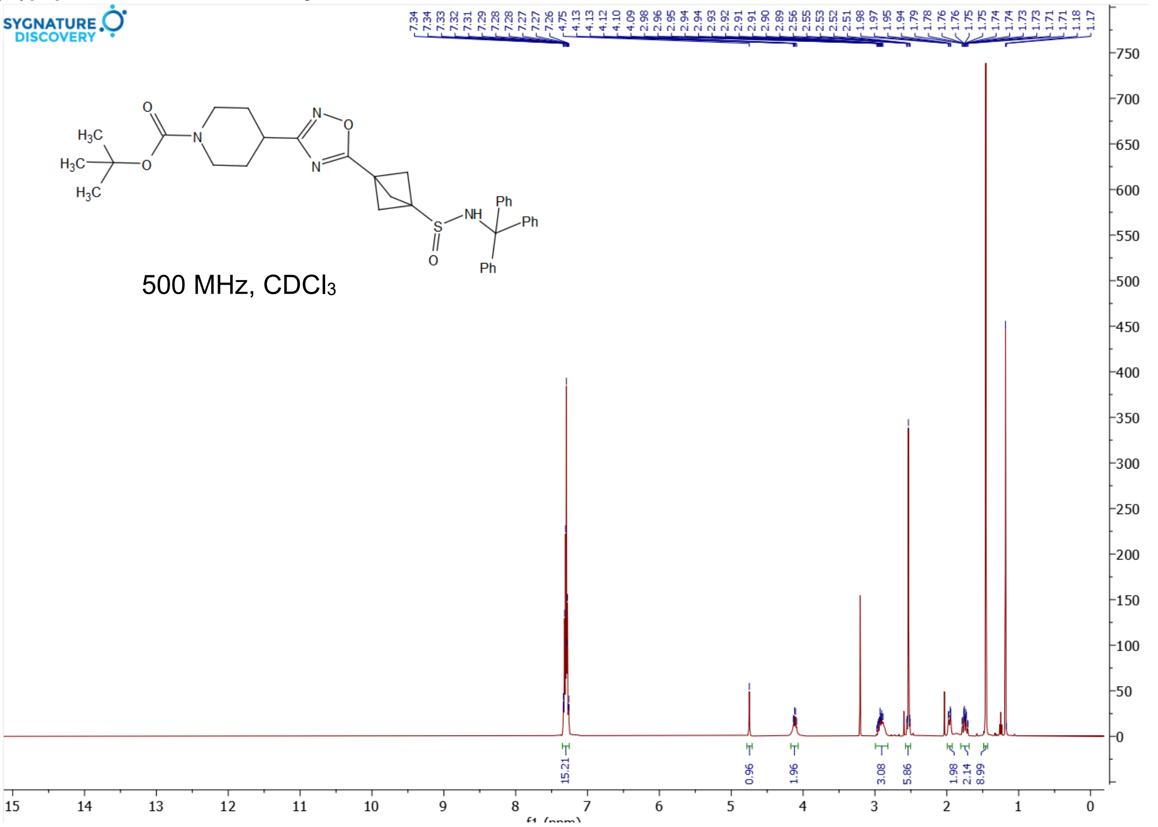

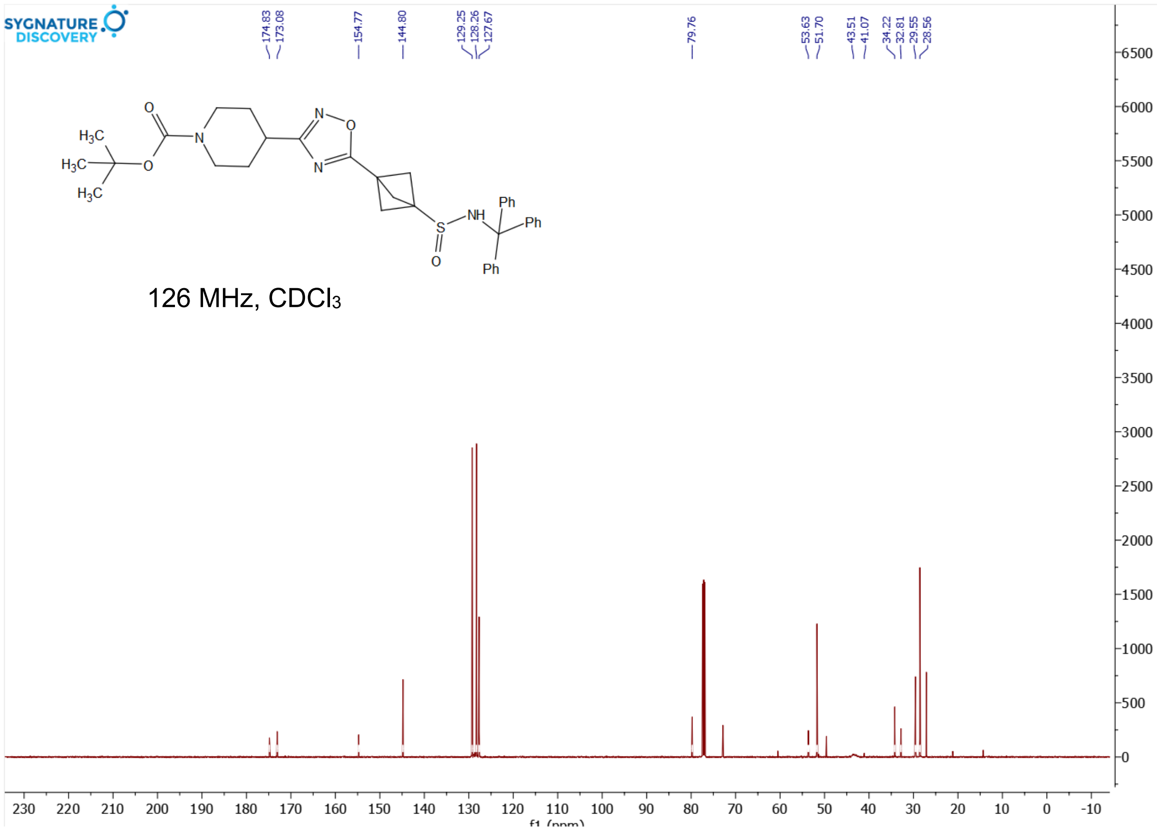

(3R)-3-((3R,5S,7R,8R,10S,13R,14S,17R)-3,7-dihydroxy-10,13-dimethylhexadecahydro-1H-cyclopenta[a]phenanthren-17-yl)-*N*-tritylbutane-1-sulfinamide 1x

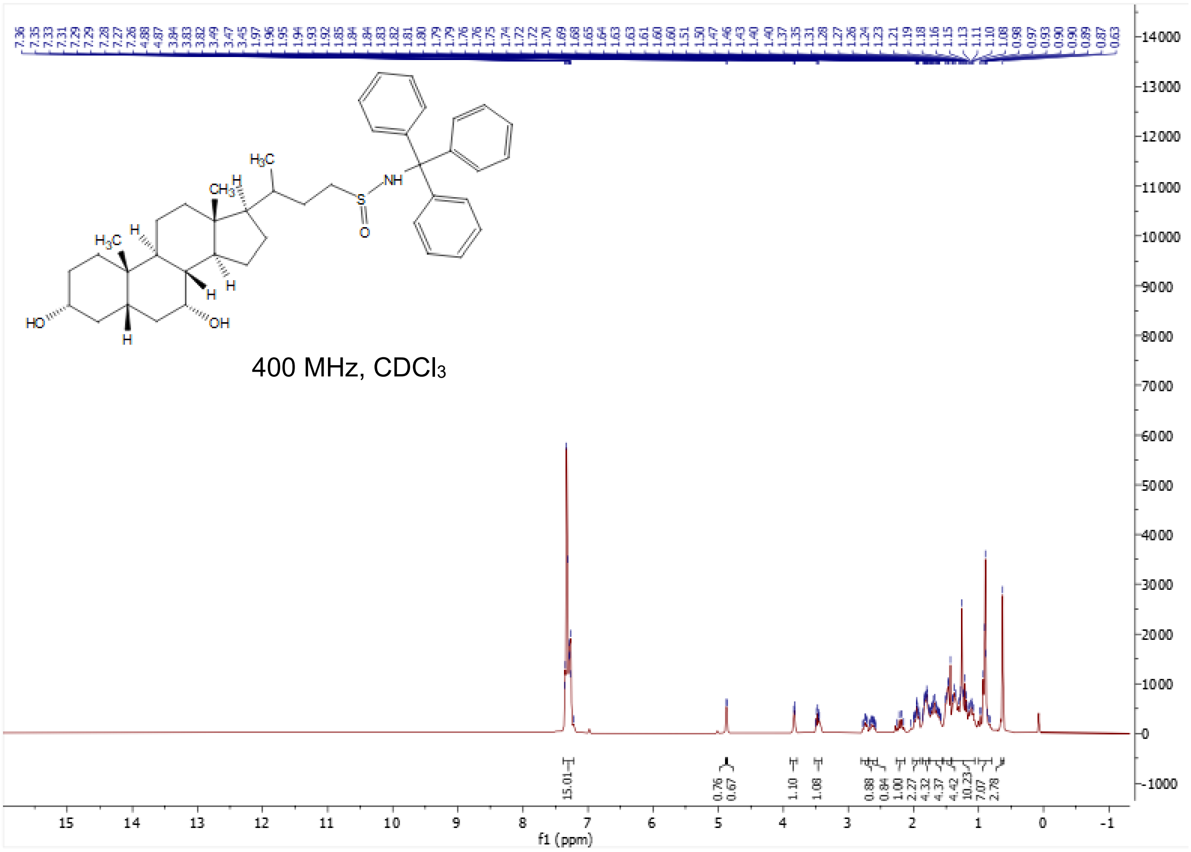

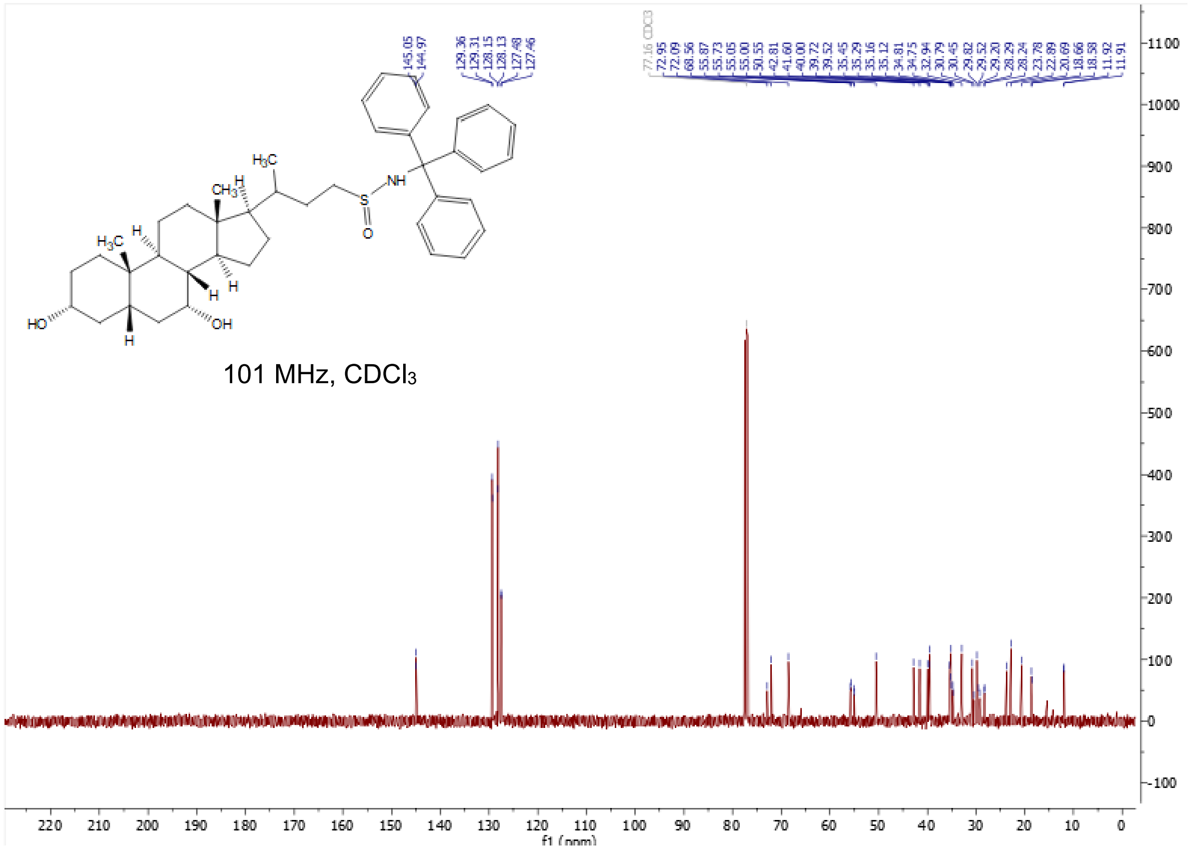

2-phenyl-*N*-(2,4,4-trimethylpentan-2-yl)ethane-1-sulfinamide 2a

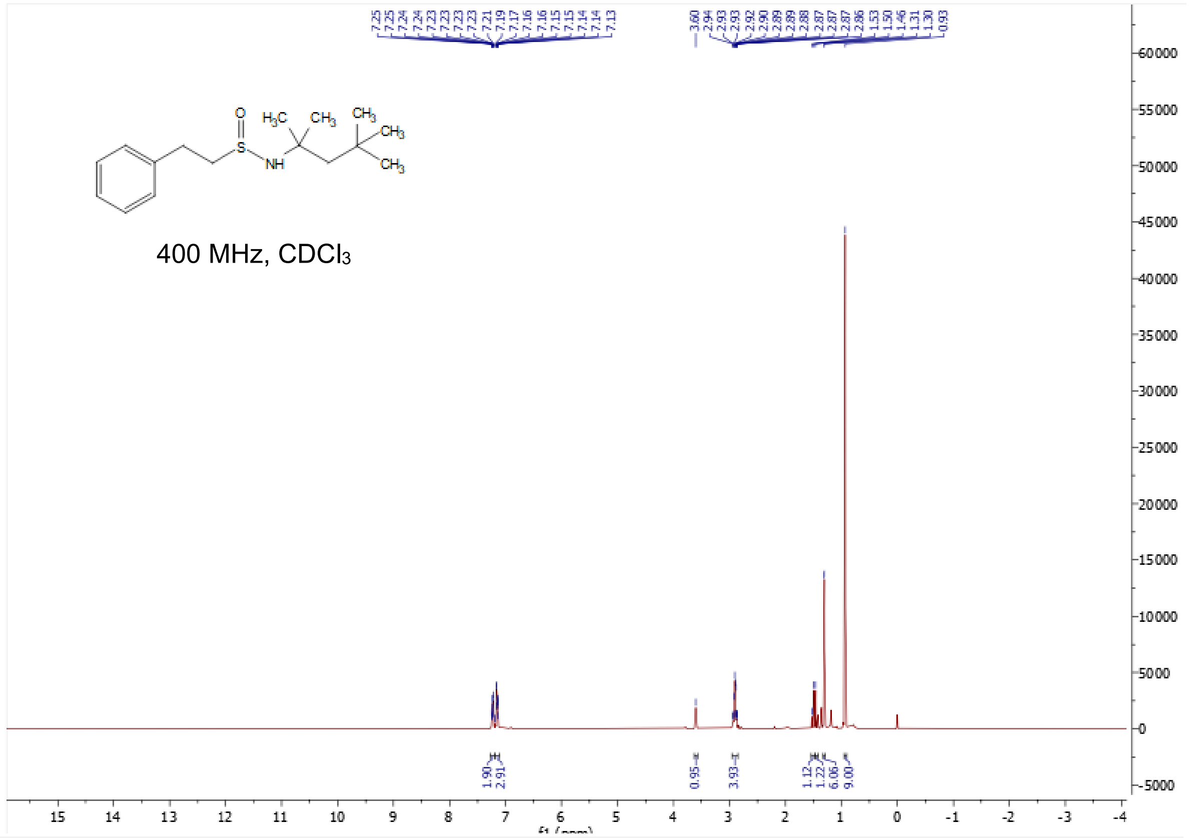

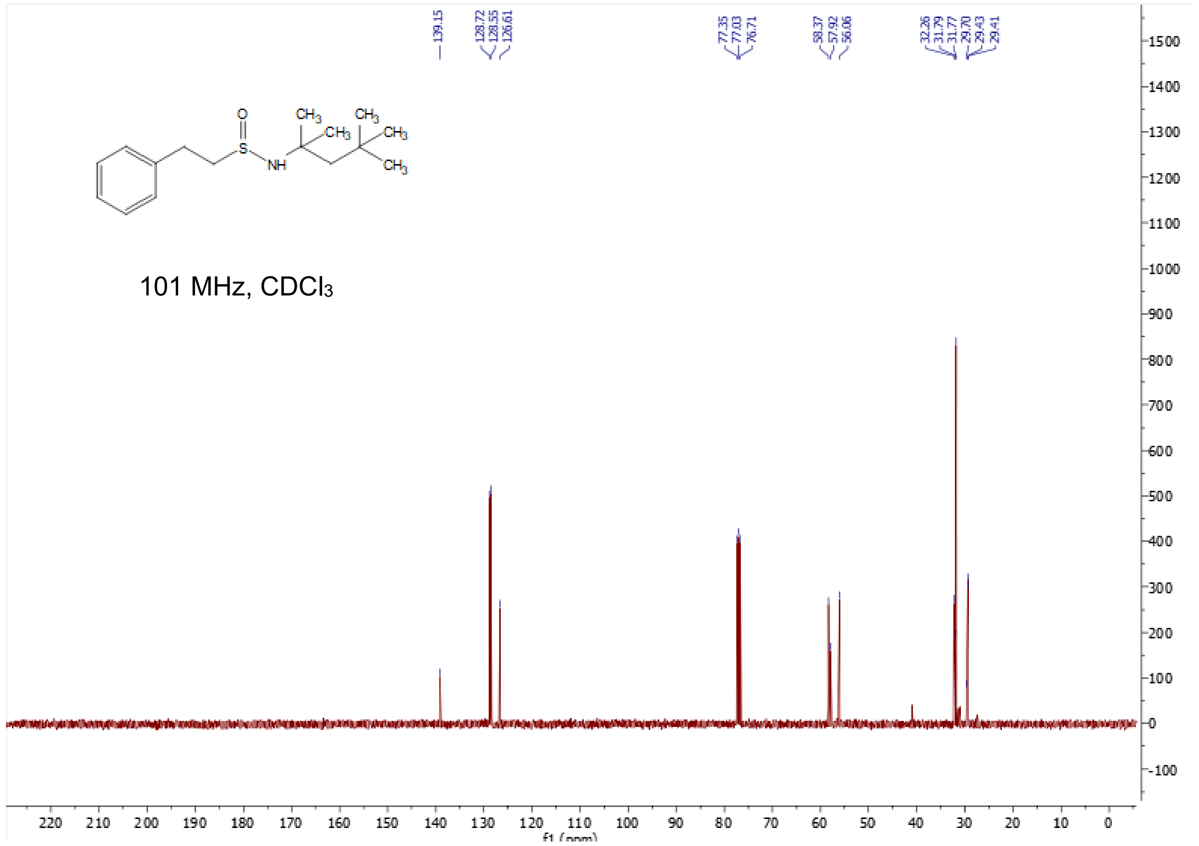

N-(tert-butoxy)-2-phenylethane-1-sulfinamide 2b

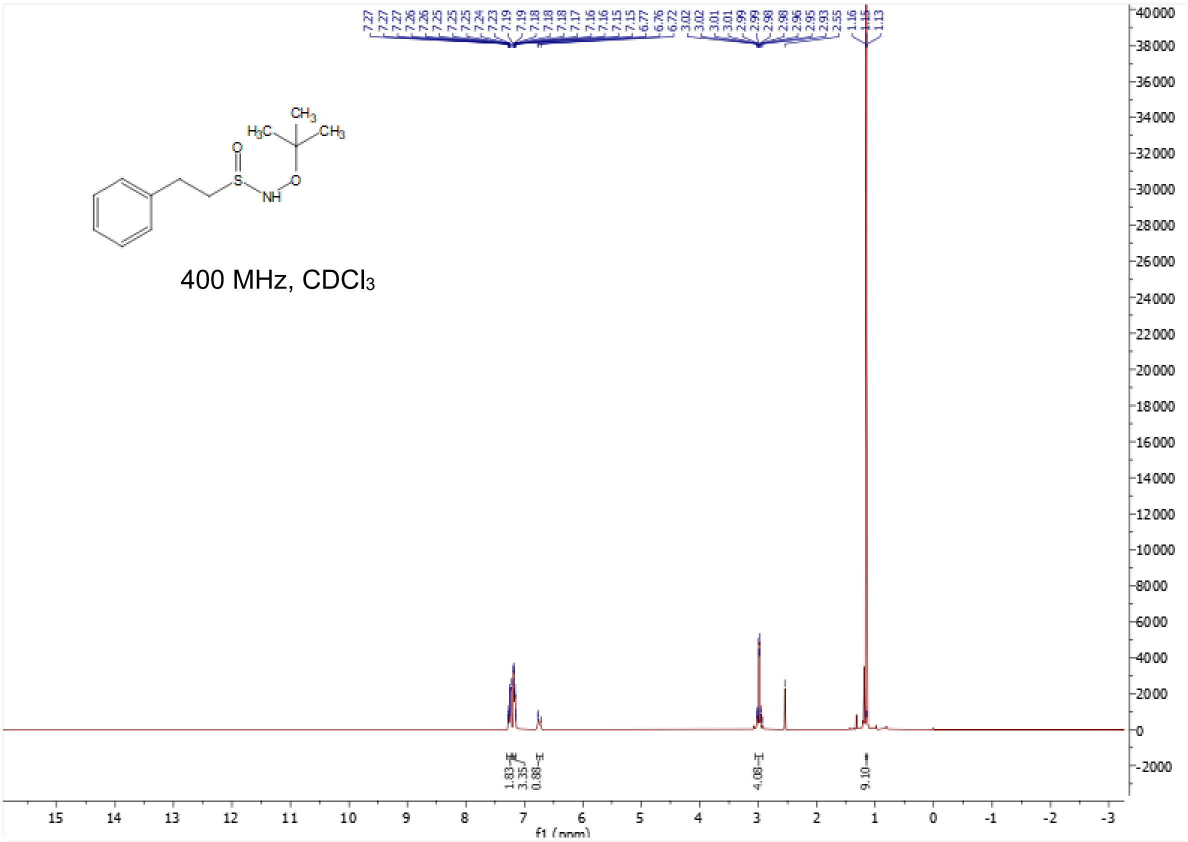

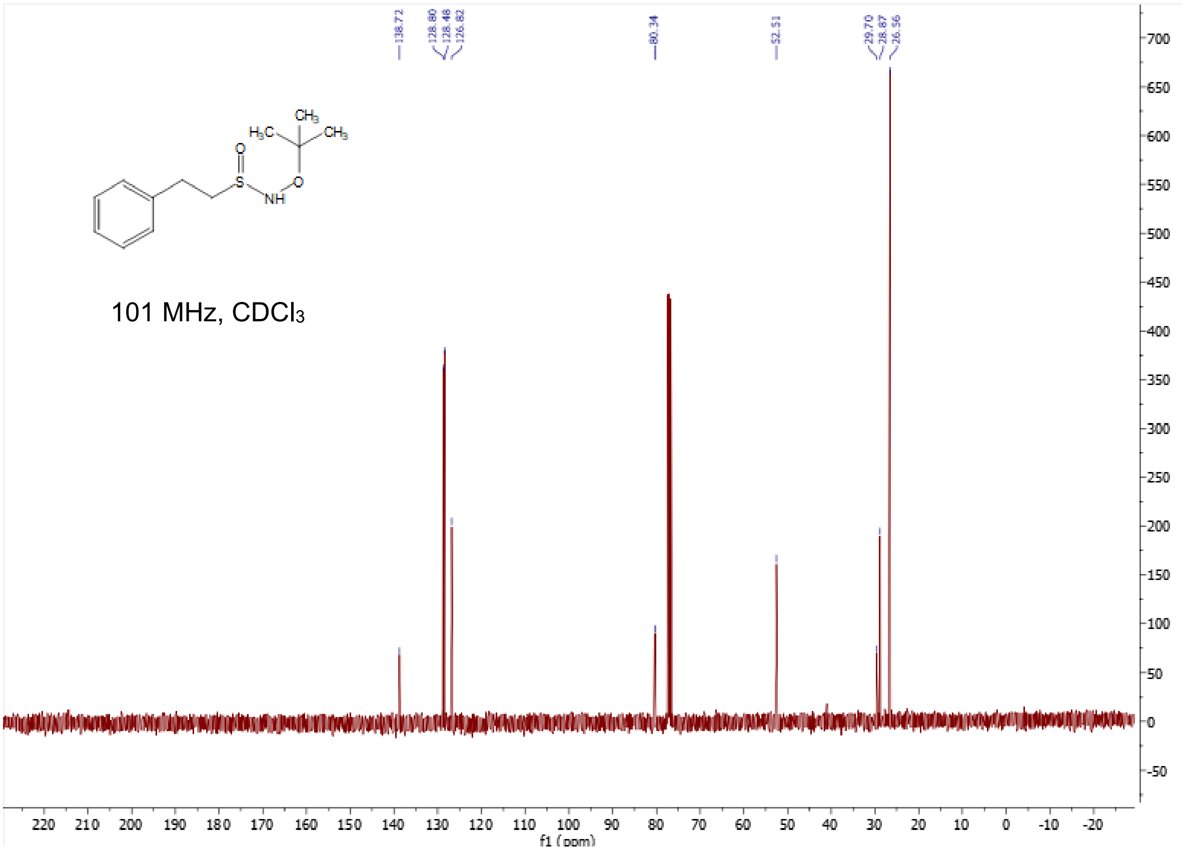

2-phenyl-*N*-(triisopropylsilyl)ethane-1-sulfinamide 2c

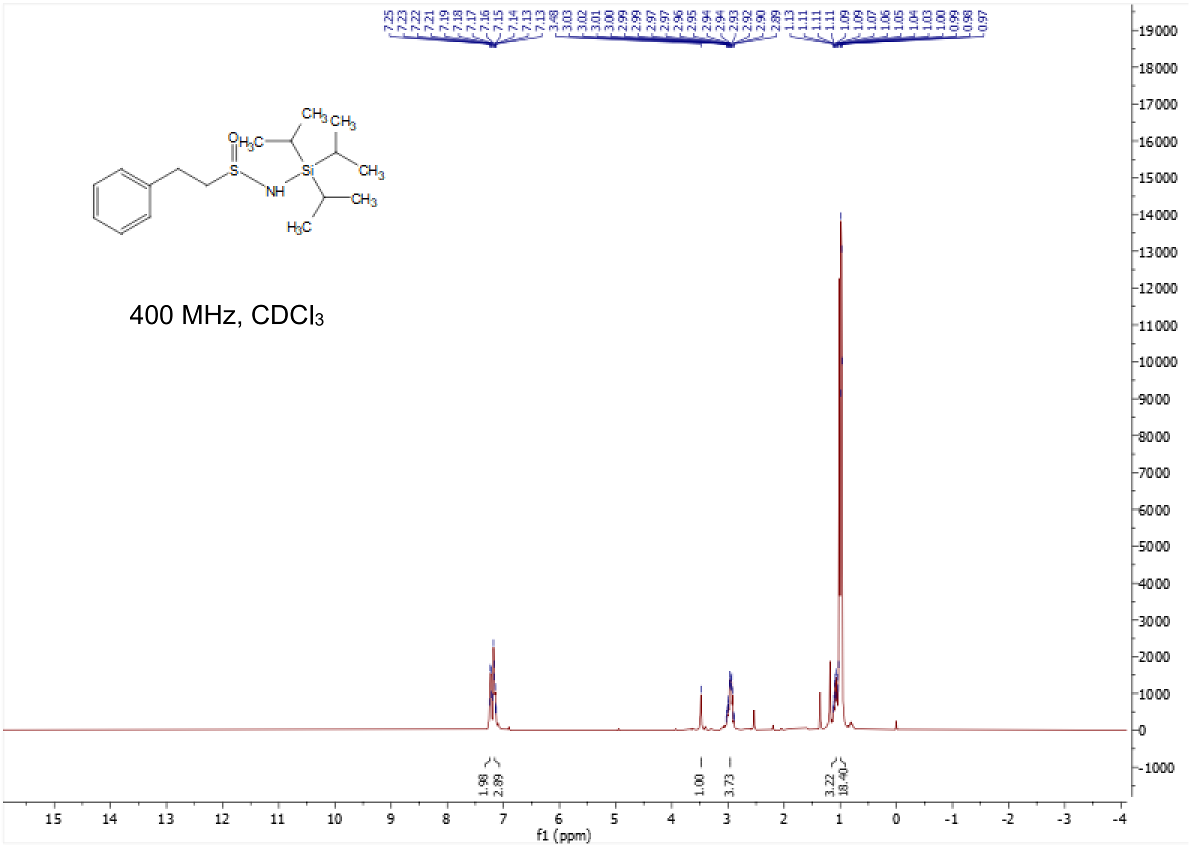

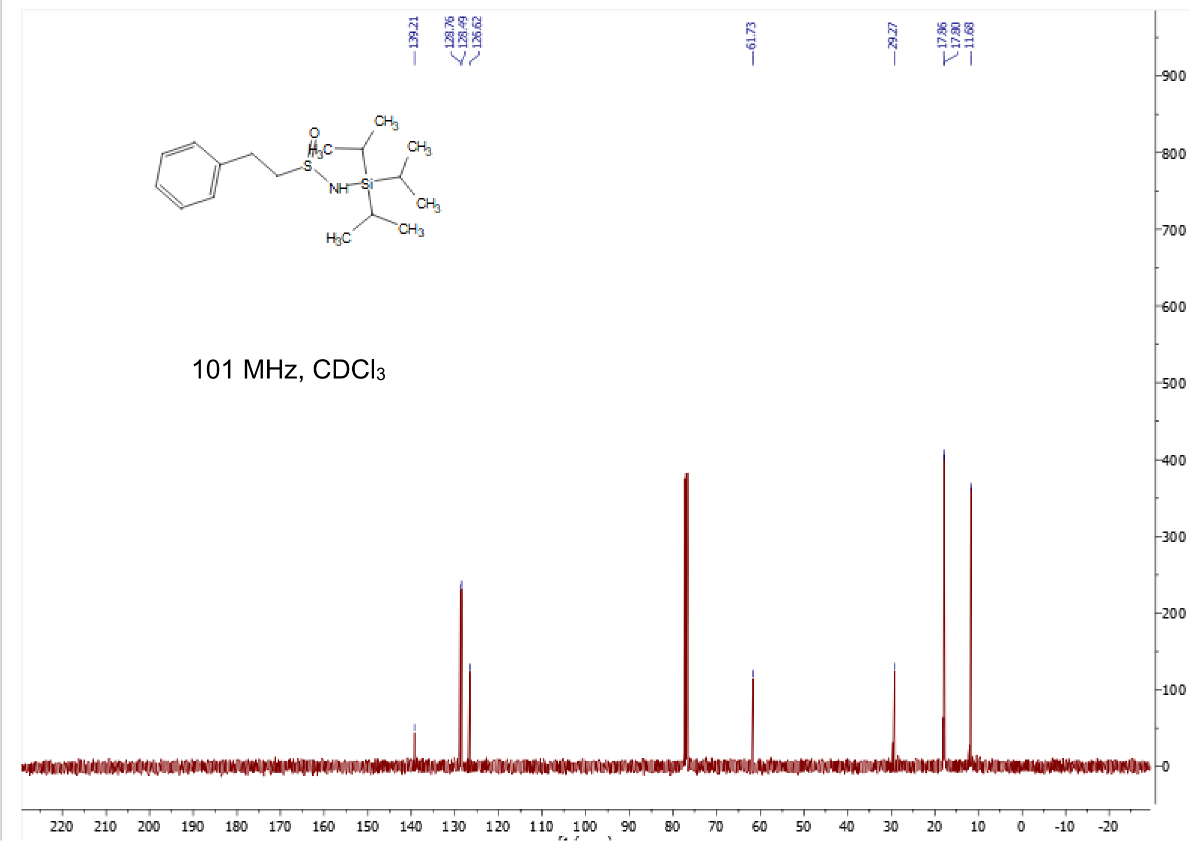

Methyl 3-(((triisopropylsilyl)amino)sulfinyl)propanoate 3a

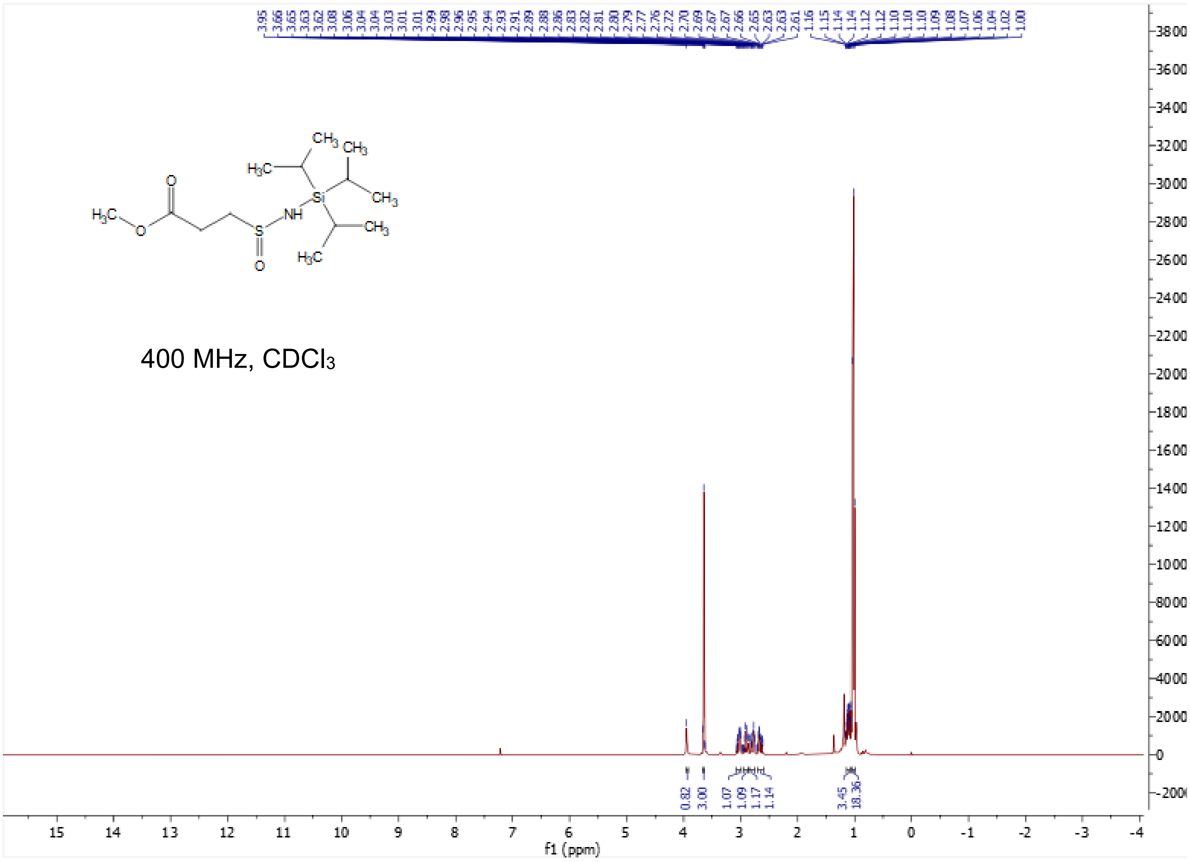

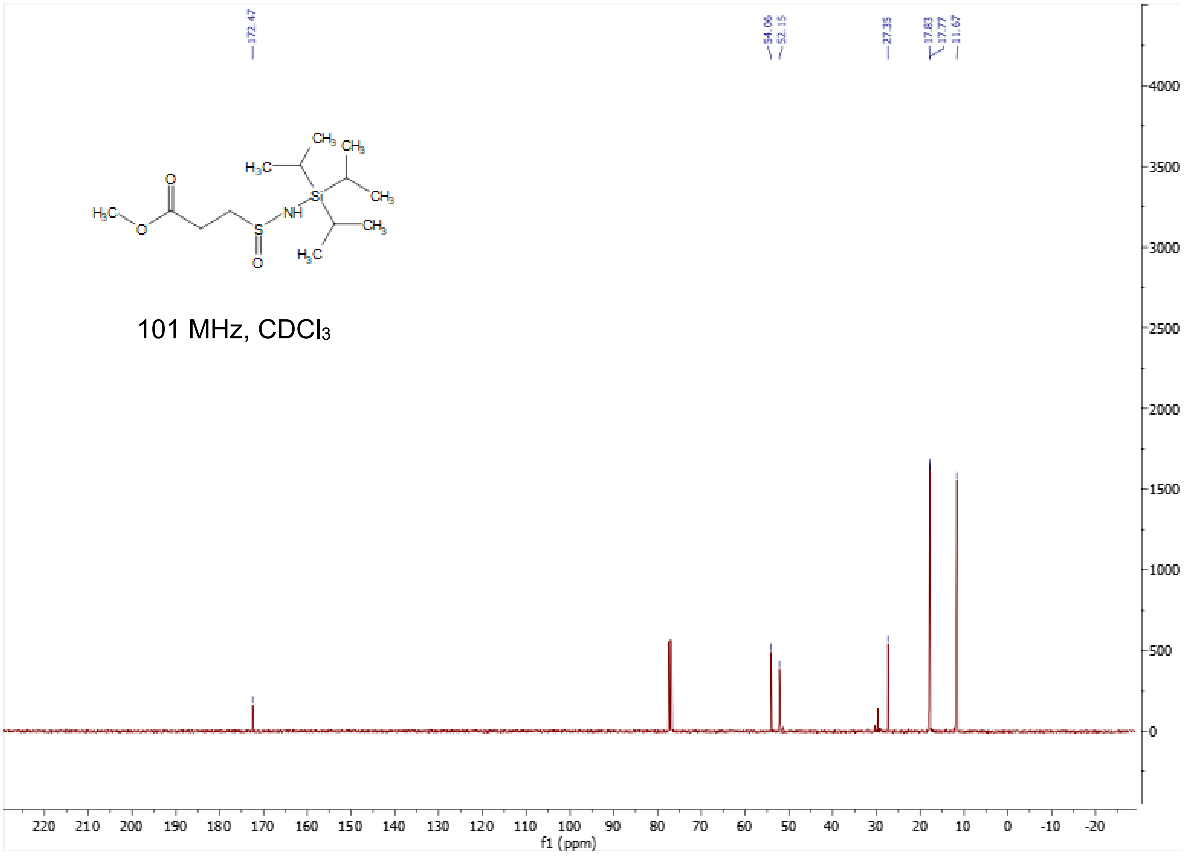

tert-butyl (2-(((triisopropylsilyl)amino)sulfinyl)ethyl)carbamate 3b

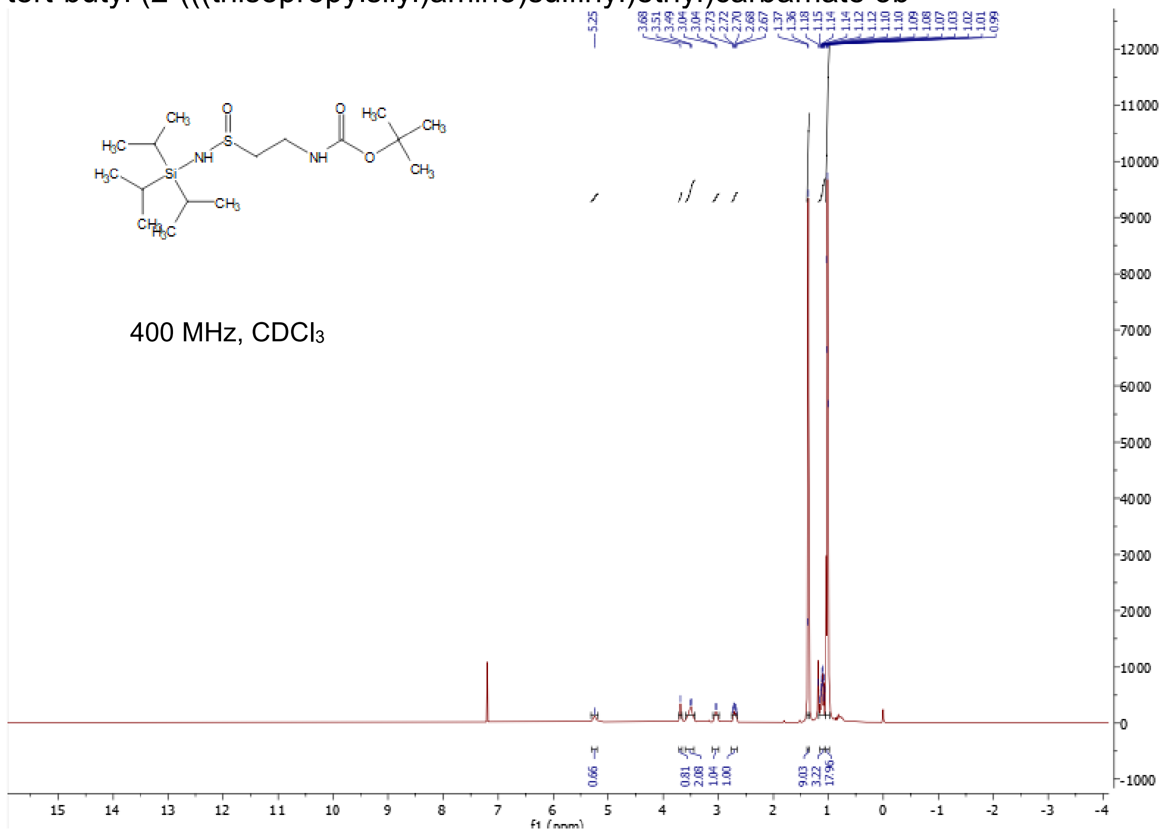

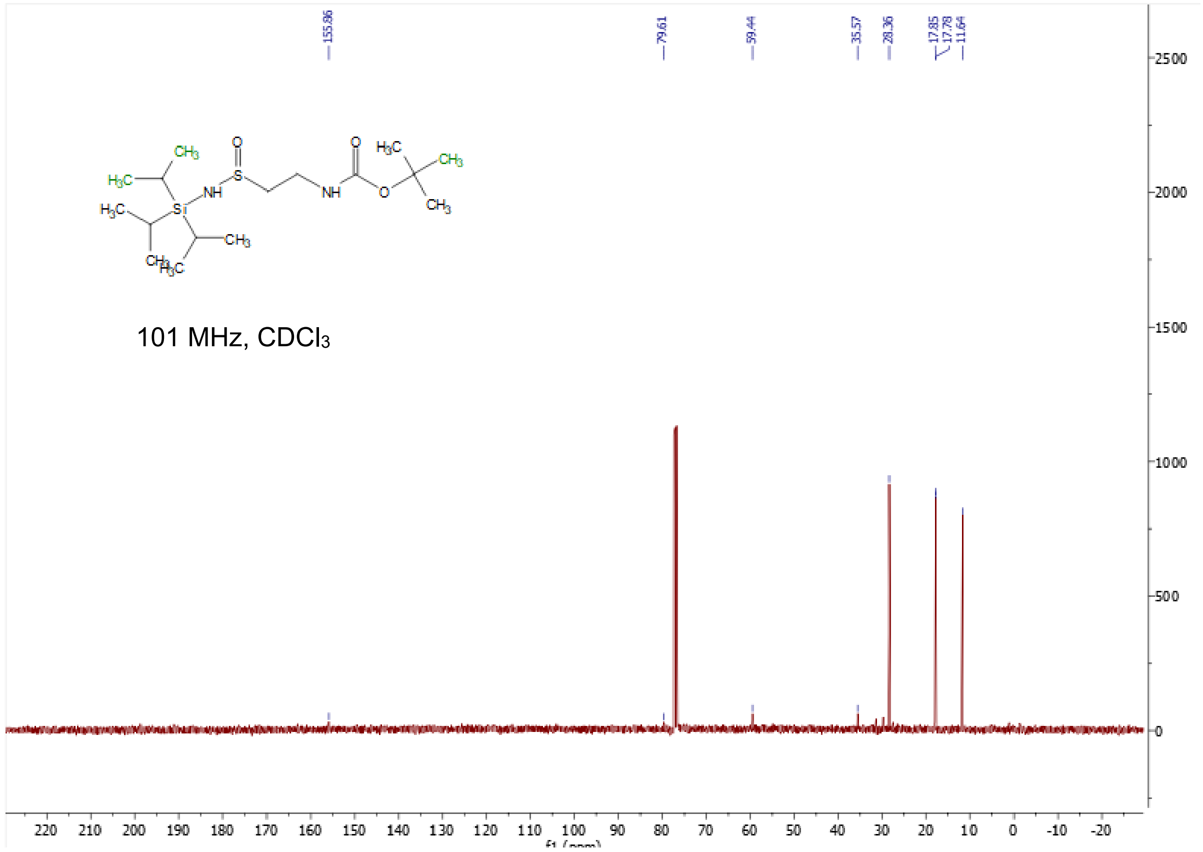

N-(Triisopropylsilyl)hexane-1-sulfinamide 3c

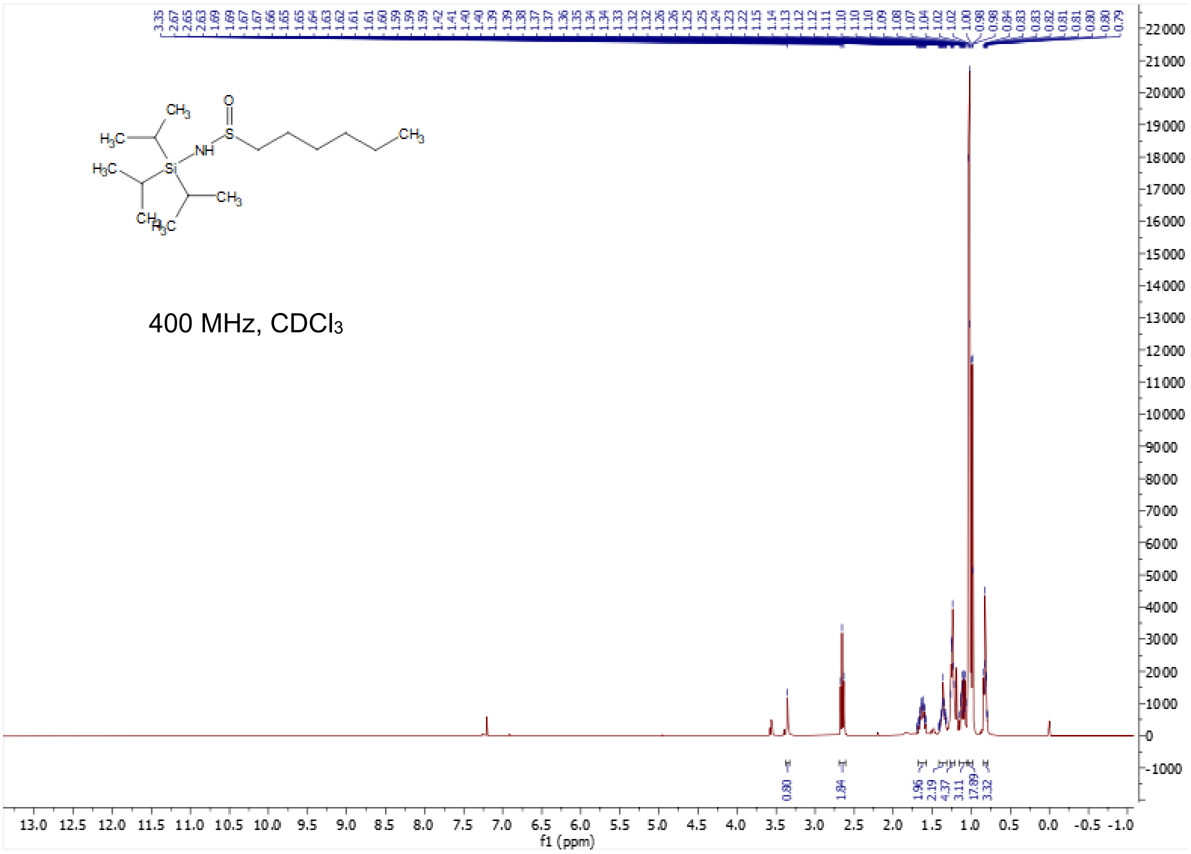

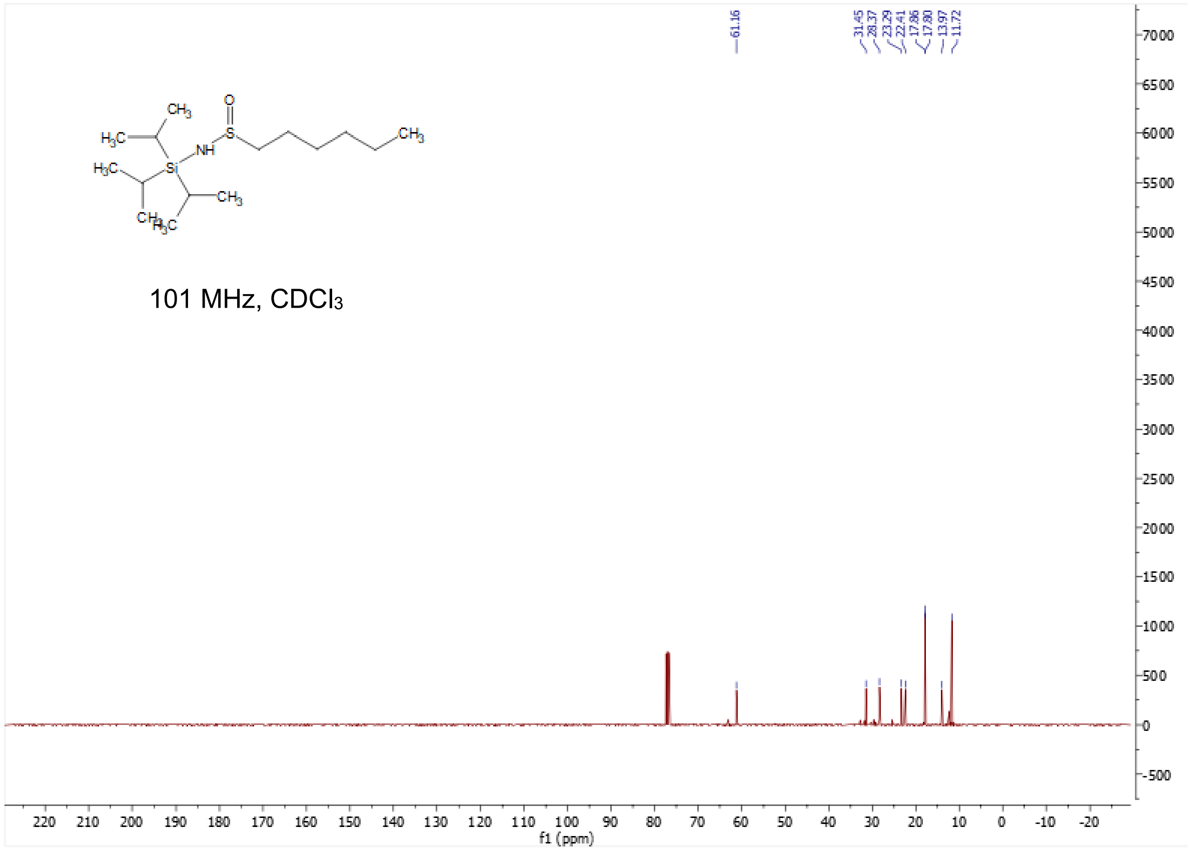

3-Methyl-*N*-(triisopropylsilyl)butane-1-sulfinamide 3d

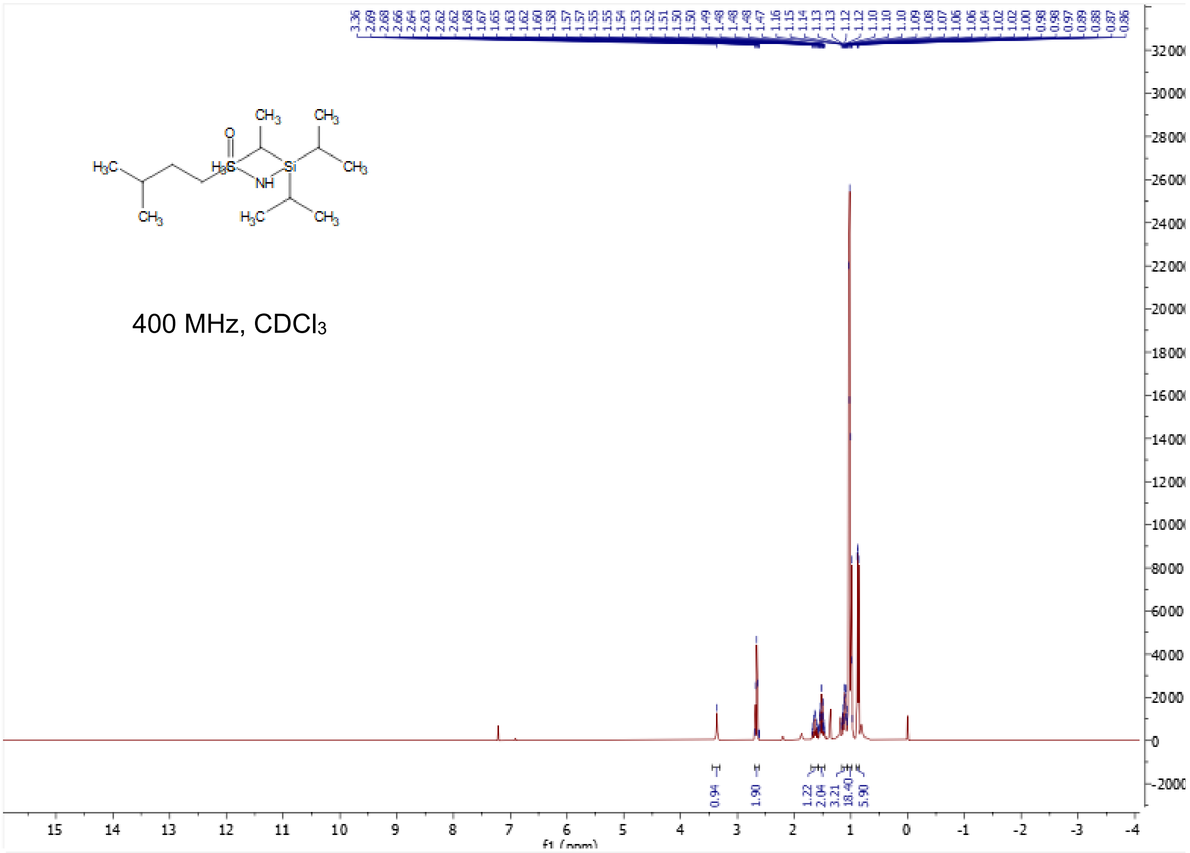

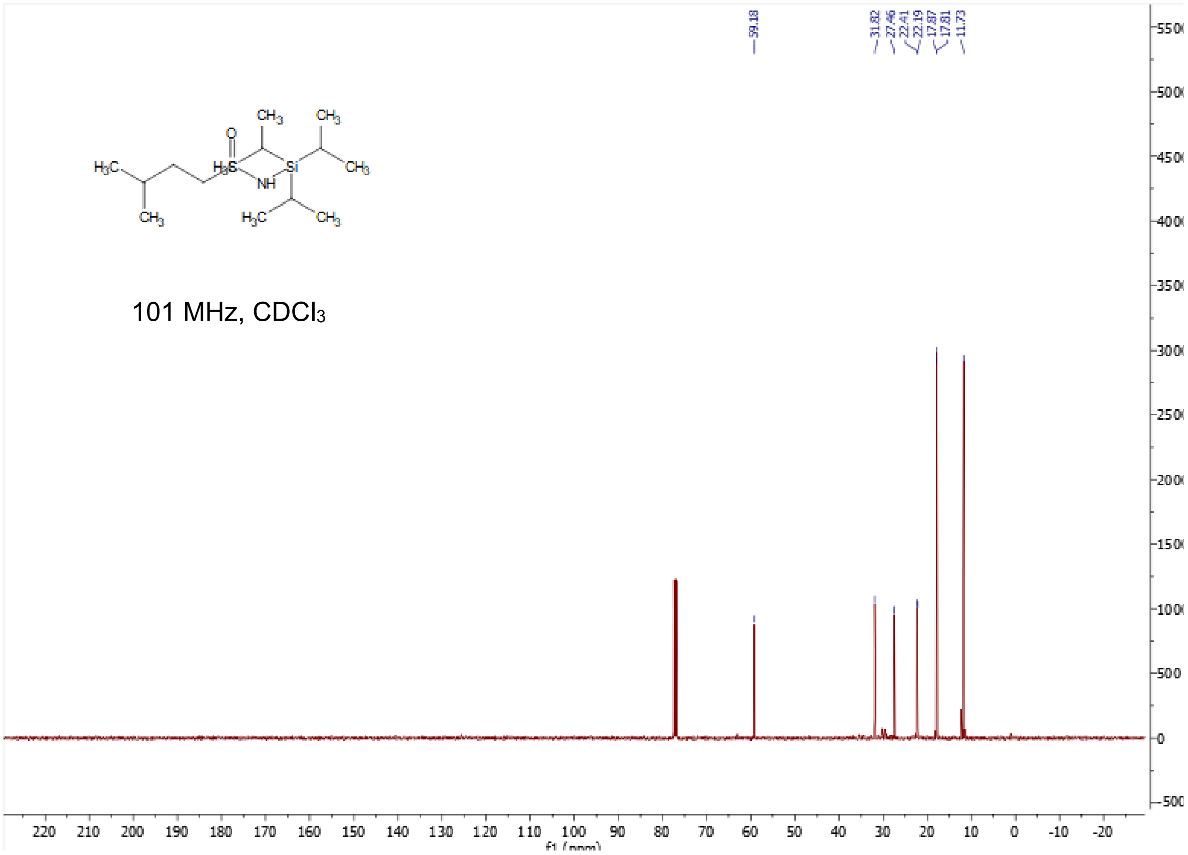

N-(Triisopropylsilyl)propane-2-sulfinamide 3e

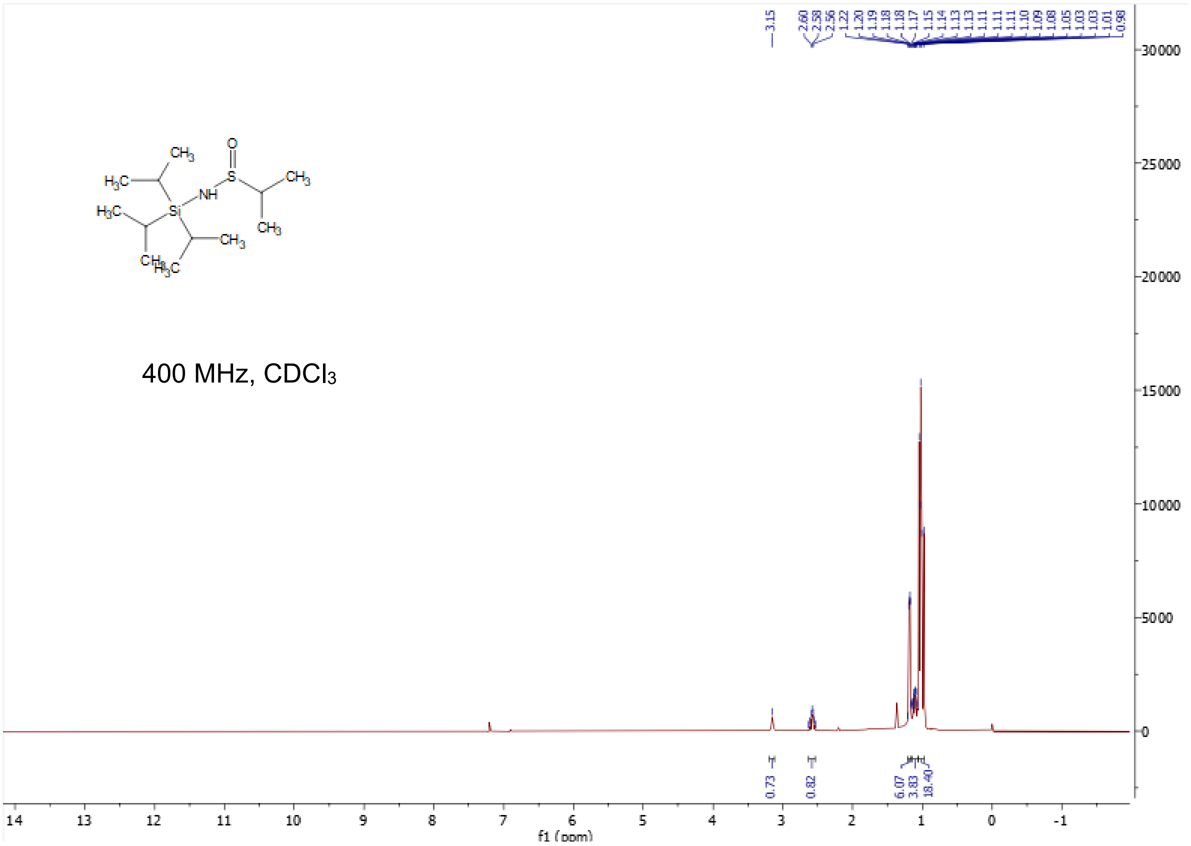

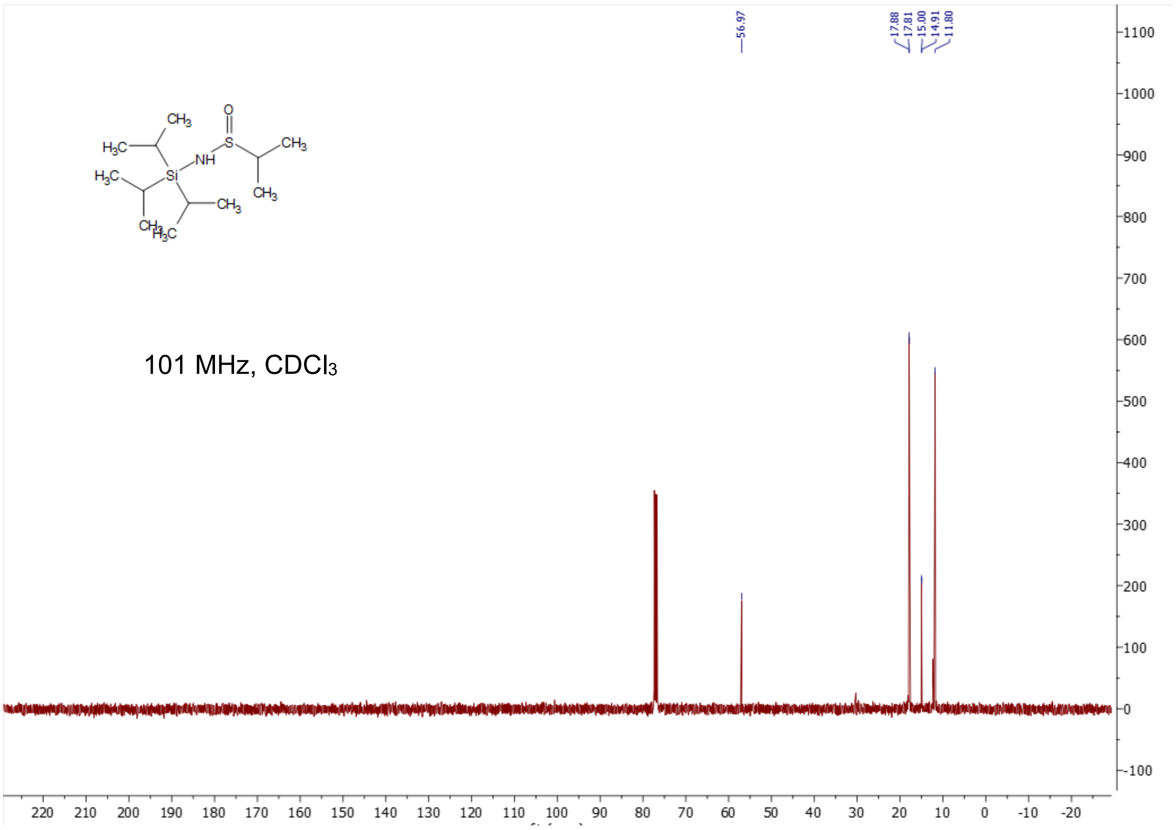

N-(Triisopropylsilyl)cyclopentanesulfinamide 3f

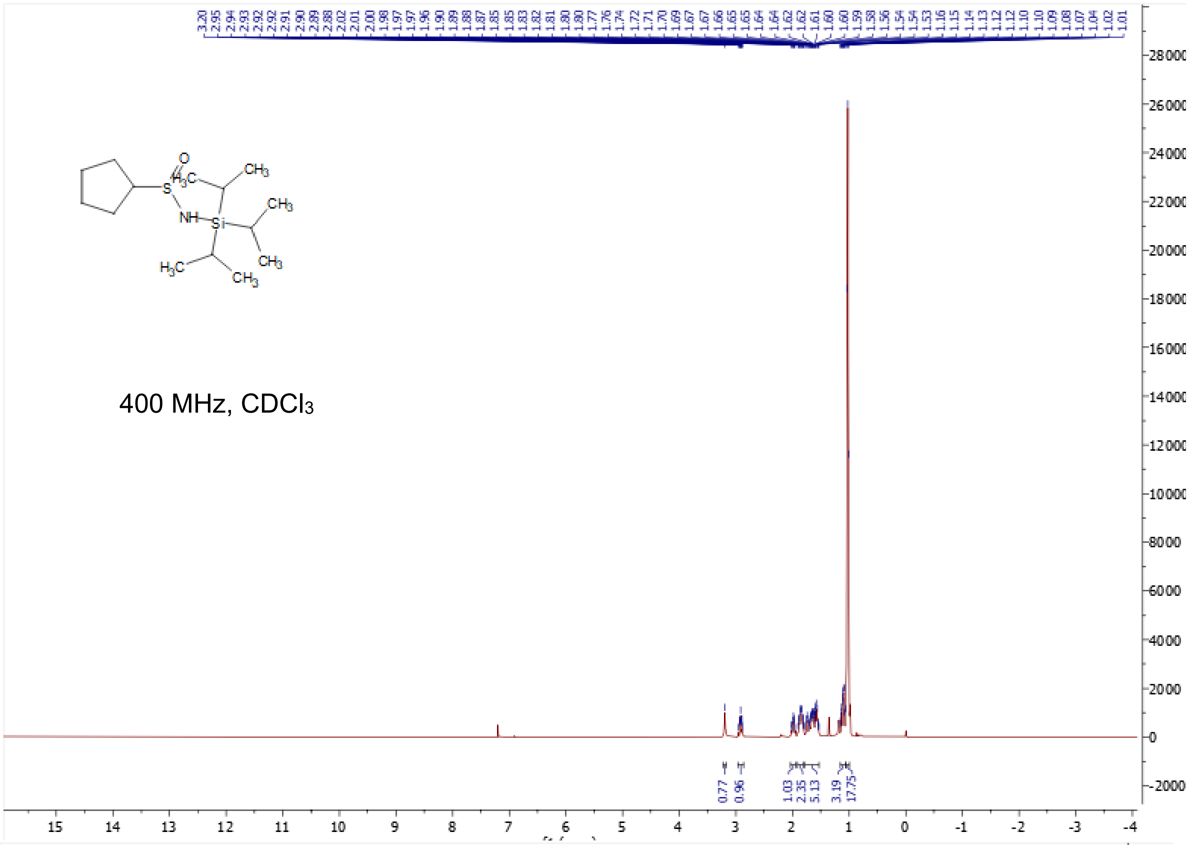

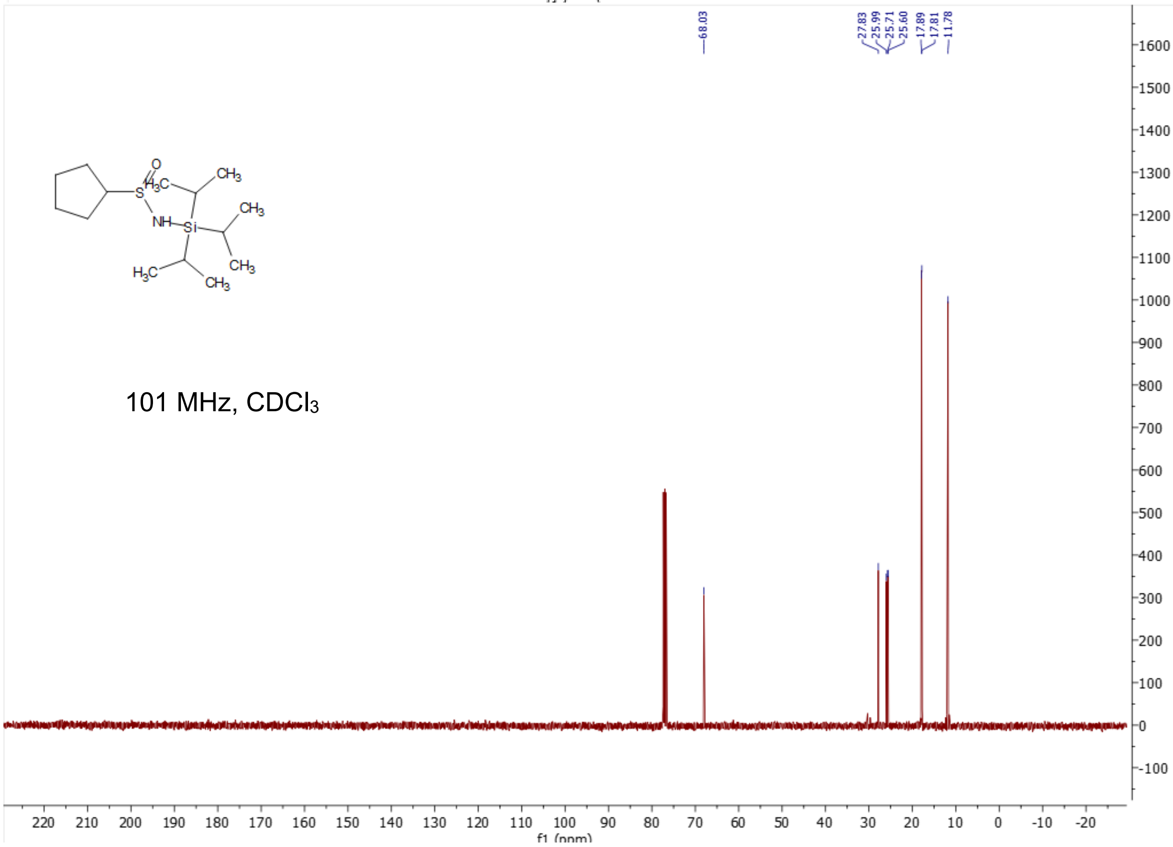

N-(Triisopropylsilyl)cyclohexanesulfinamide 3g

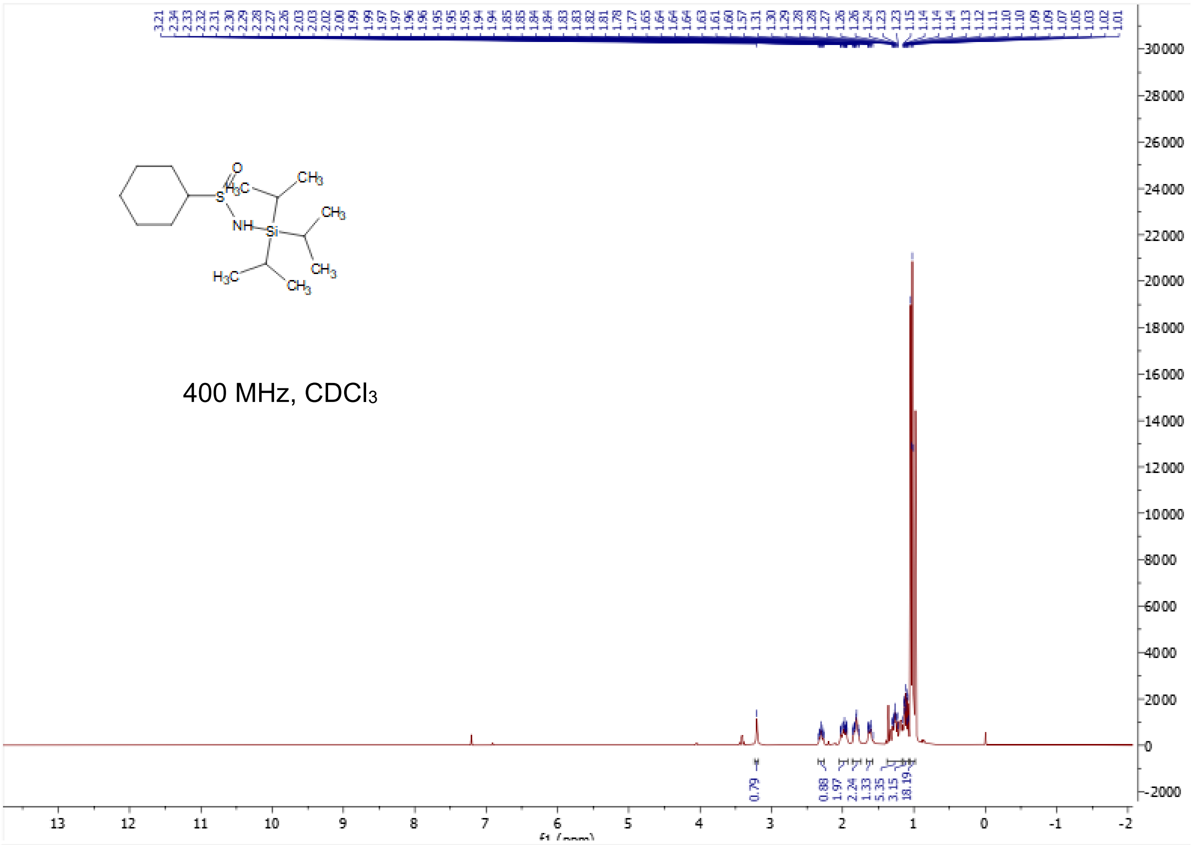

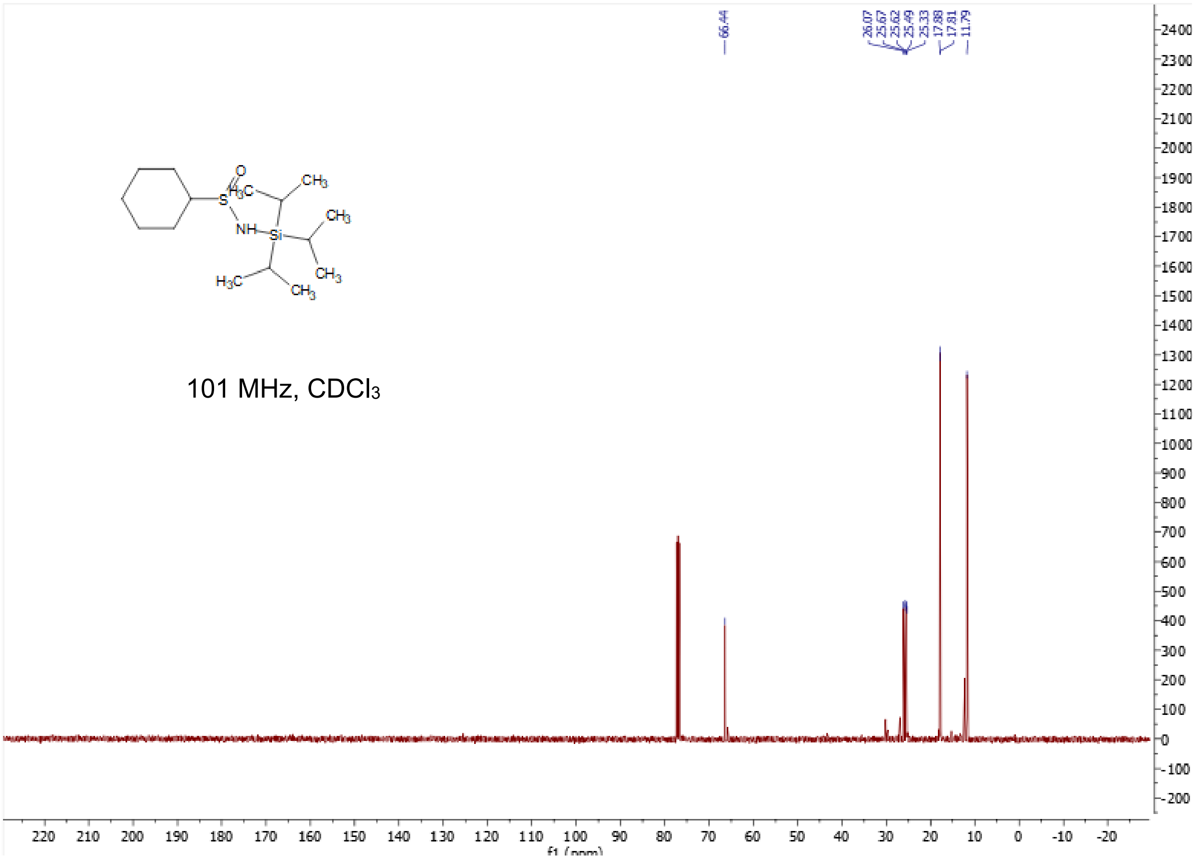

2-Methyl-*N*-(triisopropylsilyl)propane-2-sulfinamide 3h

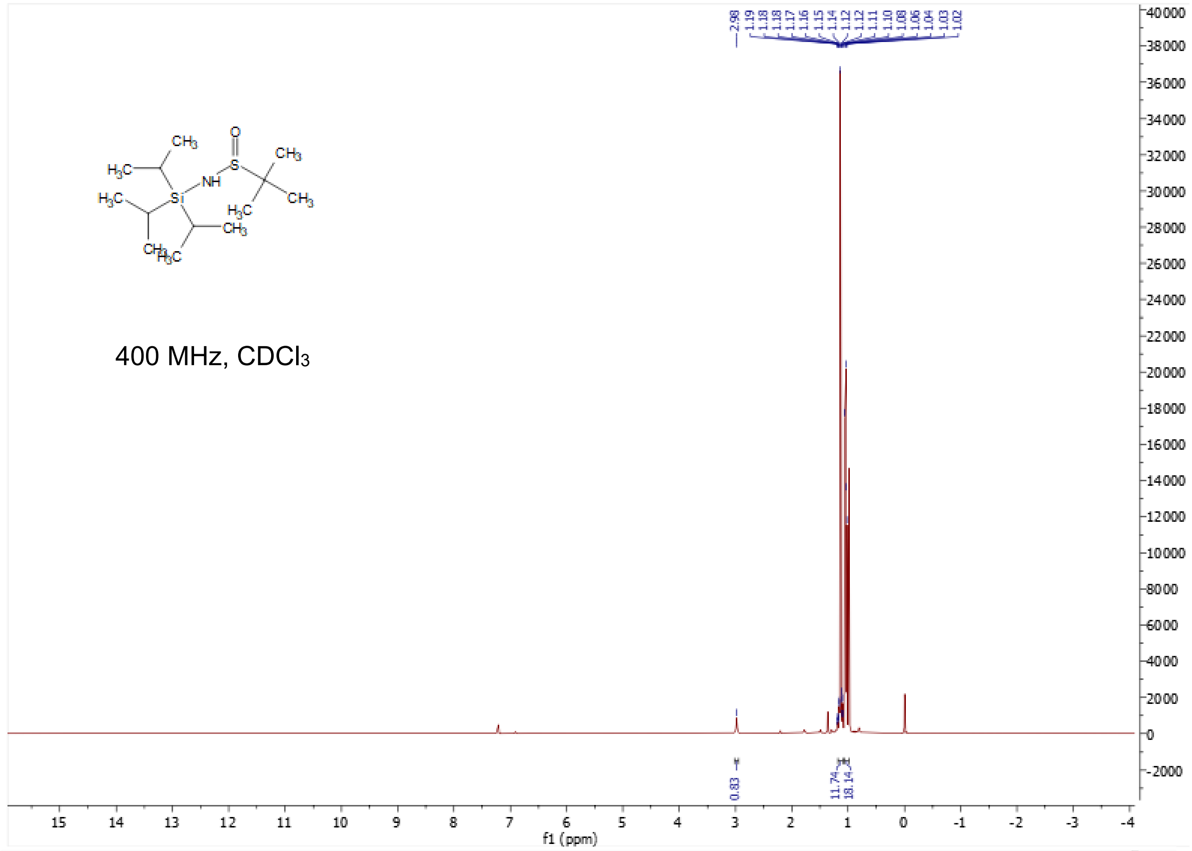

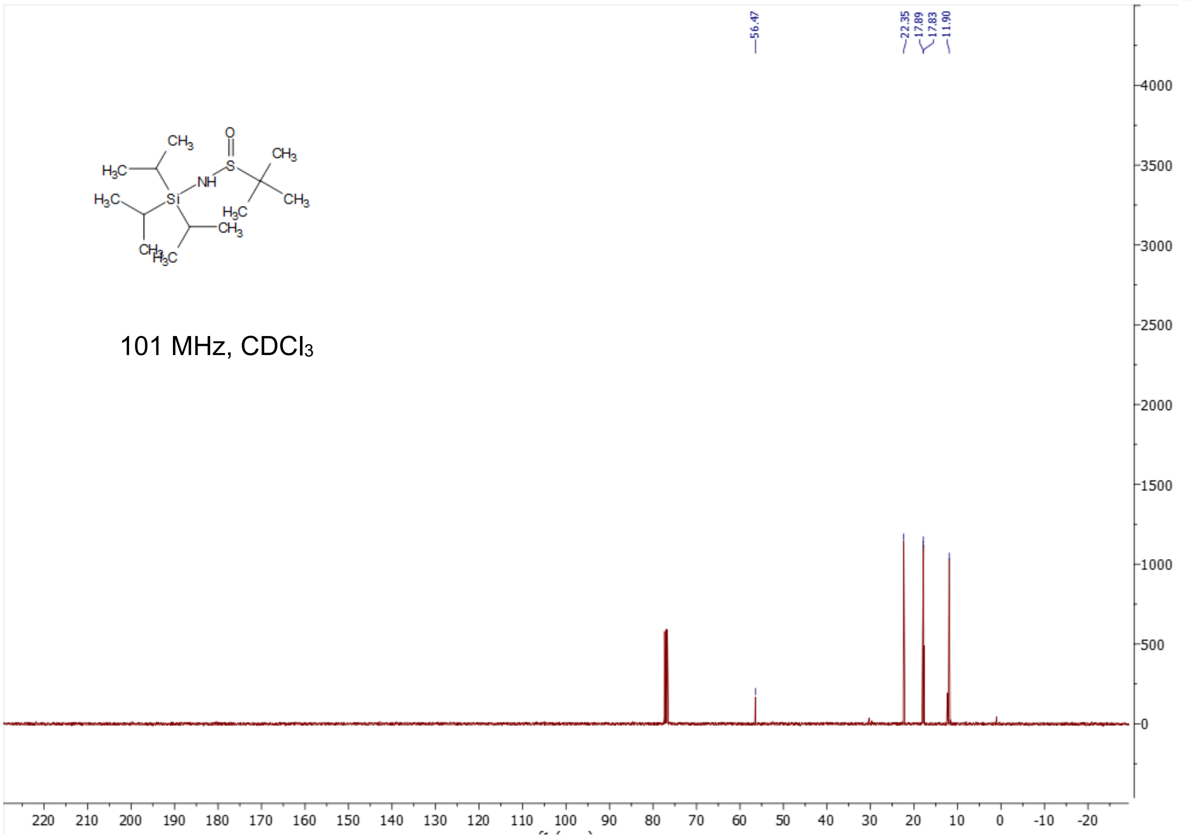

tert-butyl (tert-butoxycarbonyl)(((triisopropylsilyl)amino)sulfinyl)-D-alaninate 3i

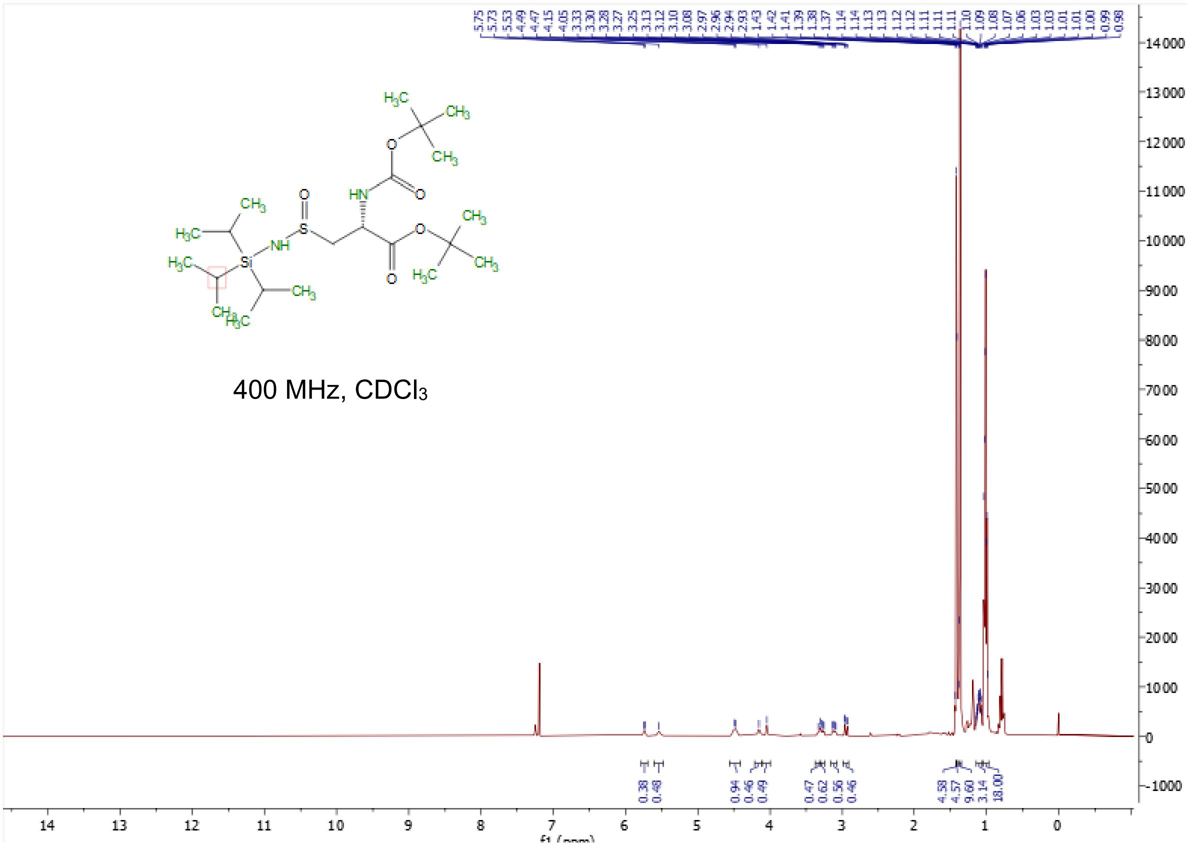

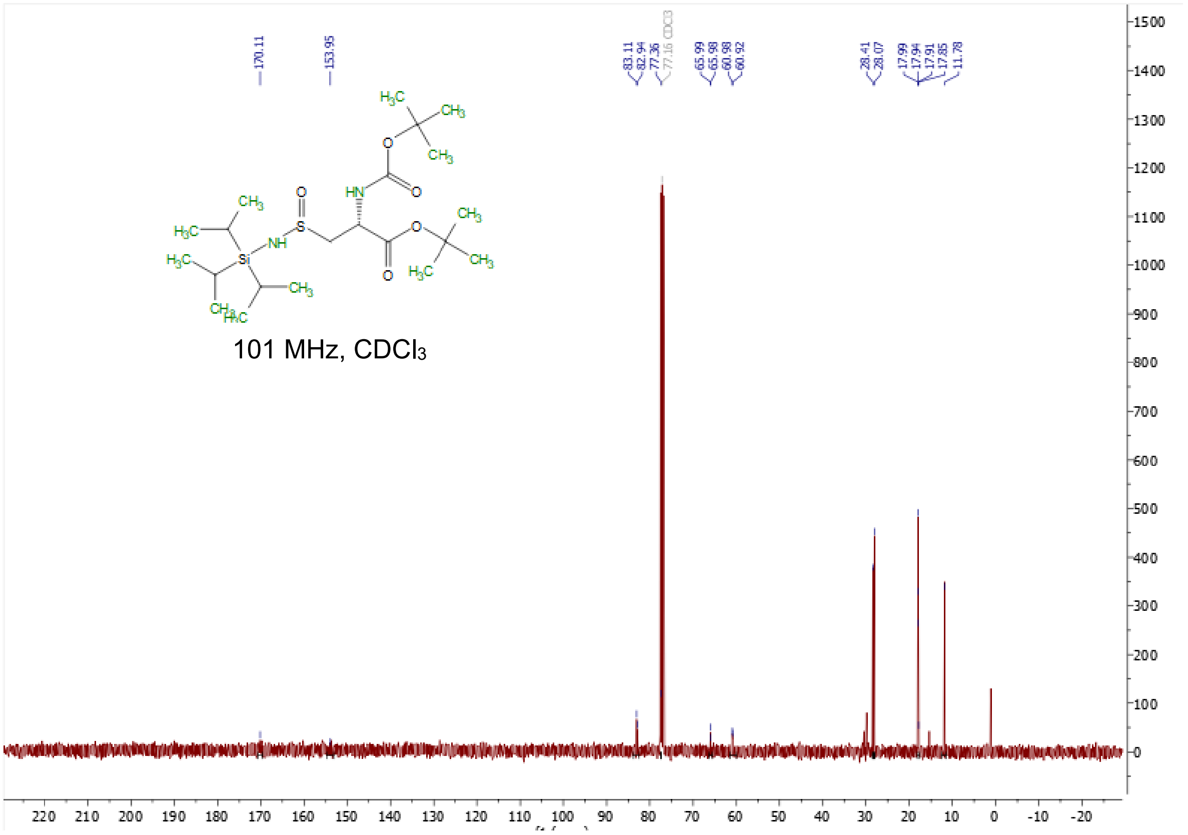

tert-butyl (2S)-2-((tert-butoxycarbonyl)amino)-4-(((triisopropylsilyl)amino)sulfinyl)butanoate 3j

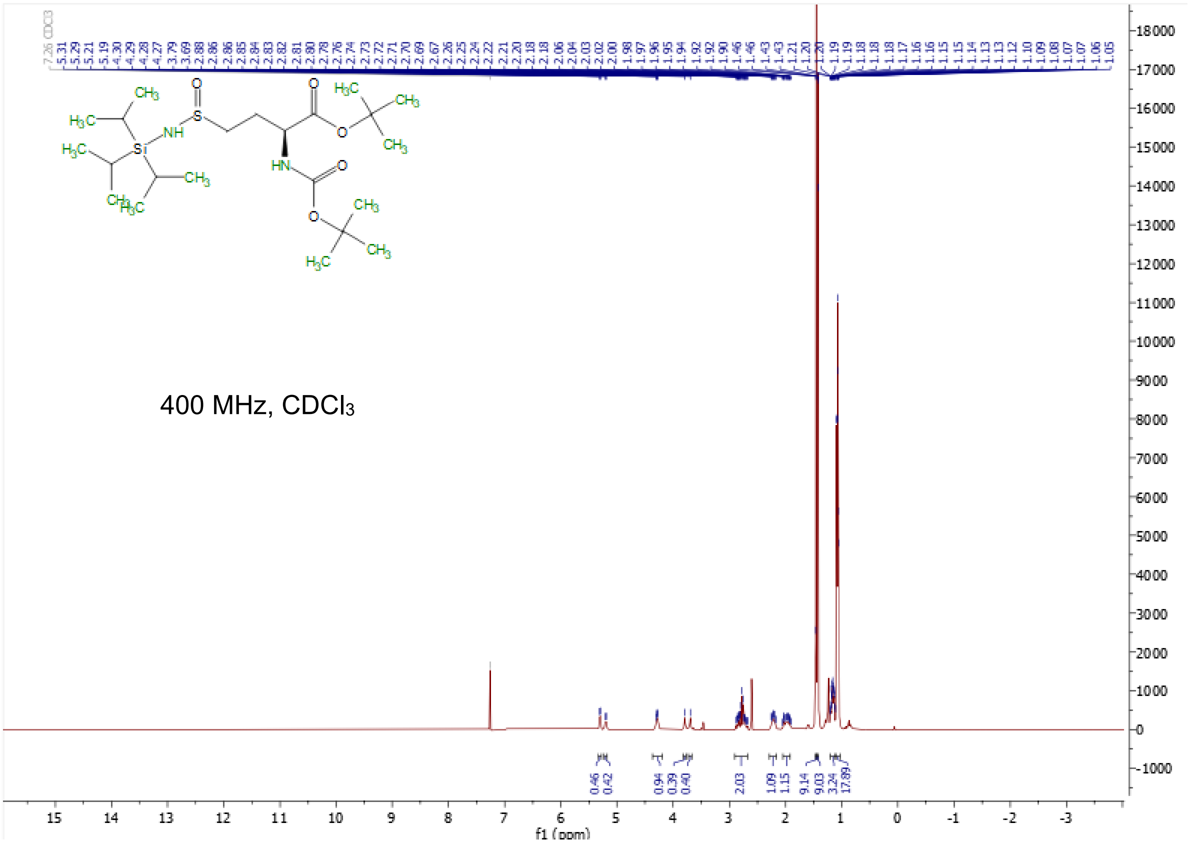

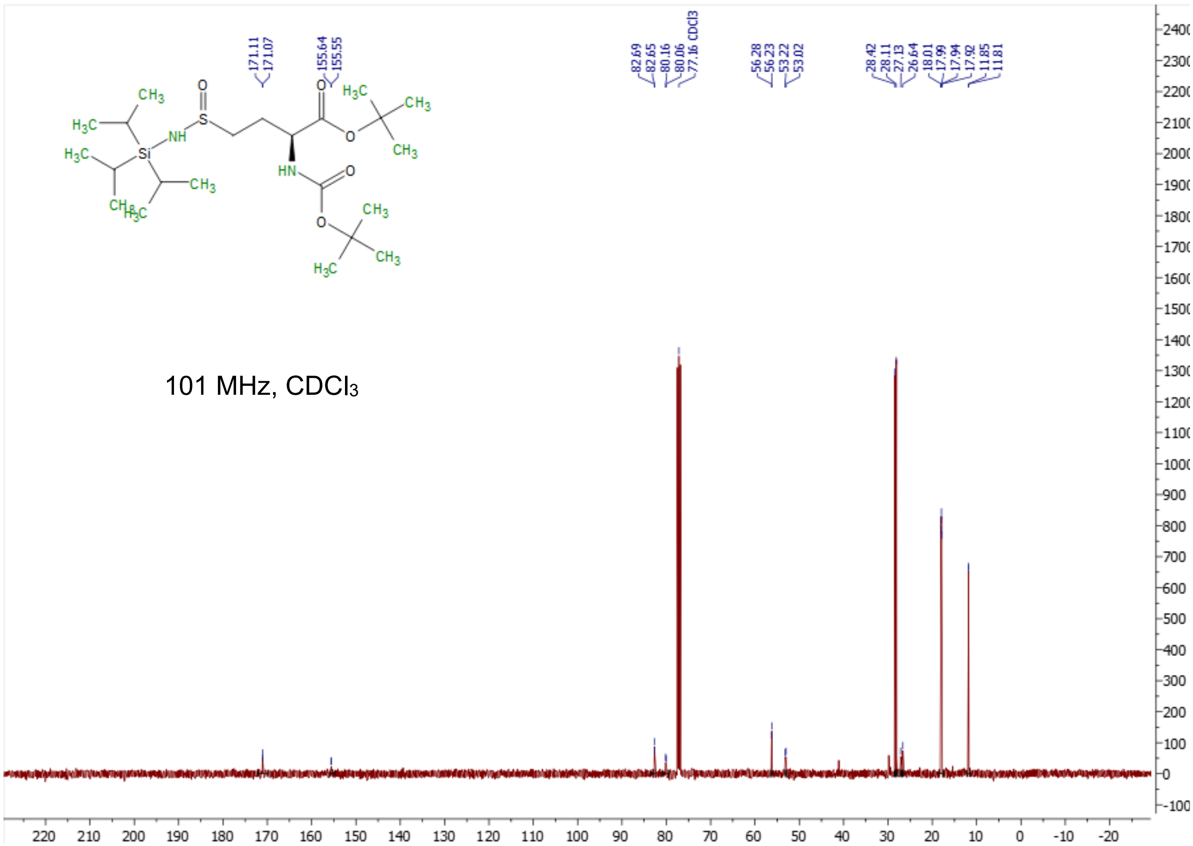

tert-butyl 4-(5-(3-(((triisopropylsilyl)amino)sulfinyl)bicyclo[1.1.1]pentan-1-yl)-1,2,4-oxadiazol-3-yl)piperidine-1-carboxylate **3k**

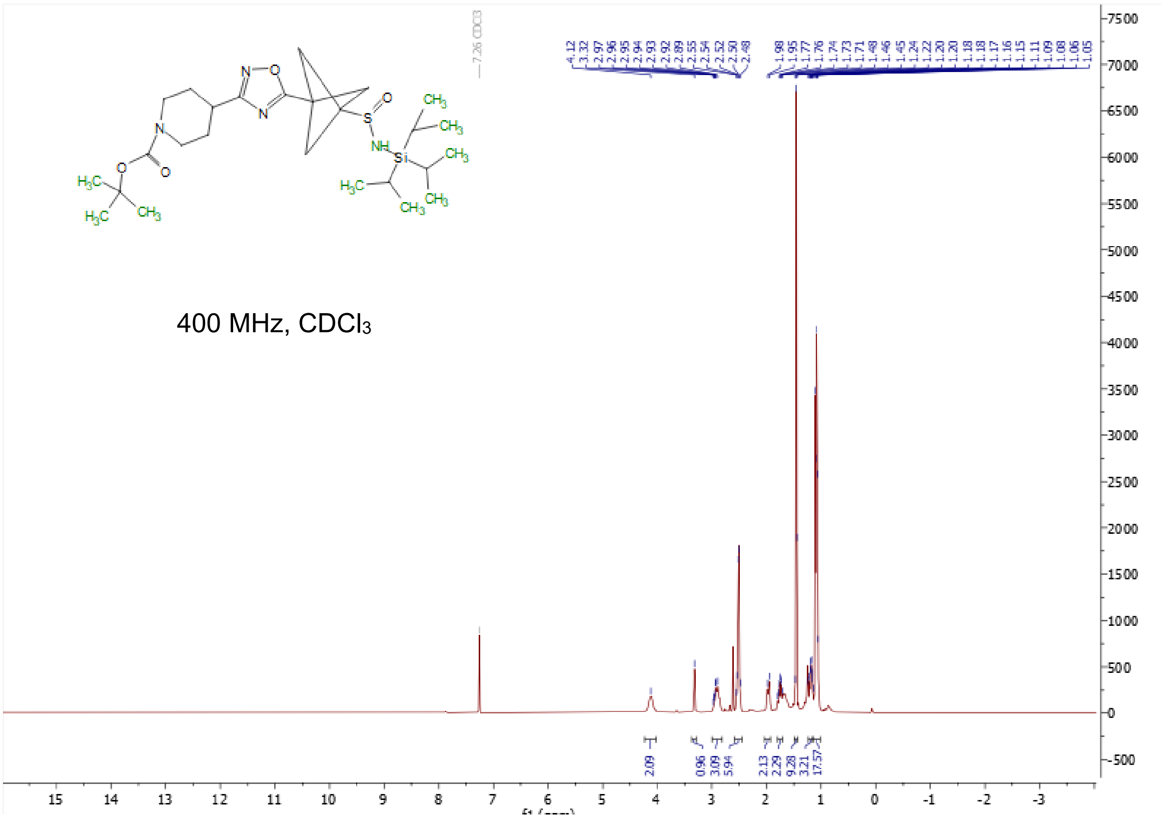

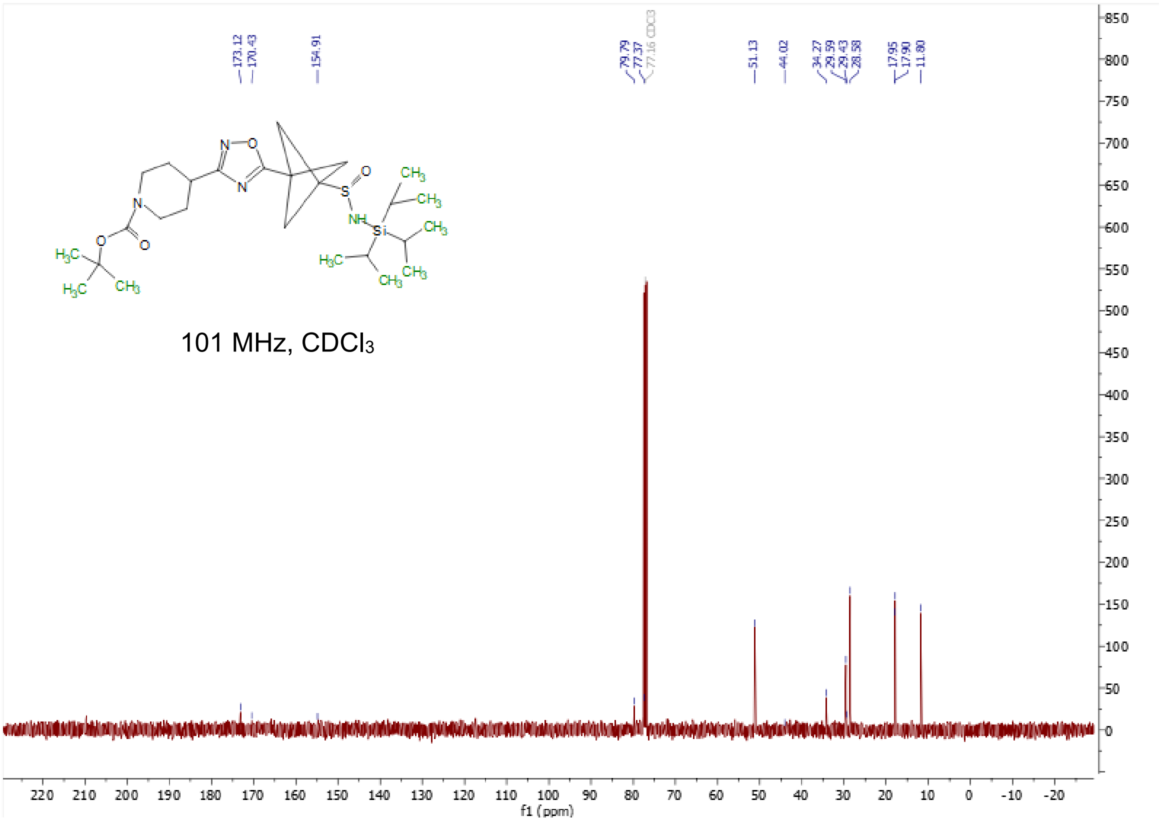

Tert-butyl 4-(5-(3-(S-fluoro-*N*-tritylsulfonimidoyl)bicyclo[1.1.1]pentan-1-yl)-1,2,4-oxadiazol-3-yl)piperidine-1-carboxylate 5

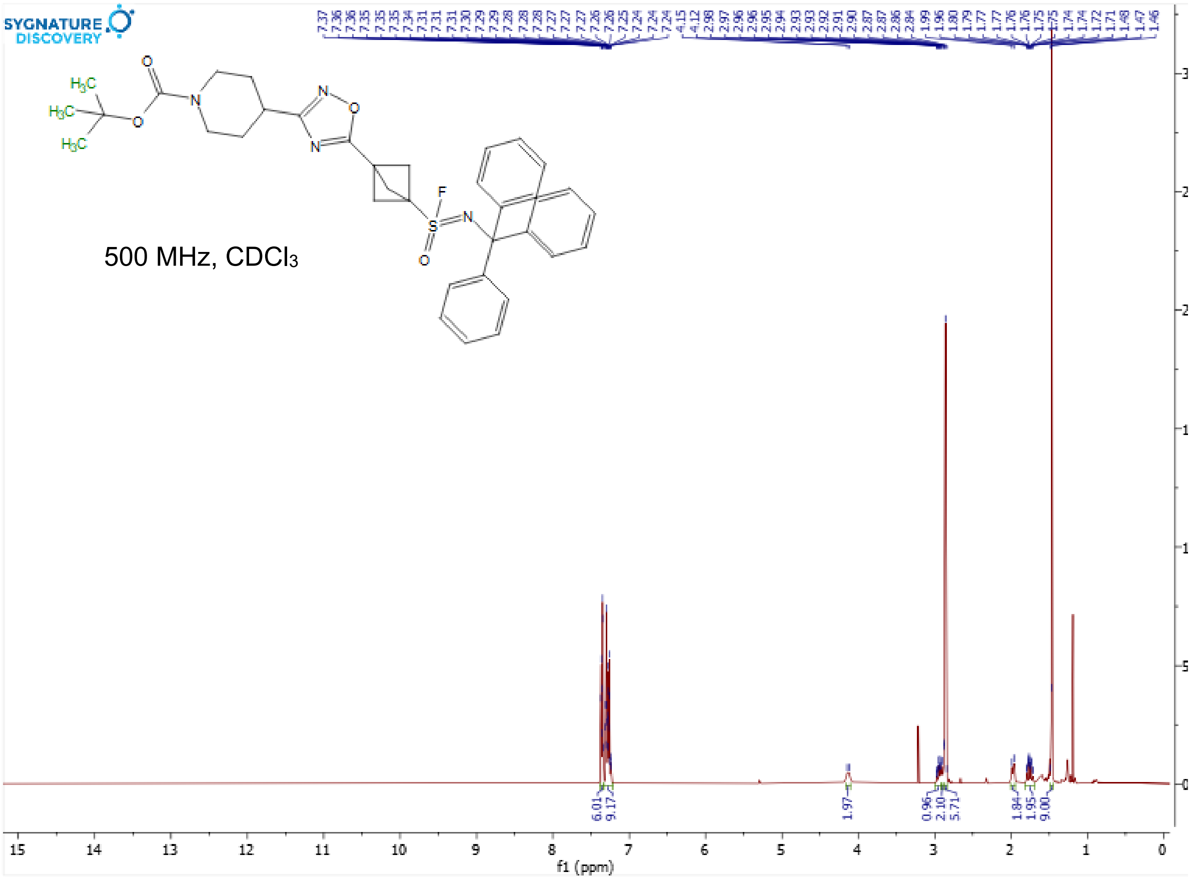

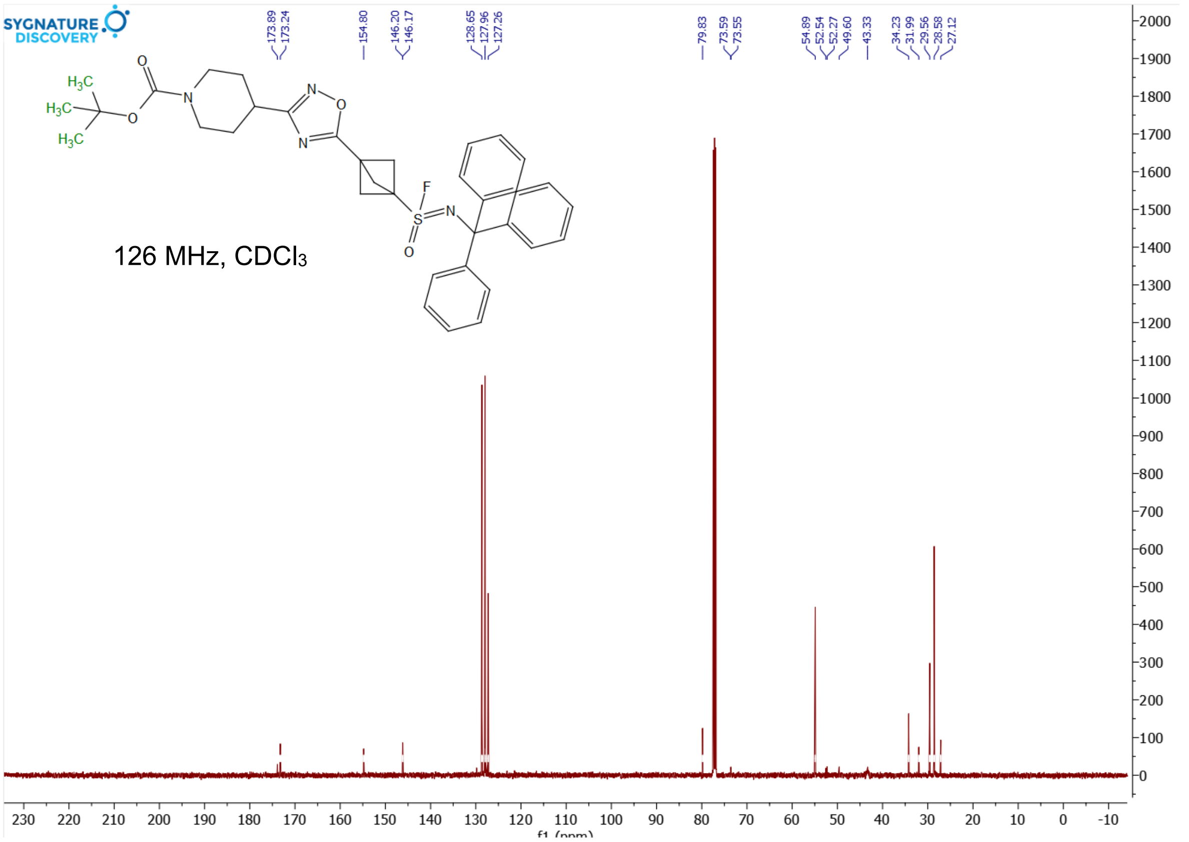

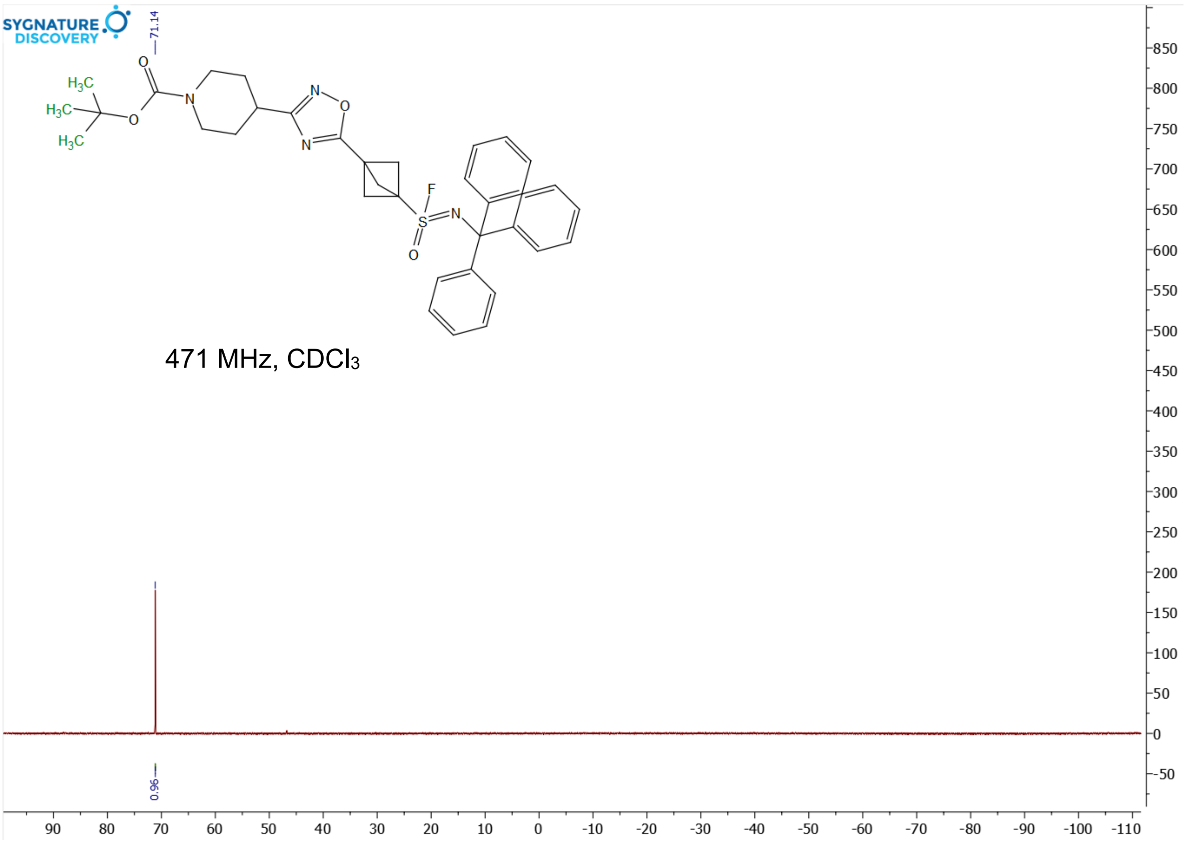
SYGNATURE DISCOVERY
71.14
471 MHz, CDCl3
0.96

Tert-butyl 4-(3-(3-(*N*-trityl-1H-imidazole-1-sulfonimidoyl)bicyclo[1.1.1]pentan-1-yl)-1,2,4-oxadiazol-5-yl)piperidine-1-carboxylate 6

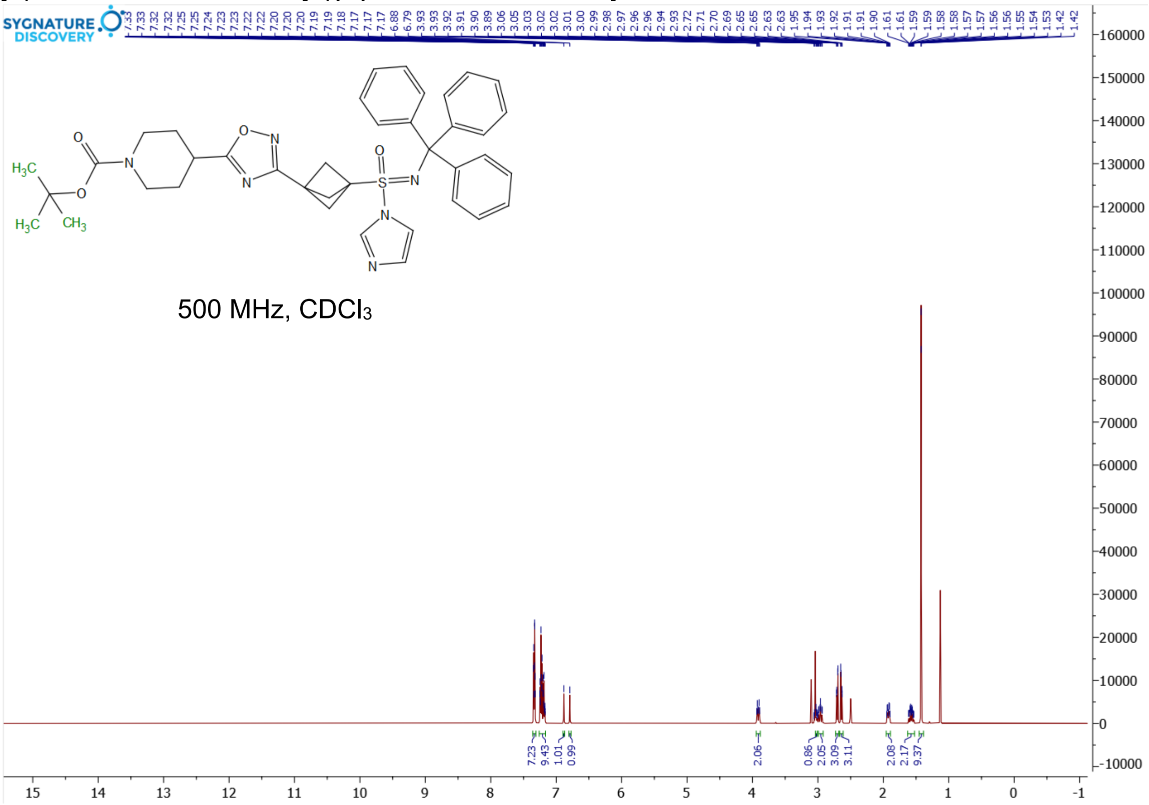

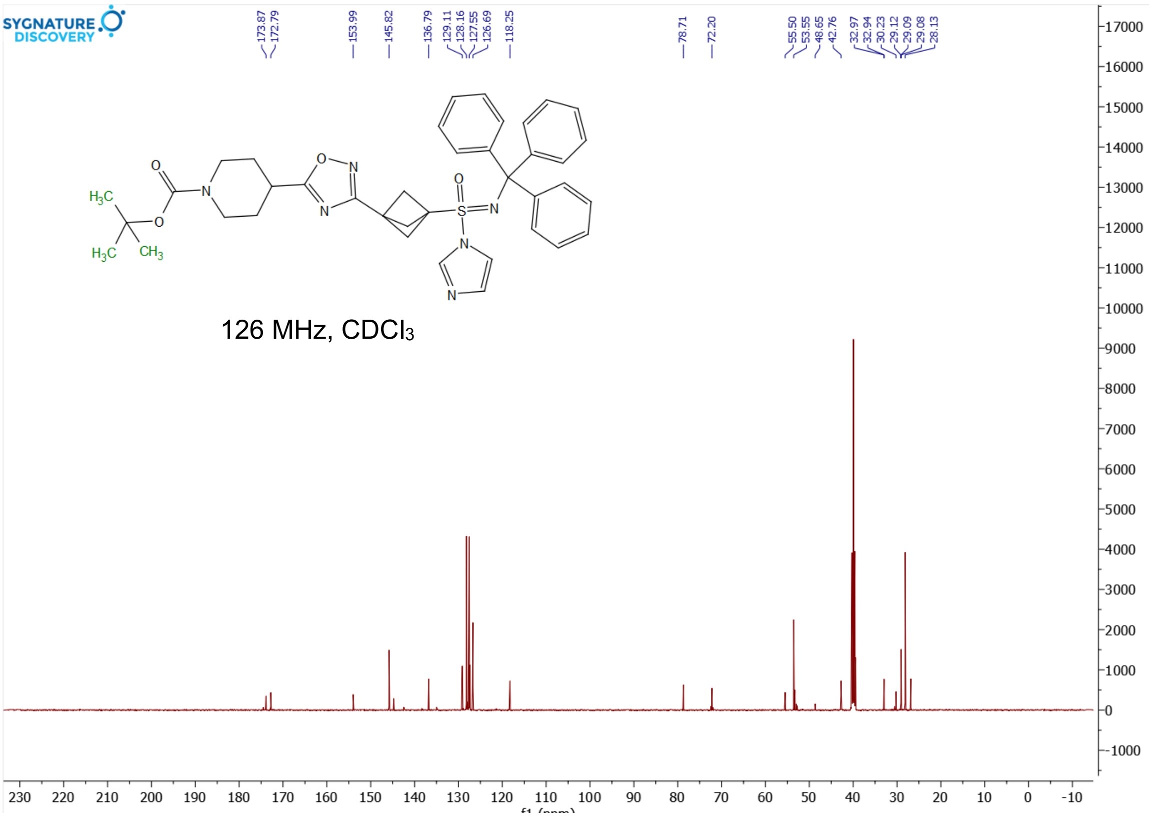

tert-butyl 4-(5-(3-(*N*-tritylsulfamoyl)bicyclo[1.1.1]pentan-1-yl)-1,2,4-oxadiazol-3-yl)piperidine-1-carboxylate 7

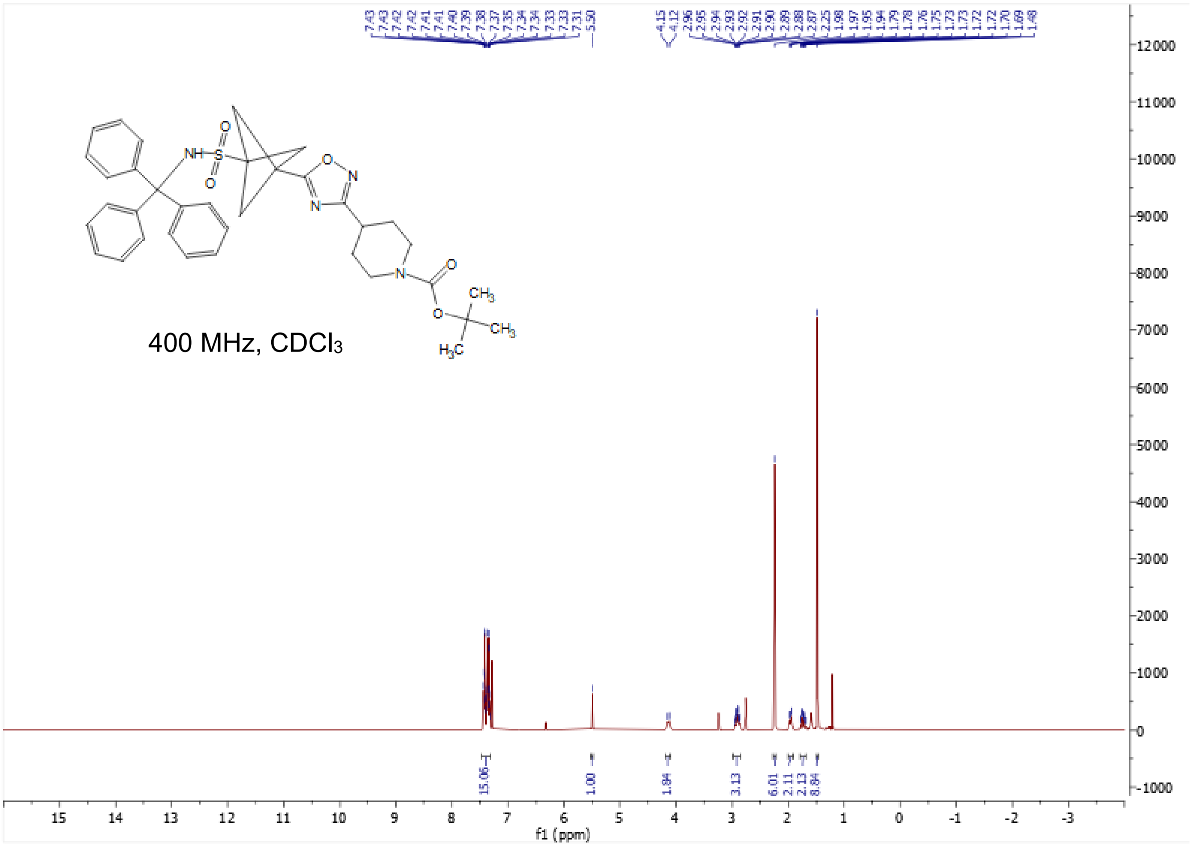

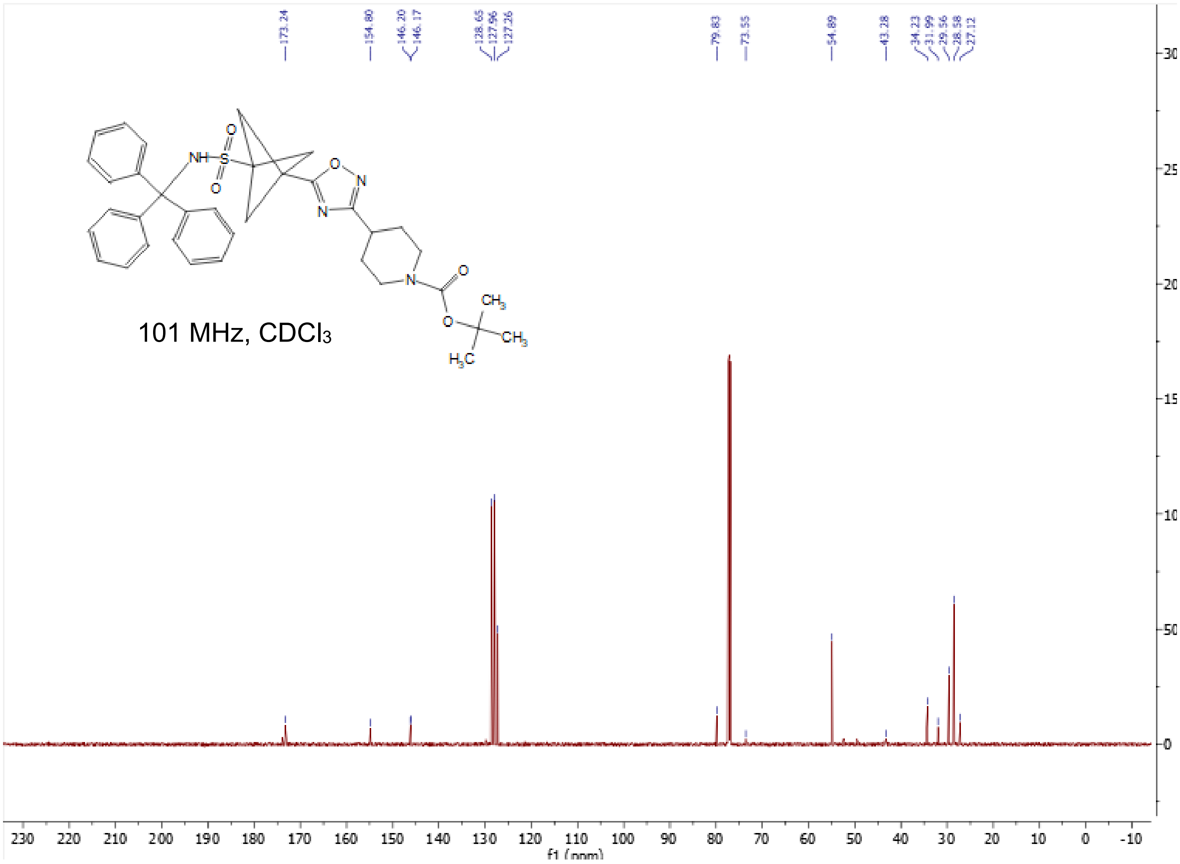

Supplement: Supplementary file 1 [file ol5c03100_si_001.pdf]
